# A C–H Activation Approach to the Tricyclic Core of Glionitrin A and B

**DOI:** 10.1021/acsomega.2c00810

**Published:** 2022-04-04

**Authors:** Nicolas R. Koning, Daniel Strand

**Affiliations:** Centre for Analysis and Synthesis, Department of Chemistry, Lund University, Box 124, SE-221 00 Lund, Sweden

## Abstract

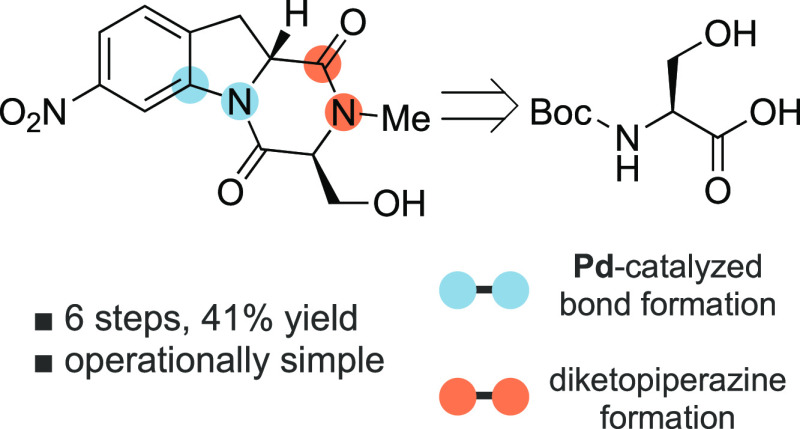

Synthesis of diketopiperazines has been of long-standing interest in both natural product synthesis and medicinal chemistry. Here, we present an operationally convenient and efficient approach to the fused indoline-diketopiperazine tricyclic core of glionitrin A/B and related structures using a Pd-catalyzed C–H activation reaction to form the indoline five-membered ring. Exploratory work aimed at elaborating the tricyclic structures into the corresponding natural products is discussed.

## Introduction

**Figure 1:**
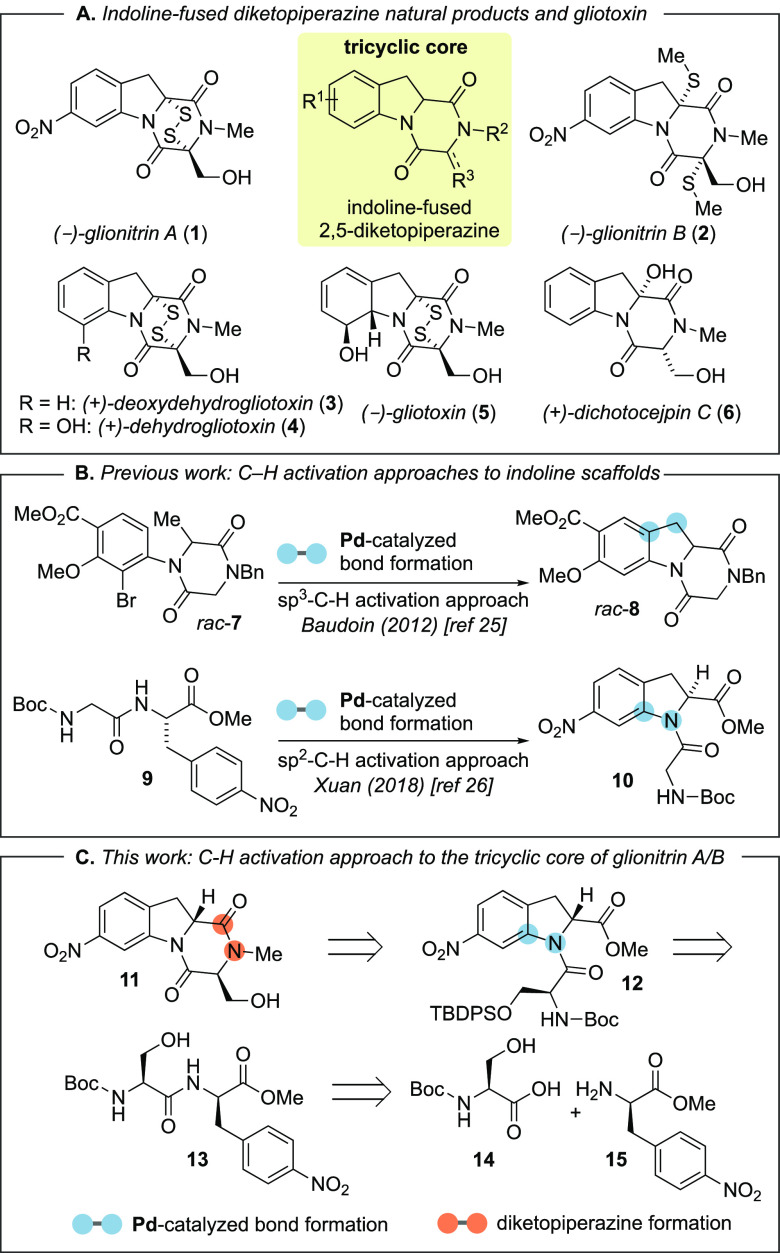
(A) Selected natural products; (B) selected previous C–H activation approaches to indoline-fused scaffolds; and (C) retrosynthesis of the tricyclic core of glionitrin A and B.

2,5-Diketopiperazines (DKPs) are ubiquitously found in natural products, materials, and drugs.^1,2^ Consequently, this motif has been the subject of extensive synthetic efforts.^2−4^ Natural products containing dithiodiketopiperazines (DTDKPs) have drawn particular interest due to their challenging syntheses and broad spectra of useful biological properties.^5,6^ A subclass of this natural product family comprises compounds wherein the DTDKP is fused to an indoline at the 2,3-positions. Such structures have received less synthetic attention than, for instance, the related non-aromatic gliotoxin (**5**)^7,8^ (Figure 1A). Examples include (−)-glionitrin A (**1**)^9^ and B (**2**),^10^ but the motif is also shared in aromatic gliotoxin homologues like (+)-deoxydehydrogliotoxin (**3**)^11^ and (+)-dehydrogliotoxin (**4**).^12^

The first syntheses of (−)-glionitrin A and B were recently reported by our group.^13^ In this study, the DTDKP motif was constructed in a stepwise manner outgoing from a triketopiperazine precursor. Still, a more direct approach would be to form the disulfide bridge on a DKP scaffold.^14^ Practical access to a suitably functionalized substrate of this type is thus of considerable interest for exploring new synthetic pathways to indoline-fused DTDKPs. Moreover, the glionitrins were originally isolated from a co-culture of two microbes, the bacterial strain *Sphingomonas* KMK-001 and the fungal strain *Aspergillus fumigatus* KMC-901, and details of their biosyntheses remain unclear.^9,10^ It seems plausible that DKP derivatives like **11** (Figure 1C) would lie on the biosynthetic pathway,^15^ and access to such compounds may aid in shedding light on the biosynthetic machineries that produce these fascinating structures.

Here, we describe a C–H activation approach leading to concise and practical syntheses of the complete tricyclic cores of glionitrin A/B and dehydrodeoxygliotoxin with defined stereochemistry. Exploratory studies toward elaborating these structures to the corresponding natural products by direct S–S bridge construction or via α-oxidation reactions are also discussed.

Indoline-fused DKPs have been synthesized through several approaches.^16−27^ Variations drawing on metal-catalyzed bond activation for forming the indoline five-membered ring are particularly attractive as these allow for simple starting materials.^22−26^ An elegant example is the palladium-catalyzed sp^3^-C–H activation approach by Baudoin and co-workers to form indoline **8** from DKP **7** (Figure 1B),^25^ which has since been applied to total syntheses of several DTDKP natural products.^28,29^ More recently, Xuan and co-workers described a palladium-catalyzed sp^2^-C–H activation to produce **10**, wherein the amide serving as a directing group for the C–H activation step is also incorporated in the formed ring.^26^

For the purpose of developing practical syntheses of the tricyclic cores of the glionitrins and homologous structures, we envisioned expanding on Xuan’s approach^26^ by elaborating the more functionalized dipeptide **13** to indoline **12** (Figure 1C). From this structure, tricyclic **11**, carrying the hydroxymethyl side chain of the glionitrins, would be completed by cyclization to a DKP followed by N-methylation and deprotection.

## Results and Discussion

**Scheme 1:**
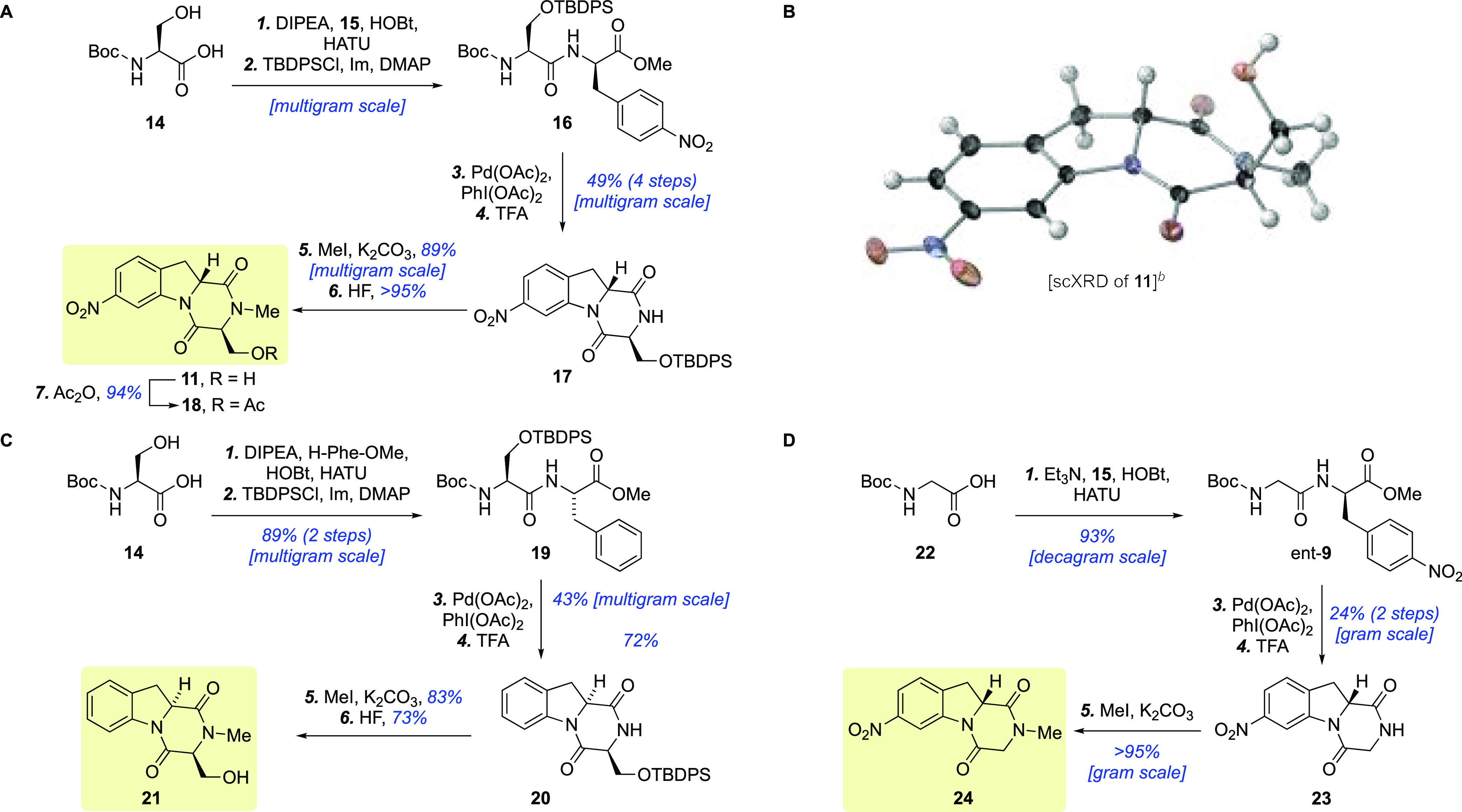
(A) Synthesis of the Tricyclic Core of Gliontrin A and B; (B) scXRD Structure of DKP **11**; (C) Synthesis of Tricyclic Structure **21**; and (D) Synthesis of Tricyclic Structure **24**^,^ Reagents and conditions: (1) **15**^.^HCl (1.05 equiv) or H-Phe-OMe^.^HCl (1.1 equiv), HATU (1.0–1.2 equiv), HOBt (1.0–1.2 equiv), Et_3_N (4.0 equiv) or DIPEA (4.0 equiv), CH_2_Cl_2_, 0 °C to rt, 15–21 h; (2) TBDPSCl (1.1–1.2 equiv), Im (1.2–1.4 equiv), DMAP (10 mol %), CH_2_Cl_2_, 0 °C to rt, 90–105 min; (3) Pd(OAc)_2_ (5 mol %), PhI(OAc)_2_ (2.0 equiv), toluene, reflux, 16–24 h; (4) TFA (excess), CH_2_Cl_2_, 0 °C to rt, 0.75–3 h; (5) K_2_CO_3_ (25 equiv), MeI (excess), acetone, rt or reflux, 6–7 d; (6) HF (aq, 48% w/w, excess), pyridine, rt, 1–20 h. (7) acetic anhydride, 100 °C, microwave irradiation, 2 h. boc = *tert*-butyl carbamate, DMAP = 4-(dimethylamino)pyridine, Im = imidazole, TBDPSCl = *tert*-butyldiphenylsilyl chloride, DIPEA = *N*,*N*-diisopropylethylamine, HATU = 1-[bis(dimethylamino)methylene]-1*H*-1,2,3-triazolo[4,5-*b*]pyridinium 3-oxid hexafluorophosphate, and HOBt = 1-hydroxybenzotriazole; thermal ellipsoids are shown at 50% probability.

We first targeted protected dipeptide **16** as a suitable cyclization precursor. Synthetically, *N*-boc-protected serine **14** was coupled with nitro-phenylalanine **15** using HATU (Scheme 1A). Protection of the free alcohol was needed to avoid side reactions such as C–O bond cleavage^30^ or oxidation^31^ during the subsequent C–H activation reaction. A *tert*-butyldiphenyl silyl (TBDPS) group was found suitable for this purpose and also proved beneficial by improving the solubility of intermediates. We initially explored cyclization of a dipeptide substrate with the requisite *N*-methyl group already installed. However, in agreement with observations by Xuan,^26^ this derivative was found incompatible with the C–H activation step due to the inability to form a bidentate coordination with the metal.

With **16** in hand, the stage was set for investigating the C–H activation step to form the indoline five-membered ring of the targeted tricyclic system. To this end, we employed Xuan’s optimized conditions:^26^ Pd(OAc)_2_ (5 mol %) and PhI(OAc)_2_ (2.0 equiv) in refluxing toluene. Pleasingly, indoline **12** could be isolated along with recovered starting material in a 2:1 ratio. A brief optimization of the reaction conditions showed that increasing the amount of PhI(OAc)_2_ to 4.0 equiv improved the yield to 50% (see Supporting Information, Table S1), but for gram-scale synthesis, we settled on the original protocol.

Due to the difficult separation of the mixture of **16** and **12** obtained in the C–H activation step, the products were directly subjected to trifluoroacetic acid (TFA) to give DKP **17** in good overall yield (49% over four steps). It is worth noting that the sequence of reactions starting from commercially available **14** could be telescoped without chromatographic purification of intermediates to produce >9 g of **17** in a single pass.

Next, we turned our attention to N-methylation of amide **17**. This transformation proved challenging due to competing β-siloxy elimination in presence of strong bases like NaH or NaHMDS. Ultimately, we found that amide **17** was smoothly methylated using an excess of MeI with K_2_CO_3_ as the base^32^ to give *N*-methyl DKP **30** in 89% yield. Synthesis of the tricyclic core of glionitrin A and B (**11**) was then completed by liberation of the primary alcohol with HF (>95% yield). The structure of alcohol **11** was confirmed by single crystal X-ray diffraction (scXRD) analysis (Scheme 1B). The conversion of **11** into the corresponding acetate **18** was also straightforwardly accomplished in 94% yield by reaction with acetic anhydride under microwave irradiation for 2 h at 100 °C (Scheme 1A)

We then applied the same approach to diastereomeric des-nitro derivative **21** (Scheme 1C). This structure was obtained in six steps from *N*-boc-protected serine **14** in 17% overall yield. In a similar fashion, the corresponding glycine derivative **24** was also produced in 21% yield over four steps (Scheme 1D).

**Scheme 2:**
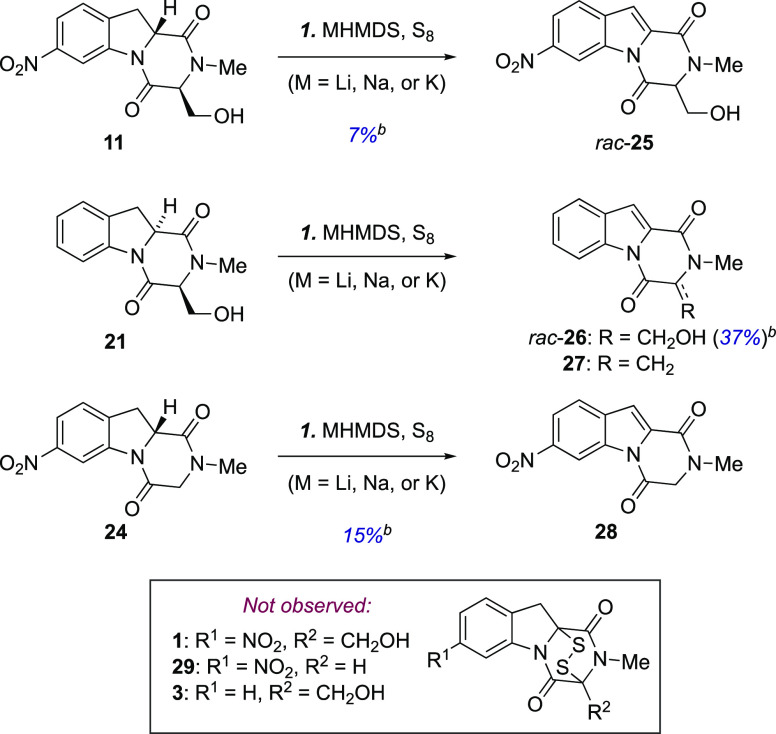
Attempted Direct Sulfenylation of DKPs **11**, **21**, and **24**^,^ Reagents and conditions: (1) NaHMDS, LiHMDS, or KHMDS (5.0–16 equiv), S_8_ (1.0–2.0 equiv), THF, −78 °C or room temperature, 1.5–3 h; using LiHMDS.

With a practical method to access to the tricyclic cores of the glionitrins and two related structures in place, we explored methods aimed at elaborating these to the corresponding epi-DTDKPs.^8,22,28,29,33−35^ To this end, we first investigated direct installation of the disulfide bridge by reacting DKP enolates with electrophilic sulphur.^8,14^ The reactions of unprotected **11**, **21**, and **24** or the protected **30** and **35** using various bases (LiHMDS, NaHMDS, or KHMDS) and elemental sulfur (S_8_) as the electrophile precursor^8,14^ following literature procedures^8^ gave slightly varying results, but in no case was formation of the corresponding epi-DTDKP products observed as evident by comparison of the crude ^1^H NMR spectra with those of known structures (Scheme 2).

In a brief summary, reacting alcohol **11** with LiHMDS (8.0 equiv) and S_8_ (1.0 equiv) with the intent of forming glionitrin A gave a mixture of indole **25** along with recovered starting material. Switching the base to NaHMDS or KHMDS gave more complex mixtures of unidentified side products together with indole **25** and unreacted **11**. Similar results were obtained when switching the order of addition [LiHMDS (4.0 equiv) first and a mixture of LiHMDS (4.0 equiv)/S_8_ (1.0 equiv) second], decreasing the reaction temperature (−78 °C), or doubling the amounts of reagents [LiHMDS (16.0 equiv) and S_8_ (2.0 equiv)]. Indole formation was also observed when exposing des-nitro indoline **21** or indoline **24** to the same conditions. In the reaction of **21** with LiHMDS (8.0 equiv) and S_8_ (1.0 equiv), we also observed olefin **27** as a minor side product. Reactions of the corresponding O-silylated substrates under the same conditions followed a similar pattern: using LiHMDS, NaHMDS, or KHMDS as the base, silyl ethers **30** and **35** were both converted into complex mixtures.

Although examples of successful formation of epi-DTDKPs on indoline-fused DKP scaffolds have been reported using the here-evaluated procedures in moderate to good yields,^8,14,36^ our results suggest that substrates like **11** are not amenable to these conditions and that the encountered difficulties are not easily circumvented by variations in reaction conditions and operational procedures.

**Scheme 3:**
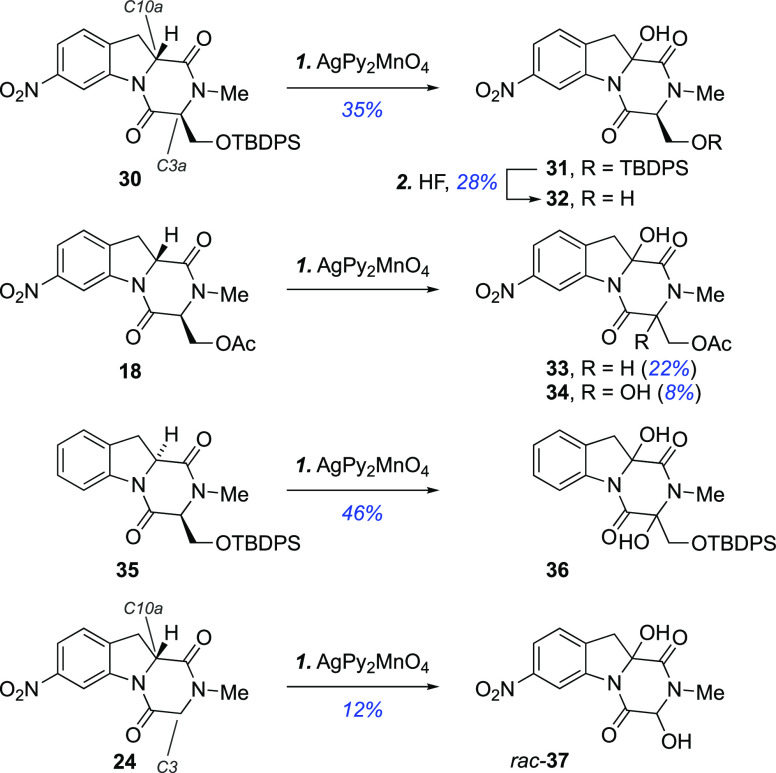
α-Oxidation of DKPs **18**, **24**, **30**, and **35** Reagents and conditions: (1) silver(I)bispyridine permanganate (4.0–8.0 equiv), CH_2_Cl_2_, rt, 15–24 h; (2) HF^.^py, pyridine, rt, 2 h.

An alternative approach to construct disulfide bridges on DKP scaffolds is the biomimetic α-oxidation with permanganate reagents pioneered by Movassaghi.^32,37−42^ The hemi-aminal DKPs formed with this procedure have been successfully transformed into highly complex natural product targets via acid-promoted sulfenylation.^32,37,38^ Inspired by this work, we explored oxidation of DKPs **18**, **24**, **30**, and **35** with silver(I)bispyridine permanganate (Scheme 3).^43^

Thus, oxidation of silyl ether **30** with silver(I)bispyridine permanganate (8.0 equiv) in CH_2_Cl_2_ at room temperature gave a difficult to separate 40:60 mixture of unreacted starting material and C10a mono-oxidized **31** as a single diastereomer (35% yield). Trace amounts of the C3a/C10a doubly oxidized product were detected by HRMS analysis of the crude reaction mixture. Treatment of the mixture of **30** and **31** with HF provided diol **32**.

Oxidation of acetate **18** with silver(I)bispyridine permanganate (8.0 equiv) was also evaluated and gave a mixture of C10a mono-oxidized **33** (22% yield) and C3a/C10a doubly oxidized **34** (8% yield). In contrast, des-nitro silyl ether **35**, which is less deactivated at the C3a position, reacted smoothly with silver(I)bispyridine permanganate (4.0 equiv) to produce diol **36** in 46% yield as a single observed diastereomer. Similarly, oxidation of indoline **24** produced diol **37**, albeit in only 12% isolated yield.

Assignment of the relative configuration for the oxidized products was attempted using 2D-NMR spectroscopy experiments; however, the results were inconclusive. For **31–34** and **36**/**37**, the differences in distance between the C10a hydroxyl group proton and protons at C3/C3a position are too small to unambiguously distinguish between the cis and trans configurations by NOESY correlations. For **32**, **34**, and **36/37**, a further complication was magnetization transferred between the alcohol protons due to chemical exchange.

**Scheme 4:**
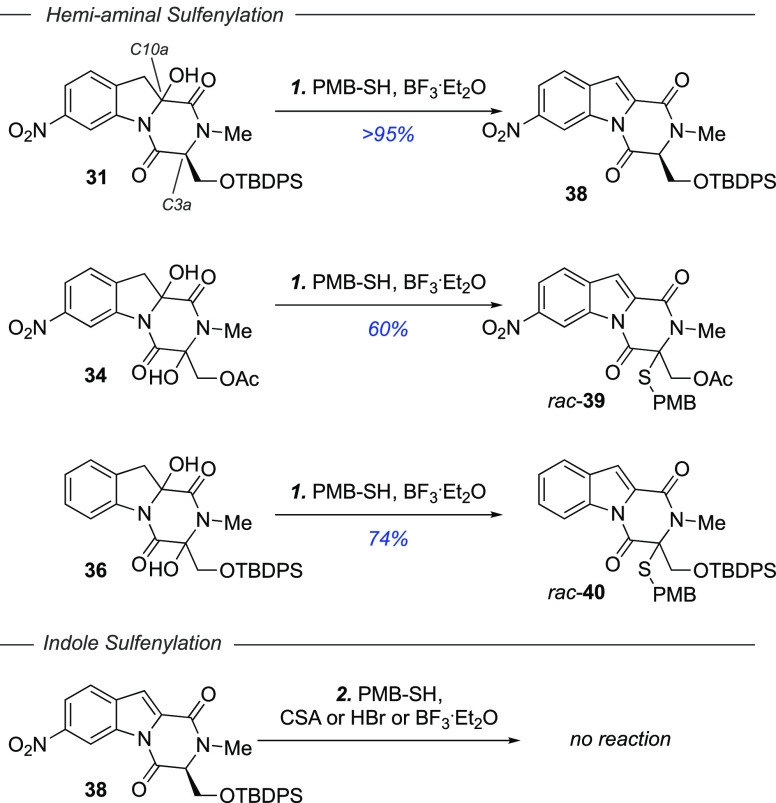
Sulfenylation of Hemi-Aminals **31**, **34**, and **36** and Attempted Sulfenylation of Indole **38** Reagents and conditions: (1) BF_3_·Et_2_O (20.0 equiv), 4-methoxy-α-toluenethiol (10.0 equiv), CH_2_Cl_2_, rt, 1 h; (2) *rac*-camphorsulfonic acid (10.0 equiv) or HBr (33% w/w in AcOH, excess) or BF_3_·Et_2_O (10.0 equiv), 4-methoxy-α-toluenethiol (4.0 equiv), CH_2_Cl_2_, reflux, 1 h.

Sulfenylation of the obtained hemi-aminals **31**, **34,** and **36** was investigated next (Scheme 4). Substrates with hemi-aminals at C10a cleanly eliminated into the corresponding indoles, whereas Lewis acid-promoted thio-aminal formation proved fruitful at the C3a position. The reaction of alcohol **31** with BF_3_·Et_2_O in the presence of 4-methoxy-α-toluenethiol thus gave indole **38** (>95% yield). Under the same reaction conditions, diol **34** was converted into C3a sulfenylated indole **39** (60% yield) and diol **36** into C3a sulfenylated indole **40** (74% yield).

Finally, we evaluated conversion of indole **38** to the corresponding C10a sulfenylated product with 4-methoxy-α-toluenethiol using various acids^44^ (camphorsulfonic acid, HBr, or BF_3_·Et_2_O). In all cases, **38** was recovered unreacted.

Thus, while the tricyclic substrates were successfully oxidized at C10a, and in case of **18**, **24**, and **35**, also at C3/C3a, the propensity to eliminate to the corresponding indoles was found prohibitive for the purpose of conversion to the corresponding epi-DTDKP natural products under the investigated conditions.

## Conclusions

In conclusion, a concise, simple, and scalable route to the complete indoline-fused DKP core of the glionitrins and related natural products has been developed. Xuan’s palladium-catalyzed C–H activation^26^ was applied to form the indoline five-membered ring from simple dipeptides which, in turn, were readily prepared from abundant and enantiomerically pure amino acids. The approach connects with putative intermediates in the biosyntheses of several natural products. Investigations aimed to elaborate the obtained tricyclic structures into their corresponding DTDKPs, either by direct S–S bridge construction or sulfenylation via oxidative conversion to the corresponding bis-hemi-aminals, were not met with success due to competing indole formation. The development of milder conditions for direct S–S bridge construction and for sulfenylation of C10a hemi-aminals thus remains an outstanding challenge with sensitive indoline-fused DKP scaffolds. An additional interesting question is that of absolute stereocontrol in such reactions. Studies aimed at addressing these problems are under way in our laboratory and will be reported in due course.

## Experimental Section

### General Procedures

All reactions were conducted in air unless otherwise stated. Reactions involving hydrogen fluoride were carried out in plastic vessels. The experiment involving microwave irradiation was performed using a Biotage Initiator+. All reagents and solvents were bought from commercial suppliers and used as received unless otherwise stated. Dichloromethane and toluene were obtained from a MBraun MB-SPS 800 solvent purification system. THF was distilled over sodium benzophenone ketyl. S_8_ was recrystallized from benzene. ^1^H and ^13^C (^1^H decoupled) NMR spectroscopy data were collected on a Bruker AVANCE II 400 MHz (^1^H 400 MHz; ^13^C 101 MHz) equipped with a 5 mm BBOF *Z*-gradient probe. Multiplicities are denoted by singlet (s), doublet (d), doublet of doublets (dd), doublet of doublet of doublets (ddd), triplet of doublets (td), triplet (t), apparent triplet (app. t), doublet of triplets (dt), and multiplet (m). Broad peaks are denoted by (br). IR spectra were recorded on a Bruker ALPHA II spectrometer and peaks denoted as strong (s), medium (m), weak (w), and broad (br). Optical rotations [α]_D_^T^ were recorded at room temperature (∼20 °C) using a PerkinElmer model 341 polarimeter. D represents the sodium D line (589 nm), and concentrations (*c*) are reported in g/100 mL. HRMS data were obtained using an ESI-QTOF mass spectrometer (Waters Xevo-G2) in the positive mode between *m*/*z* 50–1200, employing lockmass correction according to the manufacturer’s instructions. Thin layer chromatography (TLC) was performed using Merck 60 F_254_ silica gel bound to aluminum plates. Purification by column chromatography was performed using Merck 60 Å (40–63 μm particle size) silica or using the Biotage Isolera One system.

### Methyl (*R*)-2-((*S*)-2-((*tert*-Butoxycarbonyl)amino)-3-hydroxypropanamido)-3-(4-nitrophenyl)propanoate (**13**)

To a stirred suspension of serine **14** (7.61 g, 37.1 mmol) and nitrophenyl alanine **15**^.^HCl^45^ (10.2 g, 38.9 mmol) in CH_2_Cl_2_ (500 mL) under a N_2_ atmosphere was added *N*,*N*-diisopropylethylamine (27.1 mL, 157 mmol) in one portion. The resulting clear yellow solution was cooled to 0 °C using an ice-water bath, and 1-hydroxybenzotriazole hydrate (5.26 g, 38.9 mmol) was added in one portion, followed by portionwise addition of 1-[bis(dimethylamino)methylene]-1*H*-1,2,3-triazolo[4,5-*b*]pyridinium 3-oxid hexafluorophosphate (14.8 g, 38.9 mmol). After 21 h, the resulting reaction mixture was washed with HCl (2 × 200 mL, aq, 1 M), NaHCO_3_ (2 × 200 mL, sat. aq), and brine (200 mL). The organic phase was then dried over Na_2_SO_4_, filtered, and concentrated under reduced pressure. The resulting crude residue was used without further purification in the next reaction. Analytically pure dipeptide **13** was obtained by purification by column chromatography (50–100% EtOAc/*n*-heptane). Yield: 14.4 g (crude). Purified **13** was isolated as faint yellow crystals. >95% pure by NMR and a single spot by TLC. *R*_*f*_: 0.33 in 75% EtOAc/*n*-heptane. Optical rotation: [α]_D_^20^: −54.1 (*c* = 1.06, CH_2_Cl_2_). ^1^H NMR (CDCl_3_, 400 MHz): δ 8.19–8.13 (m, 2H), 7.36–7.30 (m, 2H), 7.24–7.15 (m, 1H), 5.51 (br, d, *J* = 7.6 Hz, 1H), 4.94–4.85 (m, 1H), 4.20–4.00 (m, 2H), 3.74 (s, 3H), 3.68–3.58 (m, 1H), 3.30 (dd, *J* = 14.0, 5.6 Hz, 1H), 3.17 (dd, *J* = 14.0, 6.8 Hz, 1H), 2.58 (dd, *J* = 8.8, 4.8 Hz, 1H), 1.42 (s, 9H) ppm. ^13^C NMR (CDCl_3_, 101 MHz): δ 171.5, 171.2, 156.3, 147.4, 143.7, 130.3, 123.9, 81.0, 62.8, 55.2, 53.0, 52.9, 37.7, 28.4 ppm. FTIR (neat): 3535 (br), 3486 (br), 3357 (br), 3258 (br), 1733 (m), 1682 (m), 1664 (s), 1542 (m), 1524 (s), 1346 (s), 1279 (m), 1245 (m), 1161 (s), 1051 (m), 859 (m), 756 (m), 687 (m), 518 (m) cm^–1^. HRMS-ESI (*m*/*z*): [M + H]^+^ calcd for C_18_H_25_N_3_O_8_, 412.1720; found, 412.1716. mp 92–94 °C (obtained by crystallization from EtOAc/*n*-heptane).

### Methyl (*R*)-2-((*S*)-2-((*tert*-Butoxycarbonyl)amino)-3-((*tert*-butyldiphenylsilyl)oxy)propanamido)-3-(4-nitrophenyl)propanoate (**16**)

To a stirred solution of crude dipeptide **13** (14.4 g) in CH_2_Cl_2_ (250 mL) at 0 °C under a N_2_ atmosphere was added imidazole (2.86 g, 42.0 mmol) in one portion. *tert*-Butyl(chloro)diphenylsilane (10.0 mL, 38.5 mmol) was then added dropwise over 5 min, after which 4-(dimethylamino)pyridine (429 mg, 3.50 mmol) was added in one portion. The reaction mixture turned white and cloudy. After 45 min, the cooling bath was removed, and after a further 45 min, the resulting reaction mixture was washed with HCl (3 × 100 mL, aq, 1 M) and brine (100 mL). The organic phase was then dried over Na_2_SO_4_, filtered, and concentrated under reduced pressure. The resulting crude residue was used in the next reaction without further purification. Analytically pure silyl ether **16** was obtained by purification by column chromatography (33–75% EtOAc/*n*-heptane). Yield: 26.6 g (crude). Purified **16** was isolated as a white amorphous solid. >95% pure by NMR and a single spot by TLC. *R*_*f*_: 0.25 in 33% EtOAc/*n*-heptane. Optical rotation: [α]_D_^20^: −20.8 (*c* = 0.53, CH_2_Cl_2_). ^1^H NMR (CDCl_3_, 400 MHz): δ 8.13–8.05 (m, 2H), 7.67–7.57 (m, 4H), 7.50–7.36 (m, 6H), 7.31–7.23 (m, 2H), 7.01 (br, d, *J* = 8.0 Hz, 1H), 5.20 (br, d, *J* = 7.6 Hz, 1H), 4.94 (dt, *J* = 7.6, 6.0 Hz, 1H), 4.32–4.20 (m, 1H), 4.12 (dd, *J* = 10.4, 4.4 Hz, 1H), 3.79 (dd, *J* = 10.4, 4.8 Hz, 1H), 3.74 (s, 3H), 3.29 (dd, *J* = 13.6, 6.0 Hz, 1H), 3.17 (dd, *J* = 13.6, 6.0 Hz, 1H), 1.45 (s, 9H), 1.05 (s, 9H) ppm. ^13^C NMR (CDCl_3_, 101 MHz): δ 171.1, 170.2, 155.7, 147.3, 143.6, 135.7, 135.6, 132.9, 132.5, 130.4, 130.21, 130.19, 128.1, 128.0, 123.8, 80.7, 64.1, 56.4, 53.0, 52.7, 38.0, 28.4, 26.9, 19.4 ppm. FTIR (neat): 3339 (w), 3322 (w), 2952 (w), 2855 (w), 1735 (m), 1652 (m), 1515 (s), 1344 (m), 1109 (m), 700 (m), 613 (m), 508 (m), 487 (m) cm^–1^. HRMS-ESI (*m*/*z*): [M + H]^+^ calcd for C_34_H_43_N_3_O_8_Si, 650.2898; found, 650.2897. mp 130–131 °C (obtained by concentration from EtOAc/*n*-heptane).

### Methyl (*R*)-1-[*N*-(*tert*-Butoxycarbonyl)-*O*-(*tert*-butyldiphenylsilyl)-l-seryl]-6-nitroindoline-2-carboxylate (**12**)

A stirred suspension of crude silyl ether **16** (26.5 g), PhI(OAc)_2_ (22.5 g, 70.0 mmol), and Pd(OAc)_2_ (393 mg, 1.75 mmol) in toluene (250 mL) was heated to reflux. After 16 h, the resulting reaction mixture was cooled to room temperature and concentrated under reduced pressure. The resulting residue was taken up in CH_2_Cl_2_, filtered through a plug of silica, and concentrated under reduced pressure. The resulting crude residue was used in the next reaction without further purification. Analytically pure indoline **12** was obtained by purification by column chromatography (25% EtOAc/*n*-heptane). Yield: 37.8 g (crude). Purified **12** was isolated as yellow oil. >95% pure by NMR and a single spot by TLC. *R*_*f*_: 0.27 in 33% EtOAc/*n*-heptane. Optical rotation: [α]_D_^20^: +27.4 (*c* = 0.50, CH_2_Cl_2_). ^1^H NMR (CDCl_3_, 400 MHz): δ 8.93 (br, s, 1H), 7.95 (dd, *J* = 8.0, 2.0 Hz, 1H), 7.75–7.56 (m, 4H), 7.54–7.19 (m, 7H), 5.79 (br, d, *J* = 8.8 Hz, 1H), 5.09 (br, d, *J* = 8.8 Hz, 1H), 4.59–4.45 (m, 1H), 4.06 (dd, *J* = 10.4, 4.8 Hz, 1H), 3.88 (dd, *J* = 10.4, 8.4 Hz, 1H), 3.80–3.52 (m, 4H), 3.41 (br, d, *J* = 16.0 Hz, 1H), 1.43 (s, 9H), 1.08 (s, 9H) ppm. ^13^C NMR (CDCl_3_, 101 MHz): δ 171.3, 170.2, 155.9, 148.2, 143.2, 137.0, 135.74, 135.70, 133.2, 133.1, 130.1, 130.0, 128.0, 127.9, 124.6, 120.2, 113.0, 80.4, 63.6, 61.1, 54.8, 53.2, 33.3, 28.4, 26.9, 19.4 ppm. FTIR (film): 3342 (br), 2955 (w), 2931 (w), 2858 (w), 1748 (m), 1704 (m), 1668 (m), 1525 (m), 1481 (m), 1428 (m), 1344 (m), 1161 (m), 1111 (s), 1007 (m), 909 (m), 819 (m), 732 (m), 701 (s), 613 (m), 503 (m), 486 (m) cm^–1^. HRMS-ESI (*m*/*z*): [M + H]^+^ calcd for C_34_H_41_N_3_O_8_Si, 648.2741; found, 648.2753.

### (3*S*,10*aR*)-3-{[(*tert*-Butyldiphenylsilyl)oxy]methyl}-7-nitro-2,3,10,10*a*-tetrahydropyrazino[1,2-*a*]indole-1,4-dione (**17**)

To a stirred solution of crude indoline **12** (37.8 g) in CH_2_Cl_2_ (250 mL) at 0 °C under a N_2_ atmosphere was added TFA (10 mL) dropwise over 5 min. Additional TFA (20 mL) was then added in one portion. After 30 min, the cooling bath was removed, and after a further 115 min, TFA (10 mL) was added in one portion. After a further 1 h, water (200 mL) was added, and the resulting mixture was neutralized with NaHCO_3_ (s). The organic phase was separated, and the aqueous phase was extracted with CH_2_Cl_2_ (3 × 100 mL). The combined organic extracts were concentrated under reduced pressure. The resulting crude residue was purified by column chromatography (50–75% EtOAc/*n*-heptane) to give diketopiperazine **17**. Yield: 9.37 g (49% over four steps from **14**). Isolated as a beige amorphous solid. >95% pure by NMR and a single spot by TLC. *R*_*f*_: 0.19 in 50% EtOAc/*n*-heptane. Optical rotation: [α]_D_^20^: −8.5 (*c* = 0.53, CH_2_Cl_2_). ^1^H NMR (CDCl_3_, 400 MHz): δ 8.94 (d, *J* = 2.2 Hz, 1H), 8.04 (dd, *J* = 8.2, 2.2 Hz, 1H), 7.62–7.54 (m, 4H), 7.46–7.30 (m, 7H), 6.39 (d, *J* = 4.0 Hz, 1H), 5.09 (app. t, *J* = 10.8, 10.0 Hz, 1H), 4.26 (dd, *J* = 10.4, 4.0 Hz, 1H), 4.21–4.16 (m, 1H), 3.93 (dd, *J* = 10.4, 2.8 Hz, 1H), 3.49 (ddd, *J* = 17.4, 10.8, 1.6 Hz, 1H), 3.37 (dd, *J* = 17.4, 10.0 Hz, 1H), 1.03 (s, 9H) ppm. ^13^C NMR (CDCl_3_, 101 MHz): δ 168.5, 163.8, 148.2, 142.2, 136.8, 135.57, 135.56, 132.3, 132.1, 130.4, 128.20, 128.16, 125.3, 120.9, 111.5, 66.7, 60.6, 60.3, 31.8, 27.0, 19.3 ppm. FTIR (film): 3242 (br), 2931 (w), 2858 (w), 1684 (s), 1601 (w), 1526 (m), 1479 (m), 1429 (m), 1343 (m), 1107 (m), 738 (m), 702 (m), 505 (w) cm^–1^. HRMS-ESI (*m*/*z*): [M + NH_4_]^+^ calcd for C_28_H_29_N_3_O_5_Si, 533.2220; found, 533.2219. mp 209–210 °C (obtained by concentration from EtOAc/*n*-heptane).

### (3*S*,10*aR*)-3-{[(*tert*-Butyldiphenylsilyl)oxy]methyl}-2-methyl-7-nitro-2,3,10,10*a*-tetrahydropyrazino[1,2-*a*]indole-1,4-dione (**30**)

To a stirred suspension of diketopiperazine **17** (5.87 g, 11.4 mmol) and K_2_CO_3_ (39.3 g, 284 mmol) in acetone (100 mL) was added iodomethane (60.0 mL, 964 mmol) in one portion. The flask was wrapped in aluminum foil. After 7 d, the resulting reaction mixture was filtered and concentrated under reduced pressure. Safety note: Iodomethane is toxic. Handling of this reagent and decontamination of equipment require appropriate protocols to avoid exposure. The resulting crude residue was purified by column chromatography (33–75% EtOAc/*n*-heptane) to give *N*-methyl diketopiperazine **30**. Yield: 5.35 g (89%). Isolated as a beige amorphous solid. >95% pure by NMR and a single spot by TLC. *R*_*f*_: 0.40 in 50% EtOAc/*n*-heptane. Optical rotation: [α]_D_^20^: −2.4 (*c* = 0.21, CH_2_Cl_2_). ^1^H NMR (CDCl_3_, 400 MHz): δ 8.93 (d, *J* = 2.2 Hz, 1H), 8.04 (dd, *J* = 8.2, 2.2 Hz, 1H), 7.61–7.52 (m, 4H), 7.46–7.30 (m, 7H), 5.15 (app. t, *J* = 10.8, 10.0 Hz, 1H), 4.26 (dd, *J* = 11.8, 3.6 Hz, 1H), 4.04 (dd, *J* = 11.8, 2.8 Hz, 1H), 4.04–4.00 (m, 1H), 3.50 (ddd, *J* = 17.4, 10.8, 1.2 Hz, 1H), 3.39 (dd, *J* = 17.4, 10.0 Hz, 1H), 2.99 (s, 3H), 1.01 (s, 9H) ppm. ^13^C NMR (CDCl_3_, 101 MHz): δ 166.8, 163.8, 148.2, 142.0, 137.5, 135.6, 135.5, 132.3, 132.0, 130.4, 128.23, 128.17, 125.3, 120.9, 111.5, 67.3, 63.6, 60.5, 32.7, 32.2, 27.0, 19.3 ppm. FTIR (film): 2931 (w), 2858 (w), 1677 (s), 1527 (m), 1483 (m), 1430 (m), 1346 (m), 1110 (m), 740 (m), 704 (m) cm^–1^. HRMS-ESI (*m*/*z*): [M + NH_4_]^+^ calcd for C_29_H_31_N_3_O_5_Si, 547.2377; found, 547.2371. mp 154–156 °C (obtained by concentration from EtOAc/*n*-heptane).

### (3*S*,10*aR*)-3-(Hydroxymethyl)-2-methyl-7-nitro-2,3,10,10*a*-tetrahydropyrazino[1,2-*a*]indole-1,4-dione (**11**)

To a stirred solution of *N*-methyl diketopiperazine **30** (670 mg, 1.26 mmol) in pyridine (6 mL) was added hydrofluoric acid (2 mL, 48% w/w) in one portion. After 1 h, the resulting reaction mixture was neutralized with NaHCO_3_ (s). The resulting mixture was diluted with water and extracted with EtOAc (2 × 20 mL). The combined organic extracts were washed with HCl (20 mL, aq, 1 M) and brine (50 mL). The organic phase was then dried over Na_2_SO_4_, filtered, and concentrated under reduced pressure. The resulting crude residue was purified by column chromatography (EtOAc) to give alcohol **11**. Yield: 365 mg (>95%). Isolated as a yellow amorphous solid. >95% pure by NMR and a single spot by TLC. *R*_*f*_: 0.19 in EtOAc. Optical rotation: [α]_D_^20^: +79.4 (*c* = 0.64, DMSO). ^1^H NMR (DMSO-*d*_6_, 400 MHz): δ 8.65 (d, *J* = 2.2 Hz, 1H), 8.00 (dd, *J* = 8.2, 2.2 Hz, 1H), 7.57 (d, *J* = 8.2 Hz, 1H), 5.54 (app. t, *J* = 5.6, 5.2 Hz, 1H), 5.20 (app. t, *J* = 10.8, 10.0 Hz, 1H), 4.17 (app. t, *J* = 2.8, 2.4 Hz, 1H), 3.92 (ddd, *J* = 11.7, 5.2, 2.4 Hz, 1H), 3.88 (ddd, *J* = 11.7, 5.6, 2.8 Hz, 1H), 3.46 (dd, *J* = 17.6, 10.0 Hz, 1H), 3.32 (ddd, *J* = 17.6, 10.8, 1.2 Hz, 1H), 2.96 (s, 3H) ppm. ^13^C NMR (DMSO-*d*_6_, 101 MHz): δ 166.4, 165.1, 146.9, 141.9, 139.0, 125.9, 120.2, 109.3, 66.6, 61.0, 59.9, 31.7, 31.6 ppm. FTIR (neat): 3368 (br), 1680 (m), 1650 (m), 1519 (m), 1482 (m), 1402 (m), 1331 (m), 1074 (m), 1060 (m), 892 (m), 815 (m), 742 (m), 522 (m) cm^–1^. HRMS-ESI (*m*/*z*): [M + H]^+^ calcd for C_13_H_13_N_3_O_5_, 292.0933; found, 292.0934. mp 224–225 °C (obtained by concentration from EtOAc).

### [(3*S*,10*aR*)-2-Methyl-7-nitro-1,4-dioxo-1,2,3,4,10,10*a*-hexahydropyrazino(1,2-*a*)indol-3-yl]methyl Acetate (**18**)

A stirred suspension of alcohol **11** (291 mg, 1.00 mmol) in acetic anhydride (3.0 mL) was heated with microwave irradiation to 100 °C. After 2 h, the resulting reaction mixture was cooled to room temperature and diluted with EtOAc (25 mL). The resulting solution was washed with NaHCO_3_ (3 × 10 mL) and brine (10 mL). The organic phase was then dried over Na_2_SO_4_, filtered, and concentrated under reduced pressure. The resulting crude residue was purified by column chromatography (50–75% EtOAc/*n*-heptane) to give acetate **18**. Yield: 313 mg (94%). Isolated as a yellow amorphous solid. >95% pure by NMR and a single spot by TLC. *R*_*f*_: 0.22 in 75% EtOAc/*n*-heptane. Optical rotation: [α]_D_^20^: +103 (*c* = 0.1, CHCl_3_). ^1^H NMR (CDCl_3_, 400 MHz): δ 8.87 (d, *J* = 2.4 Hz, 1H), 8.03 (dd, *J* = 8.4, 2.4 Hz, 1H), 7.41 (d, *J* = 8.4 Hz, 1H), 5.03 (app. t, *J* = 10.8, 10.0 Hz, 1H), 4.68 (dd, *J* = 12.0, 4.0 Hz, 1H), 4.48 (dd, *J* = 12.0, 3.6 Hz, 1H), 4.25 (app. t, *J* = 4.0, 3.6 Hz, 1H), 3.58 (ddd, *J* = 17.2, 10.8, 1.6 Hz, 1H), 3.52 (dd, *J* = 17.2, 10.0 Hz, 1H), 3.10 (s, 3H), 2.08 (s, 3H) ppm. ^13^C NMR (CDCl_3_, 101 MHz): δ 170.0, 166.4, 162.6, 148.2, 141.9, 137.2, 125.3, 121.1, 111.7, 64.3, 62.6, 60.1, 32.9, 32.4, 20.9 ppm. FTIR (film): 2938 (w), 1746 (m), 1675 (s), 1601 (w), 1525 (m), 1484 (m), 1435 (m), 1403 (m), 1346 (m), 1226 (m), 1076 (w), 1058 (w), 1035 (w), 892 (w), 831 (w), 739 (w) cm^–1^. HRMS-ESI (*m*/*z*): [M + H]^+^ calcd for C_15_H_15_N_3_O_6_, 334.1039; found, 334.1036. mp 164–166 °C (obtained by concentration from EtOAc/*n*-heptane).
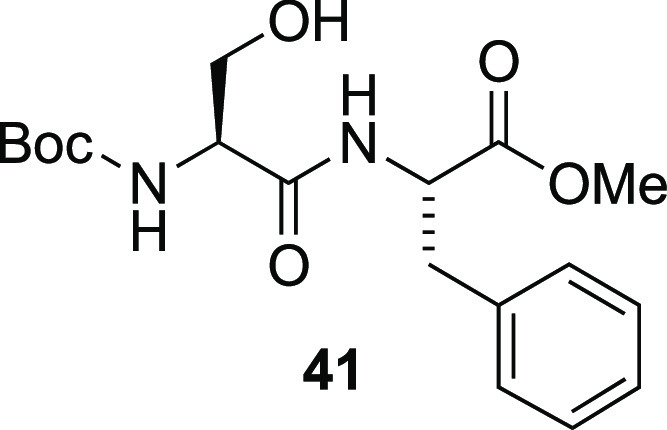


### Methyl (*tert*-Butoxycarbonyl)-l-seryl-l-phenylalaninate (**41**)^46^

To a stirred solution of serine **14** (2.05 g, 10.0 mmol) and l-phenylalanine methyl ester hydrochloride (2.37 g, 11.0 mmol) in CH_2_Cl_2_ (100 mL) under a N_2_ atmosphere was added *N*,*N*-diisopropylethylamine (6.97 mL, 40.0 mmol) in one portion. The resulting clear colorless solution was cooled to 0 °C with an ice-water bath, and 1-hydroxybenzotriazole hydrate (1.62 g, 12.0 mmol) was added in one portion, followed by portionwise addition of 1-[bis(dimethylamino)methylene]-1*H*-1,2,3-triazolo[4,5-*b*]pyridinium 3-oxid hexafluorophosphate (4.56 g, 12.0 mmol). The reaction mixture turned bright yellow. After 19 h, the resulting reaction mixture was washed with NaHCO_3_ (2 × 40 mL, sat. aq), HCl (2 × 40 mL, aq, 1 M), and brine (40 mL). The organic phase was then dried over Na_2_SO_4_, filtered, and concentrated under reduced pressure. The resulting crude residue was used in the next reaction without further purification. Analytically pure dipeptide **41** was obtained by purification by column chromatography (50–75% EtOAc/*n*-heptane). Yield: 9.1 g (crude). Purified **41** was isolated as colorless oil that partially solidified upon standing. >95% pure by NMR and a single spot by TLC. ^1^H NMR (CDCl_3_, 400 MHz): δ 7.35–7.17 (m, 3H), 7.17–7.08 (m, 2H), 7.07–6.90 (m, 1H), 5.55–5.39 (m, 1H), 4.91–4.76 (m, 1H), 4.16 (br, s, 1H), 4.00 (br, d, *J* = 11.2 Hz, 1H), 3.73 (s, 3H), 3.60 (dd, *J* = 11.2, 5.6 Hz, 1H), 3.18 (dd, *J* = 14.0, 5.6 Hz, 1H), 3.05 (dd, *J* = 14.0, 6.8 Hz, 1H), 2.77 (br, s, 1H), 1.44 (s, 9H) ppm. ^13^C NMR (CDCl_3_, 101 MHz): δ 171.9, 171.2, 156.0, 135.8, 129.3, 128.8, 127.3, 80.6, 63.0, 55.1, 53.5, 52.6, 37.8, 28.4 ppm. HRMS-ESI (*m*/*z*): [M + H]^+^ calcd for C_18_H_26_N_2_O_6_, 367.1869; found, 367.1867.

### Methyl *N*-(*tert*-Butoxycarbonyl)-*O*-(*tert*-butyldiphenylsilyl)-l-seryl-l-phenylalaninate (**19**)

To a stirred solution of crude dipeptide **41** (9.1 g) in CH_2_Cl_2_ (100 mL) under a N_2_ atmosphere were added imidazole (953 mg, 14.0 mmol) and 4-(dimethylamino)pyridine (122 mg, 1.00 mmol) in one portion. The reaction mixture was cooled to 0 °C with an ice-water bath, and *tert*-butyl(chloro)diphenylsilane (3.11 mL, 12.0 mmol) was added dropwise over 2 min. After 105 min, the resulting reaction mixture was washed with HCl (3 × 50 mL, aq, 1 M) and brine (50 mL). The organic phase was then dried over Na_2_SO_4_, filtered, and concentrated under reduced pressure. The resulting crude residue was purified by column chromatography (12.5–50% EtOAc/*n*-heptane) to give silyl ether **19**. Yield: 5.40 g (89% over two steps from **14**). Isolated as a white amorphous solid. >95% pure by NMR and a single spot by TLC. *R*_*f*_: 0.44 in 33% EtOAc/*n*-heptane. Optical rotation: [α]_D_^20^: +24.2 (*c* = 0.53, CH_2_Cl_2_). ^1^H NMR (CDCl_3_, 400 MHz): δ 7.72–7.57 (m, 4H), 7.48–7.33 (m, 6H), 7.31–7.17 (m, 3H), 7.16–7.03 (m, 2H), 6.91 (br, d, *J* = 7.2 Hz, 1H), 5.19 (br, s, 1H), 4.93–4.83 (m, 1H), 4.25 (br, s, 1H), 4.01 (dd, *J* = 10.0, 4.4 Hz, 1H), 3.77 (dd, *J* = 10.0, 5.8 Hz, 1H), 3.65 (s, 3H), 3.20–3.05 (m, 2H), 1.42 (s, 9H), 1.04 (s, 9H) ppm. ^13^C NMR (CDCl_3_, 101 MHz): δ 171.6, 170.1, 155.6, 135.9, 135.7, 135.6, 133.0, 132.6, 130.0, 130.0, 129.4, 128.7, 128.00, 127.98, 127.3, 80.3, 64.1, 55.9, 53.6, 52.4, 38.3, 28.4, 26.9, 19.4 ppm. FTIR (film): 3342 (br), 3070 (w), 2931 (w), 2858 (w), 1745 (m), 1677 (s), 1496 (m), 1366 (m), 1168 (m), 1112 (s), 742 (w), 702 (s), 505 (m) cm^–1^. HRMS-ESI (*m*/*z*): [M + H]^+^ calcd for C_34_H_44_N_2_O_6_Si, 605.3056; found, 605.3060. mp 116–117 °C (obtained by concentration from EtOAc/*n*-heptane).
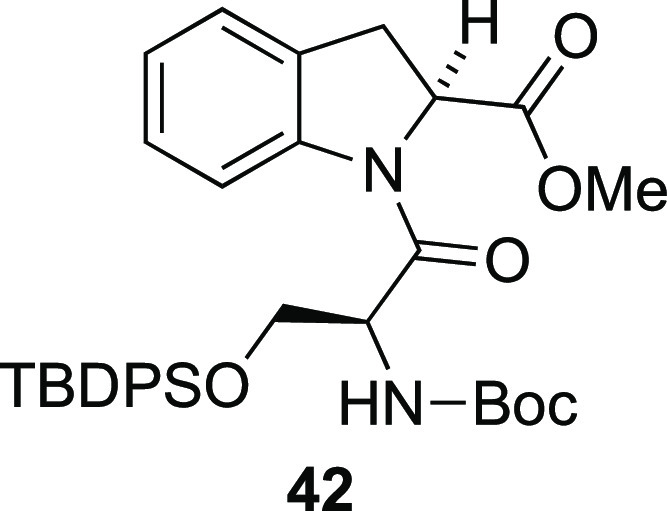


### Methyl (*S*)-1-[*N*-(*tert*-Butoxycarbonyl)-*O*-(*tert*-butyldiphenylsilyl)-l-seryl]indoline-2-carboxylate (**42**)

A stirred suspension of silyl ether **19** (5.00 g, 8.27 mmol), PhI(OAc)_2_ (5.33 g, 16.5 mmol), and Pd(OAc)_2_ (92.8 mg, 413 μmol) in toluene (83 mL) was heated to reflux. After 24 h, the resulting reaction mixture was cooled to room temperature and concentrated under reduced pressure. The resulting crude residue was purified by column chromatography (12.5% EtOAc/*n*-heptane) to give indoline **42**. Yield: 2.16 g (43%). Isolated as golden oil/foam. >95% pure by NMR and a single spot by TLC. *R*_*f*_: 0.29 in 25% EtOAc/*n*-heptane. Optical rotation: [α]_D_^20^: −50.6 (*c* = 0.69, CHCl_3_). ^1^H NMR (CDCl_3_, 400 MHz): δ 8.33 (d, *J* = 8.0 Hz, 1H), 7.61–7.51 (m, 4H), 7.47–7.34 (m, 3H), 7.33–7.23 (m, 4H), 7.18–7.12 (m, 1H), 7.12–7.04 (m, 1H), 5.39 (d, *J* = 9.2 Hz, 1H), 5.28 (dd, *J* = 9.2, 4.0 Hz, 1H), 4.75 (td, *J* = 9.2, 5.6 Hz, 1H), 3.88 (dd, *J* = 9.2, 5.6 Hz, 1H), 3.83–3.73 (m, 4H), 3.38–3.21 (m, 2H), 1.43 (s, 9H), 0.99 (s, 9H) ppm. ^13^C NMR (CDCl_3_, 101 MHz): δ 171.8, 170.6, 154.8, 142.2, 135.6, 135.5, 132.7, 132.7, 130.0, 129.2, 128.0, 127.9, 124.7, 124.5, 117.6, 79.8, 66.1, 60.6, 53.8, 53.3, 33.2, 28.4, 26.9, 19.3 ppm. FTIR (film): 3433 (w), 2955 (w), 2931 (w), 2858 (w), 1743 (m), 1712 (m), 1658 (m), 1480 (m), 1414 (m), 1366 (m), 1163 (m), 1106 (m), 1014 (m), 909 (m), 823 (w), 756 (m), 731 (s), 701 (s), 613 (m), 504 (m), 489 (w) cm^–1^. HRMS-ESI (*m*/*z*): [M + H]^+^ calcd for C_34_H_42_N_2_O_6_Si, 603.2890; found, 603.2894.

### (3*S*,10*aS*)-3-{[(*tert*-Butyldiphenylsilyl)oxy]methyl}-2,3,10,10*a*-tetrahydropyrazino[1,2-*a*]indole-1,4-dione (**20**)

To a stirred solution of indoline **42** (2.16 g, 3.58 mmol) in CH_2_Cl_2_ (30 mL) at 0 °C under a N_2_ atmosphere was added TFA (10 mL) dropwise over 5 min. After 50 min, the resulting reaction mixture was poured out onto water (50 mL) and neutralized with NaHCO_3_ (s). The organic phase was separated, and the aqueous phase was extracted with CH_2_Cl_2_ (2 × 50 mL). The combined organic extracts were dried over Na_2_SO_4_, filtered, and concentrated under reduced pressure. The resulting crude residue was purified by column chromatography (40% EtOAc/*n*-heptane) to give diketopiperazine **20**. Yield: 1.22 g (72%). Isolated as yellow foam. >95% pure by NMR and a single spot by TLC. *R*_*f*_: 0.40 in 50% EtOAc/*n*-heptane. Optical rotation: [α]_D_^20^: −26.4 (*c* = 0.72, CH_2_Cl_2_). ^1^H NMR (CDCl_3_, 400 MHz): δ 8.00 (d, *J* = 7.6 Hz, 1H), 7.70–7.61 (m, 4H), 7.50–7.36 (m, 6H), 7.29–7.18 (m, 2H), 7.10 (dt, *J* = 7.6, 1.2 Hz, 1H), 6.43 (br, s, 1H), 4.80 (t, *J* = 10.0 Hz, 1H), 4.37–4.29 (m, 2H), 3.93 (dd, *J* = 12.0, 11.2 Hz, 1H), 3.58 (dd, *J* = 16.8, 10.0 Hz, 1H), 3.40 (dd, *J* = 16.8, 10.0 Hz, 1H), 1.10 (s, 9H) ppm. ^13^C NMR (CDCl_3_, 101 MHz): δ 168.5, 162.8, 140.9, 135.7, 135.6, 132.6, 132.4, 130.34, 130.31, 129.6, 128.2, 128.0, 125.3, 125.1, 116.2, 63.0, 60.1, 56.4, 31.1, 27.0, 19.3 ppm. FTIR (film): 3214 (br), 3071 (w), 2956 (w), 2857 (w), 1680 (s), 1486 (m), 1411 (m), 1112 (m), 739 (m), 702 (m), 505 (m) cm^–1^. HRMS-ESI (*m*/*z*): [M + H]^+^ calcd for C_28_H_30_N_2_O_3_Si, 471.2104; found, 471.2103. mp 77–80 °C (obtained by concentration from EtOAc/*n*-heptane).

### (3*S*,10*aS*)-3-{[(*tert*-Butyldiphenylsilyl)oxy]methyl}-2-methyl-2,3,10,10*a*-tetrahydropyrazino[1,2-*a*]indole-1,4-dione (**35**)

To a stirred suspension of diketopiperazine **20** (1.00 g, 2.12 mmol) and K_2_CO_3_ (7.31 g, 53.1 mmol) in acetone (30 mL) was added iodomethane (20 mL, 321 mmol) in one portion. The flask was wrapped in aluminum foil. After 5 d, the reaction mixture was heated to reflux. After a further 17 h, the resulting reaction mixture was cooled to room temperature and concentrated under reduced pressure. Safety note: iodomethane is toxic. Handling of this reagent and decontamination of equipment require appropriate protocols to avoid exposure. The resulting residue was mixed with water (100 mL) and extracted with CH_2_Cl_2_ (3 × 50 mL). The combined organic extracts were dried over Na_2_SO_4_, filtered, and concentrated under reduced pressure. The resulting crude residue was purified by column chromatography (50% EtOAc/*n*-heptane) to give *N*-methyl diketopiperazine **35**. Yield: 852 mg (83%). Isolated as a white amorphous solid. >95% pure by NMR and a single spot by TLC. *R*_*f*_: 0.24 in 50% EtOAc/*n*-heptane. Optical rotation: [α]_D_^20^: −48.4 (*c* = 0.61, CH_2_Cl_2_). ^1^H NMR (CDCl_3_, 400 MHz): δ 8.12–8.06 (m, 1H), 7.60–7.51 (m, 4H), 7.45–7.36 (m, 2H), 7.36–7.23 (m, 6H), 7.14 (dt, *J* = 7.6, 1.2 Hz, 1H), 4.80 (t, *J* = 10.0 Hz, 1H), 4.40–4.34 (m, 1H), 4.13–4.09 (m, 1H), 4.09 (dd, *J* = 15.2, 2.4 Hz, 1H), 3.41 (d, *J* = 10.0 Hz, 2H), 3.02 (s, 3H), 0.92 (s, 9H) ppm. ^13^C NMR (CDCl_3_, 101 MHz): δ 167.0, 162.3, 141.8, 135.8, 135.7, 133.0, 132.6, 130.2, 130.1, 130.0, 127.9, 127.94, 127.92, 125.3, 125.0, 117.0, 64.3, 62.0, 60.1, 32.8, 31.2, 26.8, 19.3 ppm. FTIR (film): 3071 (w), 3050 (w), 2857 (w), 1670 (s), 1485 (m), 1428 (m), 1113 (m), 756 (m), 736 (m), 703 (m), 505 (m) cm^–1^. HRMS-ESI (*m*/*z*): [M + H]^+^ calcd for C_29_H_32_N_2_O_3_Si, 485.2260; found, 485.2260. mp 142–143 °C (obtained by concentration from EtOAc/*n*-heptane).

### (3*S*,10*aS*)-3-(Hydroxymethyl)-2-methyl-2,3,10,10*a*-tetrahydropyrazino[1,2-*a*]indole-1,4-dione (**21**)^47^

A solution of diketopiperazine **35** (362 mg, 747 μmol) in pyridine (4 mL) was added dropwise to stirred hydrofluoric acid (2 mL, 48% w/w) over 12 min. A white suspension formed immediately. Pyridine (4 mL) was added, and a clear, colorless solution formed. After 17 h, the resulting reaction mixture was neutralized with NaHCO_3_ (s) and then diluted with water (25 mL) and NaHCO_3_ (25 mL, sat. aq). The aqueous mixture was extracted with EtOAc (4 × 25 mL). The combined organic extracts were washed with HCl (25 mL, aq, 1 M) and brine (25 mL). The organic phase was then dried over Na_2_SO_4_, filtered, and concentrated under reduced pressure. The resulting crude residue was purified by column chromatography (EtOAc) to give alcohol **21**. Yield: 135 mg (73%). Isolated as a white crystalline solid. >95% pure by NMR and a single spot by TLC. *R*_*f*_: 0.21 in EtOAc. Optical rotation: [α]_D_^20^: −45.0 (*c* = 0.10, CHCl_3_). ^1^H NMR (CDCl_3_, 400 MHz): δ 8.04 (d, *J* = 8.0 Hz, 1H), 7.31–7.22 (m, 2H), 7.13 (dt, *J* = 7.6, 1.2 Hz, 1H), 4.75 (app. t, *J* = 10.0, 9.6 Hz, 1H), 4.52–4.42 (m, 1H), 4.18–4.07 (m, 2H), 3.64 (dd, *J* = 16.8, 9.6 Hz 1H), 3.43 (dd, *J* = 16.8, 10.0 Hz, 1H), 3.18 (s, 3H), 3.04–2.96 (m, 1H) ppm. ^13^C NMR (CDCl_3_, 101 MHz): δ 168.5, 163.8, 140.7, 130.1, 128.0, 125.6, 125.2, 116.4, 61.4, 59.9, 59.3, 31.7, 29.8 ppm. FTIR (neat): 3362 (br), 1659 (m), 1642 (m), 1600 (m), 1484 (m), 1415 (m), 1394 (m), 1238 (m), 1064 (m), 1010 (m), 754 (s), 409 (m) cm^–1^. HRMS-ESI (*m*/*z*): [M + H]^+^ calcd for C_13_H_14_N_2_O_3_, 247.1083; found, 247.1082. mp 130–133 °C (obtained by concentration from EtOAc/*n*-heptane).

### Methyl (*R*)-2-{2-[(*tert*-Butoxycarbonyl)amino]acetamido}-3-(4-nitrophenyl)propanoate (ent-**9**)^26^

To a stirred suspension of glycine **22** (7.71 g, 44.0 mmol) and nitrophenyl alanine **15**^.^HCl^45^ (10.4 g, 40 mmol) in CH_2_Cl_2_ (500 mL) under a N_2_ atmosphere was added triethylamine (22.3 mL, 160 mmol) in one portion. The resulting clear yellow solution was cooled to 0 °C with an ice-water bath, and 1-[bis(dimethylamino)methylene]-1*H*-1,2,3-triazolo[4,5-*b*]pyridinium 3-oxid hexafluorophosphate (16.7 g, 44.0 mmol) was added portionwise, followed by addition of 1-hydroxybenzotriazole hydrate (5.95 g, 44 mmol) in one portion. After 30 min, the cooling bath was removed. After a further 15 h, the resulting reaction mixture was washed with HCl (2 × 200 mL, aq, 1 M), NaHCO_3_ (2 × 200 mL, sat. aq), and brine (100 mL). The organic phase was then dried over Na_2_SO_4_, filtered, and concentrated under reduced pressure. The resulting crude residue was purified by column chromatography (50–75% EtOAc/*n*-heptane) to give dipeptide ent-**9**. Yield: 16.6 g (93%). Isolated as yellow oil that crystallized upon standing. >95% pure by NMR. *R*_*f*_: 0.17 in 50% EtOAc/*n*-heptane. Optical rotation: [α]_D_^20^: −53.2 (*c* = 0.62, CH_2_Cl_2_). ^1^H NMR (CDCl_3_, 400 MHz): δ 8.18–8.11 (m, 2H), 7.33–7.27 (m, 2H), 6.72 (br, d, *J* = 6.0 Hz, 1H), 5.08 (br, s, 1H), 4.92 (dt, *J* = 7.6, 6.0 Hz, 1H), 3.83 (dd, *J* = 17.0, 6.0 Hz, 1H), 3.73 (s, 3H), 3.72 (dd, *J* = 17.0, 6.0 Hz, 1H), 3.30 (dd, *J* = 13.8, 6.0 Hz, 1H), 3.17 (dd, *J* = 13.8, 6.0 Hz, 1H), 1.43 (s, 9H) ppm. ^13^C NMR (CDCl_3_, 101 MHz): δ 171.2, 169.5, 156.2, 147.3, 143.7, 130.4, 123.9, 80.8, 52.9, 52.8, 44.6, 37.9, 28.4 ppm. FTIR (film): 3342 (w), 3307 (w), 1738 (m), 1682 (m), 1667 (s), 1521 (s), 1347 (m), 1282 (m), 1162 (m), 855 (m), 700 (m), 539 (m) cm^–1^. HRMS-ESI (*m*/*z*): [M + Na]^+^ calcd for C_17_H_23_N_3_NaO_7_, 404.1434; found, 404.1430. mp 96–98 °C (obtained by concentration from EtOAc/*n*-heptane).

### Methyl (*R*)-1-[(*tert*-Butoxycarbonyl)glycyl]-6-nitroindoline-2-carboxylate (ent-**10**)^26^

A stirred suspension of dipeptide ent-**9** (11.6 g, 30.5 mmol), PhI(OAc)_2_ (19.6 g, 61.0 mmol), and Pd(OAc)_2_ (342 mg, 1.52 mmol) in toluene (300 mL) was heated to reflux. After 20 h, the resulting reaction mixture was cooled to room temperature and concentrated under reduced pressure. The resulting crude residue was used in the next step without further purification. Analytically pure indoline ent-**10** was obtained by purification by column chromatography (50% EtOAc/*n*-heptane). Yield: 9.11 g (crude). Purified ent-**10** was isolated as a faint yellow amorphous solid. >95% pure by NMR and a single spot by TLC. *R*_*f*_: 0.17 in 50% EtOAc/*n*-heptane. Optical rotation: [α]_D_^20^: +54.1 (*c* = 0.51 CH_2_Cl_2_) ^1^H NMR (CDCl_3_, 400 MHz): δ 8.99 (br, s, 1H), 7.95 (dd, *J* = 8.0, 2.0 Hz, 1H), 7.30 (d, *J* = 8.0 Hz, 1H), 5.44 (br, s, 1H), 5.10 (br, d, *J* = 9.2 Hz, 1H), 4.16 (br, d, *J* = 14.4 Hz, 1H), 3.93–3.56 (m, 5H), 3.41 (br, d, *J* = 17.2 Hz, 1H), 1.46 (s, 9H) ppm. ^13^C NMR (CDCl_3_, 101 MHz): δ 170.7, 168.1, 156.0, 148.4, 143.3, 135.7, 124.7, 120.2, 112.5, 80.3, 60.4, 53.6, 43.7, 33.7, 28.5 ppm. FTIR (film): 3399 (br), 2978 (w), 2929 (w), 1744 (m), 1709 (m), 1682 (s), 1524 (s), 1481 (m), 1345 (s), 1246 (m), 1667 (s), 740 (w) cm^–1^. HRMS-ESI (*m*/*z*): [M + H]^+^ calcd for C_17_H_21_N_3_O_7_, 380.1458; found, 380.1458. mp 111–113 °C (obtained by concentration from EtOAc/*n*-heptane).

### (*R*)-7-Nitro-2,3,10,10*a*-tetrahydropyrazino[1,2-*a*]indole-1,4-dione (**23**)

To a stirred solution of crude indoline ent-**10** (9.11 g) in CH_2_Cl_2_ (100 mL) at 0 °C under a N_2_ atmosphere was added TFA (20 mL) in one portion. After 15 min, the cooling bath was removed. After a further 45 min, the resulting reaction mixture was neutralized with NaHCO_3_ (s). The resulting mixture was diluted with NaHCO_3_ (100 mL, sat. aq) and then extracted with CH_2_Cl_2_ (2 × 100 mL). The combined organic extracts were washed with brine (100 mL). The organic phase was then dried over Na_2_SO_4_, filtered, and concentrated under reduced pressure. The resulting crude residue was purified by column chromatography (0–20% MeOH/EtOAc) to give a dark brown solid (4.6 g), which was triturated from boiling EtOAc/MeOH (10:7, 170 mL) to give diketopiperazine **23**. Yield: 1.84 g (24% over two steps from ent-**9**). Isolated as a beige amorphous solid. >95% pure by NMR. *R*_*f*_: 0.16 in EtOAc. Optical rotation: [α]_D_^20^: +90.0 (*c* = 0.05, acetone). ^1^H NMR (DMSO-*d*_6_, 400 MHz): δ 8.62 (d, *J* = 2.4 Hz, 1H), 8.45 (br, d, *J* = 5.0 Hz, 1H), 7.99 (dd, *J* = 8.4, 2.4 Hz, 1H), 7.58 (d, *J* = 8.4 Hz, 1H), 5.12 (t, *J* = 10.0 Hz, 1H), 4.29 (dd, *J* = 17.0, 0.4 Hz, 1H), 3.76 (dd, *J* = 17.0, 5.0 Hz, 1H), 3.45 (d, *J* = 10.0 Hz, 2H) ppm. ^13^C NMR (DMSO-*d*_6_, 101 MHz): δ 168.3, 164.7, 147.0, 141.7, 138.5, 126.0, 119.9, 108.7, 59.8, 46.4, 30.2 ppm. FTIR (neat): 3316 (br), 1675 (m), 1657 (m), 1598 (w), 1514 (m), 1474 (m), 1416 (m), 1340 (m), 1090 (m), 1074 (m), 889 (m), 709 (m) cm^–1^. HRMS-ESI (*m*/*z*): [M + NH_4_]^+^ calcd for C_11_H_9_N_3_O_4_, 265.0937; found, 265.0936. mp 237–239 °C (obtained by trituration from EtOAc/MeOH).

### (*R*)-2-Methyl-7-nitro-2,3,10,10*a*-tetrahydropyrazino[1,2-*a*]indole-1,4-dione (**24**)

To a stirred suspension of diketopiperazine **23** (1.79 g, 7.23 mmol) and K_2_CO_3_ (25.0 g, 181 mmol) in acetone (70 mL) was added iodomethane (40 mL, 642 mmol) in one portion. The flask was wrapped in aluminum foil. After 7 d, the resulting reaction mixture was concentrated under reduced pressure. Safety note: iodomethane is a toxic reagent. Handling of this reagent and decontamination of equipment require appropriate protocols to avoid exposure. The resulting residue was mixed with water (100 mL) and EtOAc (100 mL). The organic phase was separated, and the aqueous phase was extracted with EtOAc (3 × 50 mL). The combined organic extracts were washed with brine (50 mL). The organic phase was then dried over Na_2_SO_4_, filtered, and concentrated under reduced pressure to give diketopiperazine **24**. Yield: 1.9 g (>95%). Isolated as an orange crystalline solid. >95% pure by NMR. *R*_*f*_: 0.27 in EtOAc. Optical rotation: [α]_D_^20^: +74.1 (*c* = 0.87, DMSO). ^1^H NMR (DMSO-*d*_6_, 400 MHz): δ 8.58 (d, *J* = 2.4 Hz, 1H), 7.98 (dd, *J* = 8.4, 2.4 Hz, 1H), 7.57 (d, *J* = 8.4 Hz, 1H), 5.13 (t, *J* = 9.6 Hz, 1H), 4.50 (dd, *J* = 16.8, 1.2 Hz, 1H), 3.99 (d, *J* = 16.8 Hz, 1H), 3.53–3.39 (m, 2H), 2.94 (s, 3H) ppm. ^13^C NMR (DMSO-*d*_6_, 101 MHz): δ 166.4, 163.7, 147.0, 141.6, 138.8, 126.0, 120.0, 108.8, 59.8, 53.3, 33.2, 30.8 ppm. FTIR (neat): 2905 (w), 1663 (s), 1598 (w), 1517 (m), 1479 (m), 1415 (m), 1396 (m), 1331 (m), 1215 (m), 1061 (m), 881 (m), 740 (m), 417 (m) cm^–1^. HRMS-ESI (*m*/*z*): [M + Na]^+^ calcd for C_12_H_11_N_3_NaO_4_, 284.0647; found, 284.0645. mp 205–207 °C (obtained by concentration from EtOAc).

### General Procedure for the Attempted Direct Sulfenylation of DKPs

To a stirred suspension of S_8_ (1.0 equiv) in THF was added LiHMDS, NaHMDS, or KHMDS (3.0–4.0 equiv, 1 M in THF) dropwise over 2 min at room temperature under a N_2_ atmosphere. After 5 min, a solution of the diketopiperazine (1.0 equiv) in THF was added dropwise over 2 min. After 5 min, LiHMDS, NaHMDS, or KHMDS (2.0–4.0 equiv, 1 M in THF) was added dropwise over ∼30 s. After 1.5 h, NaHCO_3_ (sat. aq) was added. The resulting mixture was extracted with CH_2_Cl_2_. The combined organic extracts were passed through a phase separator and concentrated under reduced pressure. The resulting crude residue was purified by column chromatography (50–100% EtOAc/*n*-heptane).

### (±)-3-(Hydroxymethyl)-2-methyl-7-nitro-2,3-dihydropyrazino[1,2-*a*]indole-1,4-dione (*rac*-**25**)

Following the general procedure using diketopiperazine **11** (29.1 mg, 100 μmol), S_8_ (25.6 mg, 100 μmol), and LiHMDS (800 μL, 1 M in THF). Yield: 2 mg (7%). Isolated as a yellow amorphous solid. >95% pure by NMR and a single spot by TLC. *R*_*f*_: 0.09 in 75% EtOAc/*n*-heptane. ^1^H NMR (DMSO-*d*_6_, 400 MHz): δ 9.11 (d, *J* = 2.4 Hz, 1H), 8.27 (dd, *J* = 8.8 Hz, 2.4 Hz, 1H), 8.02 (d, *J* = 8.8 Hz, 1H), 7.51 (s, 1H), 5.44 (app. t, *J* = 6.0, 5.6 Hz, 1H), 4.66–4.61 (m, 1H), 4.06–3.97 (m, 1H), 3.92–3.84 (m, 1H), 3.06 (s, 3H) ppm. ^13^C NMR (DMSO-*d*_6_, 101 MHz): δ 166.0, 156.1, 146.4, 135.0, 134.4, 133.1, 123.8, 120.3, 111.8, 111.3, 66.7, 61.4, 31.8 ppm. FTIR (film): 3385 (br), 2930 (w), 1720 (m), 1648 (m), 1592 (w), 1580 (w), 1522 (m), 1468 (m), 1433 (m), 1399 (s), 1340 (w), 1244 (w), 1219 (w), 1119 (w), 1065 (w), 1039 (w), 895 (w), 840 (w), 754 (w), 730 (w) cm^–1^. HRMS-ESI (*m*/*z*): [M + Na]^+^ calcd for C_13_H_11_N_3_NaO_5_, 312.0596; found, 312.0598. mp 267–269 °C (obtained by concentration from EtOAc/*n*-heptane).

### (±)-3-(Hydroxymethyl)-2-methyl-2,3-dihydropyrazino[1,2-*a*]indole-1,4-dione (*rac*-**26**)

Following the general procedure using diketopiperazine **21** (24.6 mg, 100 μmol), S_8_ (25.6 mg, 100 μmol), and LiHMDS (800 μL, 1 M in THF). Yield: 9 mg (37%). Isolated as a white amorphous solid. >95% pure by NMR and a single spot by TLC. *R*_*f*_: 0.11 in 75% EtOAc/*n*-heptane. ^1^H NMR (DMSO-*d*_6_, 400 MHz): δ 8.35 (d, *J* = 8.0 Hz, 1H), 7.78 (d, *J* = 8.0 Hz, 1H), 7.52 (dt, *J* = 8.0, 1.2 Hz, 1H), 7.41 (dt, *J* = 8.0 Hz, 1.2 Hz, 1H), 7.35 (s, 1H), 5.37 (app. t, *J* = 6.0, 5.6 Hz, 1H), 4.56–4.50 (m, 1H), 4.00 (ddd, *J* = 11.6, 7.6, 2.8 Hz, 1H), 3.88 (ddd, *J* = 11.6, 7.6, 1.6 Hz, 1H), 3.04 (s, 3H) ppm. ^13^C NMR (DMSO-*d*_6_, 101 MHz): δ 165.1, 156.3, 134.0, 130.0, 128.8, 127.1, 124.9, 122.5, 115.7, 111.6, 66.1, 60.7, 31.1 ppm. FTIR (film): 3368 (br), 2933 (w), 1711 (m), 1638 (m), 1589 (m), 1574 (w), 1447 (m), 1396 (s), 1337 (m), 1250 (w), 1067 (w), 843 (w), 752 (w), 738 (w) cm^–1^. HRMS-ESI (*m*/*z*): [M + H]^+^ calcd for C_13_H_12_N_2_O_3_, 245.0926; found, 245.0923. mp 200–202 °C (obtained by concentration from EtOAc/*n*-heptane).

### 2-Methyl-7-nitro-2,3-dihydropyrazino[1,2-*a*]indole-1,4-dione (**28**)

Following the general procedure using diketopiperazine **24** (26.1 mg, 100 μmol), S_8_ (25.6 mg, 100 μmol), and LiHMDS (500 μL, 1 M in THF). Yield: 4 mg (15%). Isolated as a yellow amorphous solid. >90% pure by NMR and a single spot by TLC. *R*_*f*_: 0.19 in 75% EtOAc/*n*-heptane. ^1^H NMR (CDCl_3_, 400 MHz): δ 9.32 (d, *J* = 2.0 Hz, 1H), 8.29 (dd, *J* = 8.8, 2.0 Hz, 1H), 7.83 (dd, *J* = 8.8, 0.8 Hz, 1H), 7.50 (d, *J* = 0.8 Hz, 1H), 4.52 (s, 2H), 3.21 (s, 3H) ppm. ^13^C NMR (CDCl_3_, 101 MHz): δ 161.2, 155.3, 147.3, 133.8, 133.7, 133.1, 123.0, 120.7, 113.2, 113.0, 53.8, 33.5 ppm. FTIR (film): 3121 (w), 2926 (w), 2855 (w), 1723 (m), 1656 (m), 1589 (w), 1572 (m), 1521 (m), 1494 (m), 1393 (m), 1365 (s), 1349 (s), 1337 (m), 1292 (w), 1249 (w), 1210 (w), 1067 (w), 1031 (w), 907 (w), 853 (w), 840 (w), 731 (m), 670 (w), 632 (w), 550 (w) cm^–1^. HRMS-ESI (*m*/*z*): [M + Na]^+^ calcd for C_12_H_9_N_3_NaO_4_, 282.0491; found, 282.0483. mp 189–191 °C (obtained by concentration from CDCl_3_/*n*-heptane).

### General Procedure for the Oxidation of DKPs

To a stirred solution of the diketopiperazine (1.0 equiv) in CH_2_Cl_2_ was added silver(I)bispyridine permanganate^43^ (4.0–8.0 equiv) in one portion. After 15–24 h, sodium bisulfite (1 M, aq) was added. The resulting mixture was passed through a phase separator, and the aqueous phase was extracted with CH_2_Cl_2_. The combined organic extracts were washed with CuSO_4_ (sat. aq), NH_4_Cl (sat. aq), and brine. The organic phase was then passed through a phase separator and concentrated under reduced pressure. The resulting crude residue was purified by column chromatography (25–100% EtOAc/*n*-heptane).

### (3*S*)-3-{[(*tert*-Butyldiphenylsilyl)oxy]methyl}-10*a*-hydroxy-2-methyl-7-nitro-2,3,10,10*a*-tetrahydropyrazino[1,2-*a*]indole-1,4-dione (**31**)

Following the general procedure using diketopiperazine **30** (265 mg, 500 μmol), CH_2_Cl_2_ (10 mL), and silver(I)bispyridine permanganate (1.54 g, 4.00 mmol). Yield: 157 mg consisting of a difficult to separate 40:60 mixture of **30**/**31** (35%). A purified sample of **31** was isolated as a white solid by exhaustive column chromatography. >90% pure by NMR and a single spot by TLC. *R*_*f*_: 0.09 in 33% EtOAc/*n*-heptane. ^1^H NMR (CDCl_3_, 400 MHz): δ 8.98 (d, *J* = 2.0 Hz, 1H), 8.10 (dd, *J* = 8.4, 2.0 Hz, 1H), 7.66–7.59 (m, 4H), 7.53–7.40 (m, 7H), 5.70 (br, s, 1H), 4.22 (dd, *J* = 10.8, 1.6 Hz, 1H), 4.08 (dd, *J* = 3.2, 1.6 Hz, 1H), 3.94 (dd, *J* = 11.2, 3.2 Hz, 1H), 3.76 (d, *J* = 18.0 Hz, 1H), 3.47 (d, *J* = 18.0 Hz, 1H), 2.93 (s, 3H), 1.02 (s, 9H) ppm. ^13^C NMR (CDCl_3_, 101 MHz): δ 165.4, 163.8, 148.2, 140.7, 136.4, 135.73, 135.65, 130.9, 130.83, 130.81, 130.5, 128.4, 125.6, 121.1, 111.8, 89.1, 66.4, 62.4, 39.8, 32.3, 26.9, 19.2 ppm. FTIR (film): 3321 (br), 2957 (w), 2932 (w), 2859 (w), 1685 (w), 1602 (w), 1528 (m), 1483 (m), 1436 (m), 1404 (m), 1349 (m), 1240 (w), 1184 (w), 1150 (m), 1112 (m), 1089 (w), 1065 (w), 1032 (w), 997 (w), 911 (w), 883 (w), 825 (w), 740 (m), 703 (m), 615 (w), 505 (w), 491 (w) cm^–1^. HRMS-ESI (*m*/*z*): [M + Na]^+^ calcd for C_29_H_31_NaN_3_O_6_Si, 568.1880; found, 568.1879. mp 96–100 °C (obtained by concentration from CH_2_Cl_2_/*n*-heptane).

### [(3*S*)-10*a*-Hydroxy-2-methyl-7-nitro-1,4-dioxo-1,2,3,4,10,10*a*-hexahydropyrazino(1,2-*a*)indol-3-yl]methyl Acetate (**33**)

Following the general procedure using diketopiperazine **18** (167 mg, 500 μmol), CH_2_Cl_2_ (10 mL), and silver(I)bispyridine permanganate (1.54 g, 4.00 mmol). Yield: 39 mg (22%). Isolated as a white amorphous solid. >95% pure by NMR and a single spot by TLC. *R*_*f*_: 0.23 in 75% EtOAc/*n*-heptane. ^1^H NMR (CDCl_3_, 400 MHz): δ 8.87 (d, *J* = 2.4 Hz, 1H), 8.08 (dd, *J* = 8.0, 2.4 Hz, 1H), 7.46 (d, *J* = 8.0 Hz, 1H), 4.77 (br, s, 1H), 4.72 (dd, *J* = 11.6, 4.4 Hz, 1H), 4.64 (dd, *J* = 11.6, 4.4 Hz, 1H), 4.36 (t, *J* = 4.4 Hz, 1H), 3.76 (dd, *J* = 18.4, 1.6 Hz, 1H), 3.46 (d, *J* = 18.4 Hz, 1H), 3.10 (s, 3H), 2.12 (s, 3H) ppm. ^13^C NMR (CDCl_3_, 101 MHz): δ 169.8, 165.1, 163.0, 148.2, 140.6, 135.5, 125.5, 121.3, 112.0, 89.3, 64.0, 63.2, 40.9, 33.4, 20.8 ppm. FTIR (film): 3291 (br), 2952 (w), 2931 (w), 1747 (m), 1686 (s), 1656 (s), 1603 (w), 1526 (s), 1483 (m), 1438 (m), 1406 (m), 1349 (s), 1222 (s), 1185 (w), 1153 (w), 1059 (m), 912 (w), 885 (w), 831 (w), 740 (m), 638 (w) cm^–1^. HRMS-ESI (*m*/*z*): [M + Na]^+^ calcd for C_15_H_15_NaN_3_O_7_, 372.0808; found, 372.0806. mp 102–105 °C (obtained by concentration from EtOAc/*n*-heptane).

### [3,10*a*-Dihydroxy-2-methyl-7-nitro-1,4-dioxo-1,2,3,4,10,10*a*-hexahydropyrazino(1,2-*a*)indol-3-yl]methyl Acetate (**34**)

Following the general procedure using diketopiperazine **18** (167 mg, 500 μmol), CH_2_Cl_2_ (10 mL), and silver(I)bispyridine permanganate (1.54 g, 4.00 mmol). Yield: 14 mg (8%). Isolated as a yellow amorphous solid. >95% pure by NMR and a single spot by TLC. *R*_*f*_: 0.19 in 75% EtOAc/*n*-heptane. ^1^H NMR (DMSO-*d*_6_, 400 MHz): δ 8.65 (d, *J* = 2.2 Hz, 1H), 8.10 (dd, *J* = 8.4, 2.2 Hz, 1H), 7.69 (d, *J* = 8.4 Hz, 1H), 7.56 (s, 1H), 7.42 (s, 1H), 4.64 (d, *J* = 11.2 Hz, 1H), 4.32 (d, *J* = 11.2 Hz, 1H), 3.68 (d, *J* = 18.4 Hz, 1H), 3.39 (d, *J* = 18.4 Hz, 1H), 2.97 (s, 3H), 1.89 (s, 3H) ppm. ^13^C NMR (DMSO-*d*_6_, 101 MHz): δ 169.4, 166.4, 163.5, 147.1, 140.5, 137.3, 126.3, 120.7, 110.2, 88.2, 84.9, 63.5, 40.6, 27.3, 20.3 ppm. FTIR (film): 3349 (br), 2979 (w), 2924 (w), 1750 (m), 1699 (s), 1605 (w), 1528 (m), 1486 (w), 1437 (m), 1396 (m), 1350 (m), 1257 (m), 1157 (m), 1060 (m), 913 (w), 828 (w), 739 (w) cm^–1^. HRMS-ESI (*m*/*z*): [M + Na]^+^ calcd for C_15_H_15_NaN_3_O_8_, 388.0757; found, 388.0758. mp 70–74 °C (obtained by concentration from CH_2_Cl_2_/*n*-heptane).

### [3,10*a*-Dihydroxy-2-methyl-1,4-dioxo-1,2,3,4,10,10*a*-hexahydropyrazino(1,2-*a*)indol-3-yl]methyl Acetate (**36**)

Following the general procedure using diketopiperazine **35** (72.7 mg, 150 μmol), CH_2_Cl_2_ (3 mL), and silver(I)bispyridine permanganate (231 mg, 600 μmol). Yield: 36 mg (46%). Isolated as yellow foam. >95% pure by NMR and a single spot by TLC. *R*_*f*_: 0.21 in 50% EtOAc/*n*-heptane. ^1^H NMR (CDCl_3_, 400 MHz): δ 8.00 (d, *J* = 7.6 Hz, 1H), 7.63–7.52 (m, 4H), 7.50–7.20 (m, 8H), 7.15 (t, *J* = 7.2 Hz, 1H), 5.22 (br, s, 1H), 5.00 (br, s, 1H), 4.29 (d, *J* = 10.8 Hz, 1H), 4.02 (d, *J* = 10.8 Hz, 1H), 3.69 (d, *J* = 17.2 Hz, 1H), 3.35 (d, *J* = 17.2 Hz, 1H), 3.01 (s, 3H), 0.96 (s, 9H) ppm. ^13^C NMR (CDCl_3_, 101 MHz): δ 168.2, 163.4, 139.9, 135.8, 135.7, 132.3, 132.1, 130.3, 130.2, 128.17, 128.10, 128.06, 128.0, 125.7, 125.3, 116.8, 88.6, 86.7, 64.2, 40.3, 28.2, 26.8, 19.3 ppm. FTIR (film): 3339 (br), 3072 (w), 3051 (w), 2956 (w), 2930 (w), 2894 (w), 2858 (w), 1688 (s), 1663 (s), 1606 (w), 1482 (m), 1463 (m), 1427 (s), 1396 (m), 1363 (w), 1320 (w), 1273 (w), 1186 (w), 1113 (s), 1056 (m), 912 (w), 822 (m), 777 (w), 753 (m), 735 (s), 613 (w), 505 (m), 489 (w) cm^–1^. HRMS-ESI (*m*/*z*): [M + Na]^+^ calcd for C_29_H_32_N_2_NaO_5_Si, 539.1973; found, 539.1978.

### (±)-3,10*a*-Dihydroxy-2-methyl-7-nitro-2,3,10,10*a*-tetrahydropyrazino[1,2-*a*]indole-1,4-dione (*rac*-**37**)

Following the general procedure using diketopiperazine **24** (52.0 mg, 200 μmol), CH_2_Cl_2_ (4 mL), and silver(I)bispyridine permanganate (385 mg, 1.00 mmol). Yield: 7 mg (12%). Isolated as a yellow amorphous solid. >95% pure by NMR and a single spot by TLC. *R*_*f*_: 0.10 in 75% EtOAc/*n*-heptane. ^1^H NMR (DMSO-*d*_6_, 400 MHz): δ 8.61 (d, *J* = 2.4 Hz, 1H), 8.06 (dd, *J* = 8.4, 2.4 Hz, 1H), 7.64 (d, *J* = 8.4 Hz, 1H), 7.45 (s, 1H), 7.37 (d, *J* = 6.6 Hz, 1H), 5.21 (d, *J* = 6.6 Hz, 1H), 3.65 (d, *J* = 18.4 Hz, 1H), 3.31 (d, *J* = 18.4 Hz, 1H), 2.96 (s, 3H) ppm. ^13^C NMR (DMSO-*d*_6_, 101 MHz): δ 165.9, 164.0, 147.0, 140.6, 137.3, 126.1, 120.3, 109.7, 88.7, 81.5, 40.6, 31.2 ppm. FTIR (film): 3258 (br), 2924 (w), 2854 (w), 1690 (s), 1667 (m), 1602 (w), 1526 (m), 1482 (m), 1439 (w), 1404 (m), 1350 (m), 1242 (w), 1185 (w), 1149 (w), 1068 (w), 1046 (m), 1027 (m), 940 (w), 884 (w), 829 (w), 777 (w), 740 (w) cm^–1^. HRMS-ESI (*m*/*z*): [M – H]^+^ calcd for C_12_H_11_N_3_O_6_, 292.0570; found, 292.0572. mp 200–202 °C (obtained by concentration from CH_2_Cl_2_/*n*-heptane).

### (3*S*)-10*a*-Hydroxy-3-(hydroxymethyl)-2-methyl-7-nitro-2,3,10,10*a*-tetrahydropyrazino[1,2-*a*]indole-1,4-dione (**32**)

To a stirred 40:60 mixture of **30**/**31** (70.0 mg) in pyridine (1.5 mL) was added pyridine hydrofluoride (500 μL). After 2 h, the resulting reaction mixture was neutralized with NaHCO_3_ (s). The resulting mixture was diluted with water and extracted with EtOAc (3 × 10 mL). The combined organic extracts were washed with CuSO_4_ (2 × 10 mL, sat. aq) and brine (10 mL). The organic phase was then dried over Na_2_SO_4_, filtered, and concentrated under reduced pressure. The resulting crude residue was purified by column chromatography (50–100% EtOAc/*n*-heptane) to give diol **32**. Yield: 11 mg (28%). Isolated as a white crystalline solid. >95% pure by NMR and a single spot by TLC. *R*_*f*_: 0.12 in 75% EtOAc/*n*-heptane. ^1^H NMR (DMSO-*d*_6_, 400 MHz): δ 8.67 (d, *J* = 2.4 Hz, 1H), 8.06 (dd, *J* = 8.2, 2.4 Hz, 1H), 7.63 (d, *J* = 8.2 Hz, 1H), 7.10 (s, 1H), 6.53 (t, *J* = 4.4 Hz, 1H), 4.37 (app. t, *J* = 4.0, 3.2 Hz, 1H), 4.04–3.91 (m, 2H), 3.66 (dd, *J* = 18.4, 1H), 3.29 (d, *J* = 18.4 Hz, 1H), 2.99 (s, 3H) ppm. ^13^C NMR (DMSO-*d*_6_, 101 MHz): δ 165.3, 164.7, 147.0, 140.5, 137.2, 126.1, 120.3, 109.7, 88.8, 66.1, 60.8, 32.4 ppm. The signal corresponding to the benzylic carbon atom coincides with the residual DMSO signal. FTIR (film): 3259 (br), 2927 (w), 1681 (s), 1603 (w), 1526 (m), 1483 (m), 1437 (m), 1405 (m), 1348 (m), 1348 (m), 1243 (w), 1203 (w), 1185 (w), 1148 (w), 1107 (w), 1085 (w), 1066 (w), 908 (w), 887 (w), 830 (w), 818 (w), 741 (w) cm^–1^. HRMS-ESI (*m*/*z*): [M + Na]^+^ calcd for C_13_H_13_N_3_NaO_6_, 330.0702; found, 330.0692. mp 239–240 °C (obtained by recrystallization from EtOH).

### General Procedure for the Sulfenylation of DKP Hemi-Aminals

To a stirred solution of the hemi-aminal (1.0 equiv) and 4-methoxy-α-toluene thiol (10.0 equiv) in CH_2_Cl_2_ under a N_2_ atmosphere was added dropwise BF_3_·Et_2_O (20.0 equiv). After 1 h, NaHCO_3_ (sat. aq) was added, and the resulting mixture was extracted with CH_2_Cl_2_. The combined organic extracts were passed through a phase separator and concentrated under reduced pressure. The resulting crude residue was purified by column chromatography (25–50% EtOAc/*n*-heptane).

### (*S*)-3-{[(*tert*-Butyldiphenylsilyl)oxy]methyl}-2-methyl-7-nitro-2,3-dihydropyrazino[1,2-*a*]indole-1,4-dione (**38**)

Following the general procedure using hemi-aminal **31** (14.0 mg, 25.7 μmol), 4-methoxy-α-toluene thiol (33 μL, 257 μmol), CH_2_Cl_2_, (1 mL), and BF_3_·Et_2_O (69 μL, 513 mmol). Yield: 13 mg (>95%). Isolated as a white/yellow amorphous solid. >95% pure by NMR and a single spot by TLC. *R*_*f*_: 0.13 in 33% EtOAc/*n*-heptane. Optical rotation: [α]_D_^20^: −181 (*c* = 1.0 in CH_2_Cl_2_). ^1^H NMR (CDCl_3_, 400 MHz): δ 9.33 (d, *J* = 2.2 Hz, 1H), 8.28 (dd, *J* = 8.8, 2.2 Hz, 1H), 7.81 (d, *J* = 8.8 Hz, 1H), 7.50–7.22 (m, 11H), 4.33 (app. t, *J* = 2.4, 2.0 Hz, 1H), 4.21 (dd, *J* = 10.8, 2.0 Hz, 1H), 4.05 (dd, *J* = 10.8, 2.4 Hz, 1H), 3.03 (s, 3H), 0.69 (s, 9H) ppm. ^13^C NMR (CDCl_3_, 101 MHz): δ 164.2, 156.6, 147.1, 135.5, 135.4, 134.1, 133.9, 133.5, 131.9, 131.7, 130.3, 130.2, 128.03, 127.97, 122.8, 120.5, 112.7, 112.4, 66.8, 63.2, 32.0, 26.3, 18.8 ppm. FTIR (film): 3122 (w), 3072 (w), 2953 (w), 2931 (w), 2886 (w), 2858 (w), 1723 (m), 1660 (m), 1591 (w), 1579 (w), 1522 (m), 1470 (m), 1429 (m), 1396 (m), 1360 (s), 1291 (w), 1245 (w), 1210 (w), 1105 (m), 1068 (w), 1045 (w), 931 (w), 908 (w), 854 (w), 730 (s), 702 (m), 616 (w), 504 (w), 489 (m), 429 (w) cm^–1^. HRMS-ESI (*m*/*z*): [M + H]^+^ calcd for C_29_H_29_N_3_O_5_Si, 528.1955; found, 528.1961. mp 69–77 °C (obtained by concentration from CH_2_Cl_2_/*n*-heptane).

### (±)-(3-((4-Methoxybenzyl)thio)-2-methyl-7-nitro-1,4-dioxo-1,2,3,4-tetrahydropyrazino[1,2-*a*]indol-3-yl)methyl Acetate (*rac*-**39**)

Following the general procedure using bis-hemi-aminal **34** (14.0 mg, 38.3 μmol), 4-methoxy-α-toluene thiol (49 μL, 383 μmol), CH_2_Cl_2_ (1 mL), and BF_3_^·^Et_2_O (103 μL, 766 mmol). Yield: 11 mg (60%). Isolated as a yellow crystalline solid. >95% pure by NMR and a single spot by TLC. *R*_*f*_: 0.29 in 50% EtOAc/*n*-heptane. ^1^H NMR (CDCl_3_, 400 MHz): δ 9.01 (d, *J* = 2.4 Hz, 1H), 8.26 (dd, *J* = 8.8, 2.4 Hz, 1H), 7.77 (d, *J* = 8.8 Hz, 1H), 7.45 (s, 1H), 6.98–6.90 (m, 2H), 6.39–6.28 (m, 2H), 4.76–4.67 (m, 2H), 3.79 (d, *J* = 14.8 Hz, 1H), 3.57 (d, *J* = 14.8 Hz, 1H), 3.44 (s, 3H), 3.32 (s, 3H), 1.92 (s, 3H) ppm. ^13^C NMR (CDCl_3_, 101 MHz): δ 169.6, 162.1, 158.9, 156.3, 147.1, 133.6, 133.5, 131.5, 129.2, 126.4, 122.7, 120.6, 113.6, 113.3, 113.2, 74.1, 64.2, 54.9, 34.2, 28.6, 20.6 ppm. FTIR (film): 3124 (w), 2954 (w), 2837 (w), 1751 (m), 1751 (m), 1720 (m), 1663 (m), 1609 (w), 1580 (w), 1522 (m), 1512 (m), 1464 (m), 1422 (m), 1397 (m), 1358 (s), 1341 (s), 1250 (m), 1210 (m), 1177 (m), 1037 (w), 897 (w), 840 (w), 731 (m) cm^–1^. HRMS-ESI (*m*/*z*): [M + H]^+^ calcd for C_23_H_21_N_3_O_7_S, 484.1178; found, 484.1175. mp 176–179 °C (obtained by concentration from EtOAc/*n*-heptane).

### (±)-3-{[(*tert*-Butyldiphenylsilyl)oxy]methyl}-3-[(4-methoxybenzyl)thio]-2-methyl-2,3-dihydropyrazino[1,2-*a*]indole-1,4-dione (*rac*-**40**)

Following the general procedure using bis-hemi-aminal **36** (36.0 mg, 69.8 μmol), 4-methoxy-α-toluene thiol (97 μL, 698 μmol), CH_2_Cl_2_ (2 mL), and BF_3_·Et_2_O (172 μL, 1.40 mmol). Yield: 33 mg (74%). Isolated as colorless oil. >95% pure by NMR and a single spot by TLC. *R*_*f*_: 0.17 in 25% EtOAc/*n*-heptane. ^1^H NMR (CDCl_3_, 400 MHz): δ 8.30–8.25 (m, 1H), 7.71–7.66 (m, 1H), 7.53–7.23 (m, 13H), 6.98–6.91 (m, 2H), 6.38–6.31 (m, 2H), 4.29 (d, *J* = 10.0 Hz, 1H), 3.84 (d, *J* = 10.0 Hz, 1H), 3.66 (d, *J* = 14.0 Hz, 1H), 3.53 (d, *J* = 14.0 Hz, 1H), 3.38 (s, 3H), 3.19 (s, 3H), 0.74 (s, 9H) ppm. ^13^C NMR (CDCl_3_, 101 MHz): δ 163.5, 158.7, 157.7, 135.6, 135.1, 132.4, 132.1, 130.2, 130.1, 129.7, 129.0, 128.04, 128.01, 127.9, 127.8, 127.2, 125.3, 122.3, 117.0, 114.1, 113.6, 77.7, 65.9, 54.9, 34.0, 28.4, 26.4, 19.1 ppm. FTIR (film): 3071 (w), 2953 (w), 2931 (w), 2893 (w), 2857 (w), 1711 (m), 1656 (w), 1609 (w), 1589 (w), 1574 (w), 1449 (m), 1424 (s), 1353 (s), 1303 (w), 1251 (m), 1222 (w), 1204 (w), 1176 (w), 1107 (s), 1035 (w), 908 (w), 865 (w), 822 (m), 735 (s), 702 (s), 611 (w), 504 (m), 488 (w) cm^–1^. HRMS-ESI (*m*/*z*): [M + Na]^+^ calcd for C_37_H_38_N_2_NaSSi, 657.2219; found, 657.2224.

## Supporting Information Available

Crystallographic information for **11**, optimization of the C–H activation reaction to form **12** from **16**, and copies of ^1^H and ^13^C NMR spectra of **9**, **11**–**13**, **16**–**21**, **23**–**26**, **28**, and **30**–**42** (PDF)
Crystallographic data for DKP-11 (CIF)

## Supplementary Material

ao2c00810_si_001.pdf

ao2c00810_si_002.cif
