# Supplementary material for: A C–H Activation Approach to the Tricyclic Core of Glionitrin A and B
Source: ACS Omega. 2022 Apr 4;7(14):12329–41. doi: 10.1021/acsomega.2c00810 (PMC9016890; doi:10.1021/acsomega.2c00810)

Supporting Information

A C-H Activation Approach to the Tricyclic Core of Glionitrin A and B

Nicolas R. Koning and Daniel Strand*

Centre for Analysis and Synthesis, Department of Chemistry, Lund university Box 124,

SE-221 00 Lund, Sweden.

Index

I. *Optimization of the synthesis of indoline* ***12*** *S3*

II. *Single crystal X-ray diffraction (scXRD) analysis of* ***11*** *S4*

III. *Copies of* ^{1}*H NMR and* ^{13}*C NMR spectra for* ***9****,* ***11-13****,* ***16-21****,* ***23-26****,* ***28****, and* ***30-42*** *S6*

*I. Optimization of the synthesis of indoline **12***

General procedure: To a mixture of dipeptide **16** (64.9 mg, 100 µmol), $Pd(OAc)_2$, and $PhI(OAc)_2$ was added toluene (1 mL). The resulting reaction mixture was heated to 110 °C. After 20 h, the mixture was cooled to room temperature and filtered over celite. The filtrate was then concentrated under reduced pressure and the resulting crude residue was analyzed by ^1H NMR spectroscopy.

Table S1. Optimization of the reaction conditions for conversion of **16** to **12**.

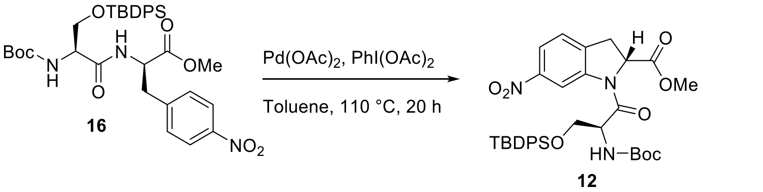

Entry	Catalyst (mol%)	Oxidant (equiv.)	Yield (%)[a]
1	5	2	29
2	5	4	50
3	5	6	50
4	10	2	33
5	20	2	33

[a]Using 1-methoxynaphtalene as internal standard for yields and the following peaks for integration: δ 6.82 (dd, J = 7.2, 1.2 Hz, 1H) and 5.78 (br. d, J = 9.6 Hz, 1H).

*II. Single crystal X-ray diffraction (scXRD) analysis of **11***

Single crystals covered in paratone oil were cut to size and mounted on a MiTeGen micro-mount loop. Data collection was performed on an Agilent Enhance diffractometer equipped with a MoKα high-brilliance IμS radiation source (λ = 0.71073 Å). Absorption was corrected for using multi-scan empirical absorption correction with spherical harmonics as implemented in the SCALE3 ABSPACK scaling algorithm.[1] The structures were solved in WinGX[2] using SUPERFLIP[3] or SHELXL 2016/4[4] and refined using SHELXL 2016/4. Non-hydrogen atoms were refined anisotropically.

[1] CrysAlis PRO. Agilent Technologies 2011.

[2] Farrugia, L. J. *WinGX* and *ORTEP* for *Windows*: an update. *J. Appl. Crystallogr.* **2012**, *45*, 849-854.

[3] Palatinus, L.; Chapuis, G. *SUPERFLIP* – a computer program for the solution of crystal structures by charge flipping in arbitrary dimensions. *J. Appl. Crystallogr.* **2007**, *40*, 786-790.

[4] Sheldrick, G. M. Crystal structure refinement with *SHELXL*. *Acta Crystallogr.* **2015**, *C71*, 3-8.

scXRD data for 11. *Crystallization:* Single crystals suitable for SC-XRD spontaneously formed from a solution of diketopiperazine **11** in methanol-d_4.

Table S2. Crystal data for diketopiperazine **11**.[a,b]

Chemical formula	$C_{13}H_{13}N_3O_5$	
Formula weight	291.26	
Collection temperature /K	293(2)	
Crystal size /mm^3	0.2 × 0.1 × 0.1	
Crystal habit	yellow, irregular	
Wavelength /Å	0.71073	
Crystal system	Orthorhombic	
Space group	$P2_12_12_1$	
Unit cell dimensions:	*a* = 7.1203(6) Å	α = 90 °
	b = 7.5193(7) Å	β = 90 °
	c = 23.4584(18) Å	γ = 90 °
Unit cell volume /Å^3	1255.95(18)	
Z, Calculated density /Mg/m^3	4, 1.540	
Radiation type	MoKα	
Absorption coefficient, m/mm^{-1}	0.121	
No. reflections collected / unique	5917 / 2862	
R_{int}	0.0269	
Completeness to theta = 25.000 /%	99.5	
Data / restraints / parameters	2862 / 0 / 192	
Goodness of fit on F^2	1.046	
Final *R* indices $(I > 2\sigma(I))$	R_1 = 0.0460, wR_2 = 0.0858	
R indices *(all data)*	R_1 = 0.0533, wR_2 = 0.0895	
Absolute structure parameter	-1.7(8)	
Largest diff. peak and hole /e-/Å^3	0.210 and -0.239	
CCDC	2114380	

[a] Absolute configuration was assigned by two unchanging stereocenters in the synthesis (C3a and C10a); [b] For simplicity, the alcohol group was modeled with a hydrogen atom instead of a deuterium atom that would be the result of chemical exchange in methanol-d_4.

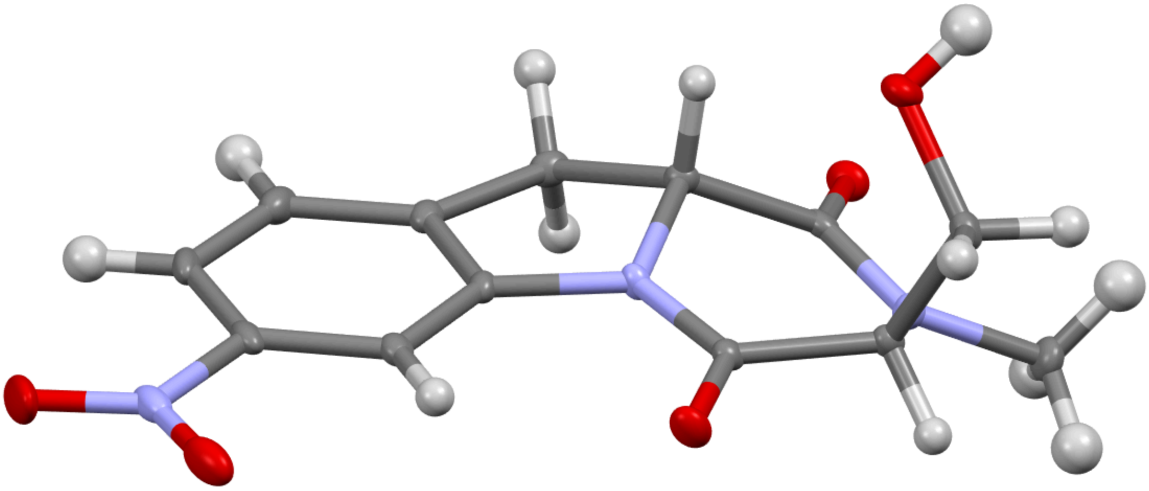

Figure S1. Asymmetric unit of diketopiperazine **11**. Black = carbon atom; blue = nitrogen atom; red = oxygen atom; white = hydrogen atom. Thermal ellipsoids are shown at 30% probability.

*III. Copies of ^{1}H NMR and ^{13}C NMR spectra for **9**, **11-13**, **16-21**, **23-26**, **28**, and **30-42***

^{1}H NMR, 400 MHz, $CDCl_3$

Strand and co-workers 2022

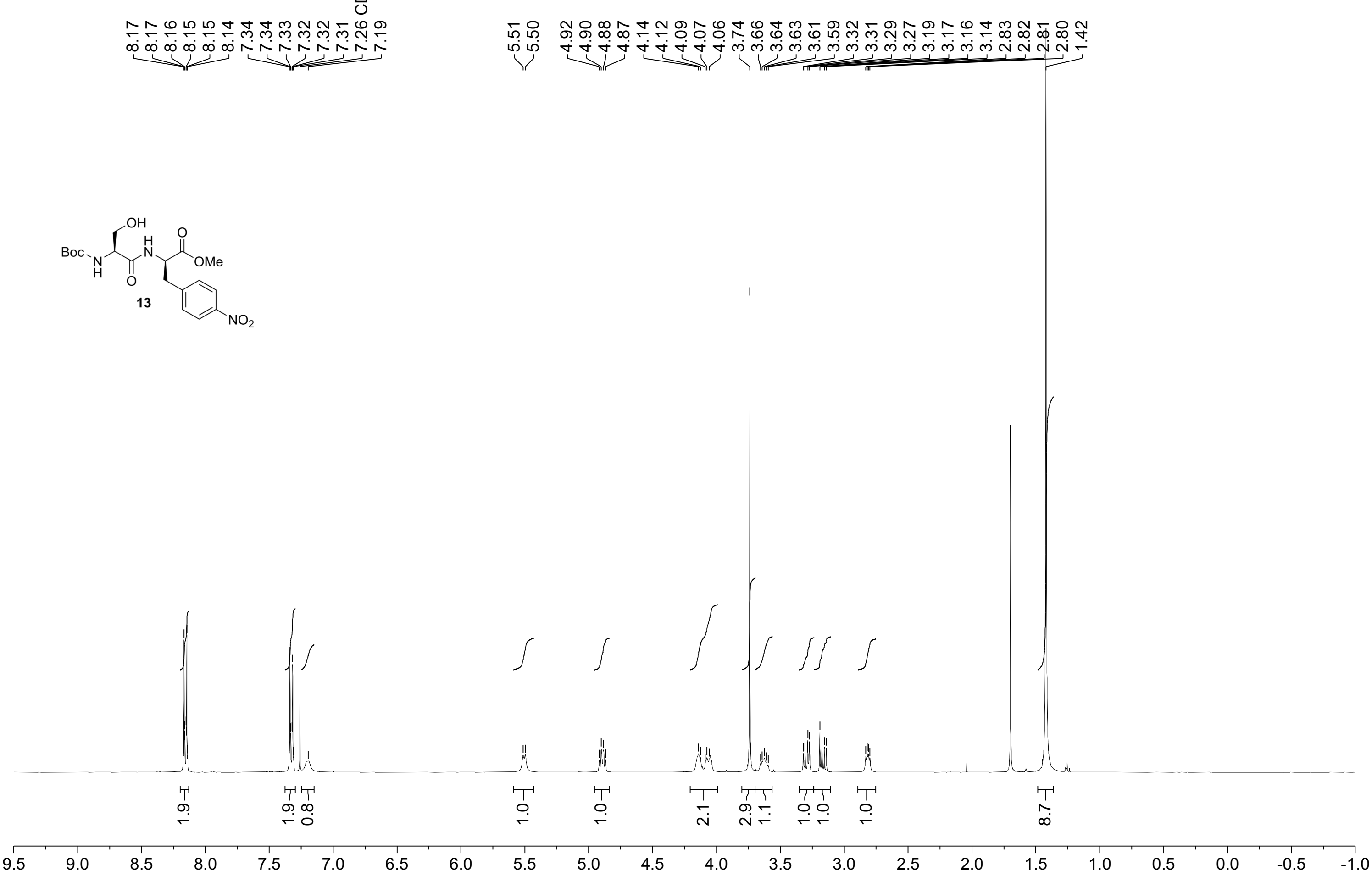

^{13}C NMR, 101 MHz, $CDCl_3$

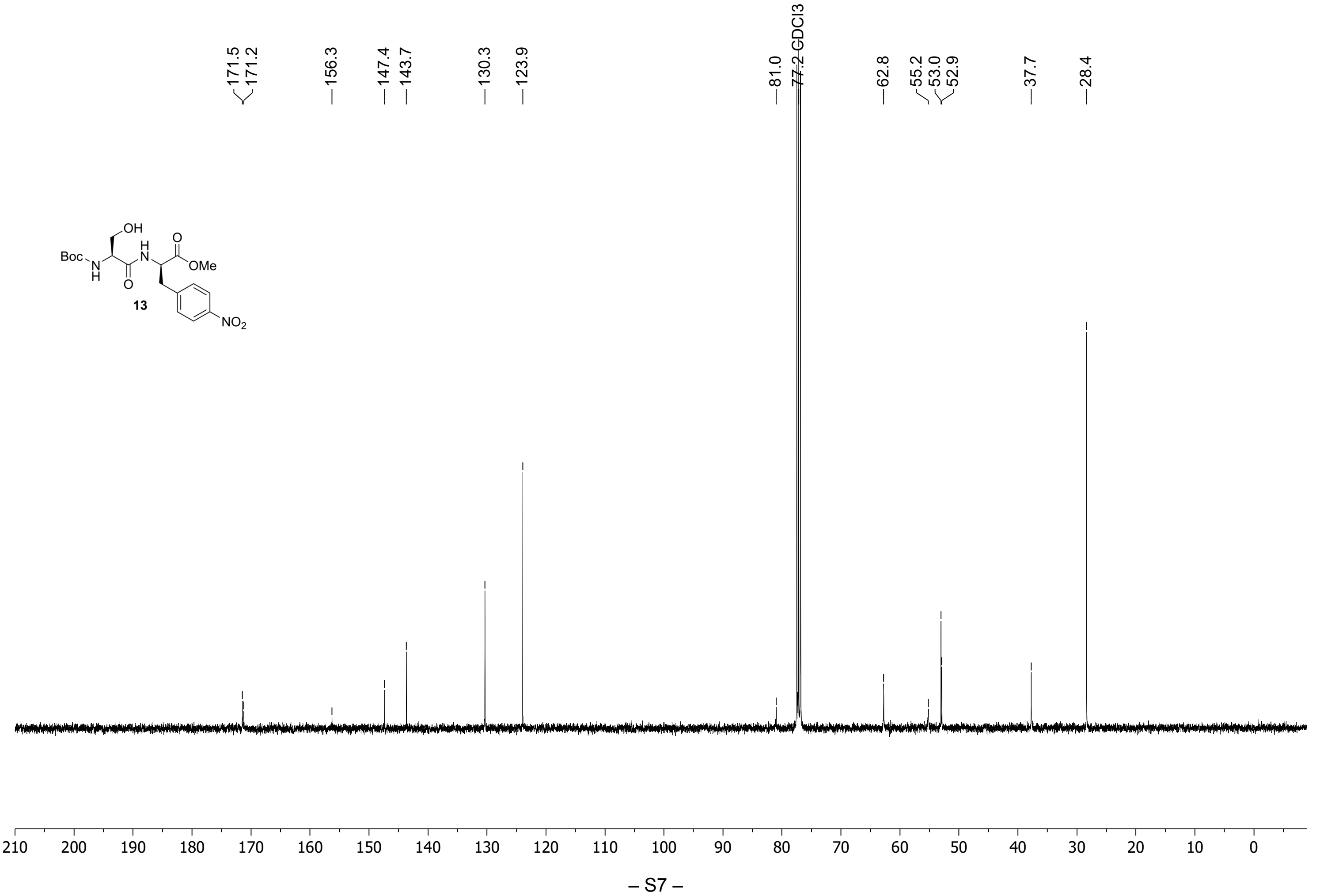

^{1}H NMR, 400 MHz, $CDCl_3$

Strand and co-workers 2022

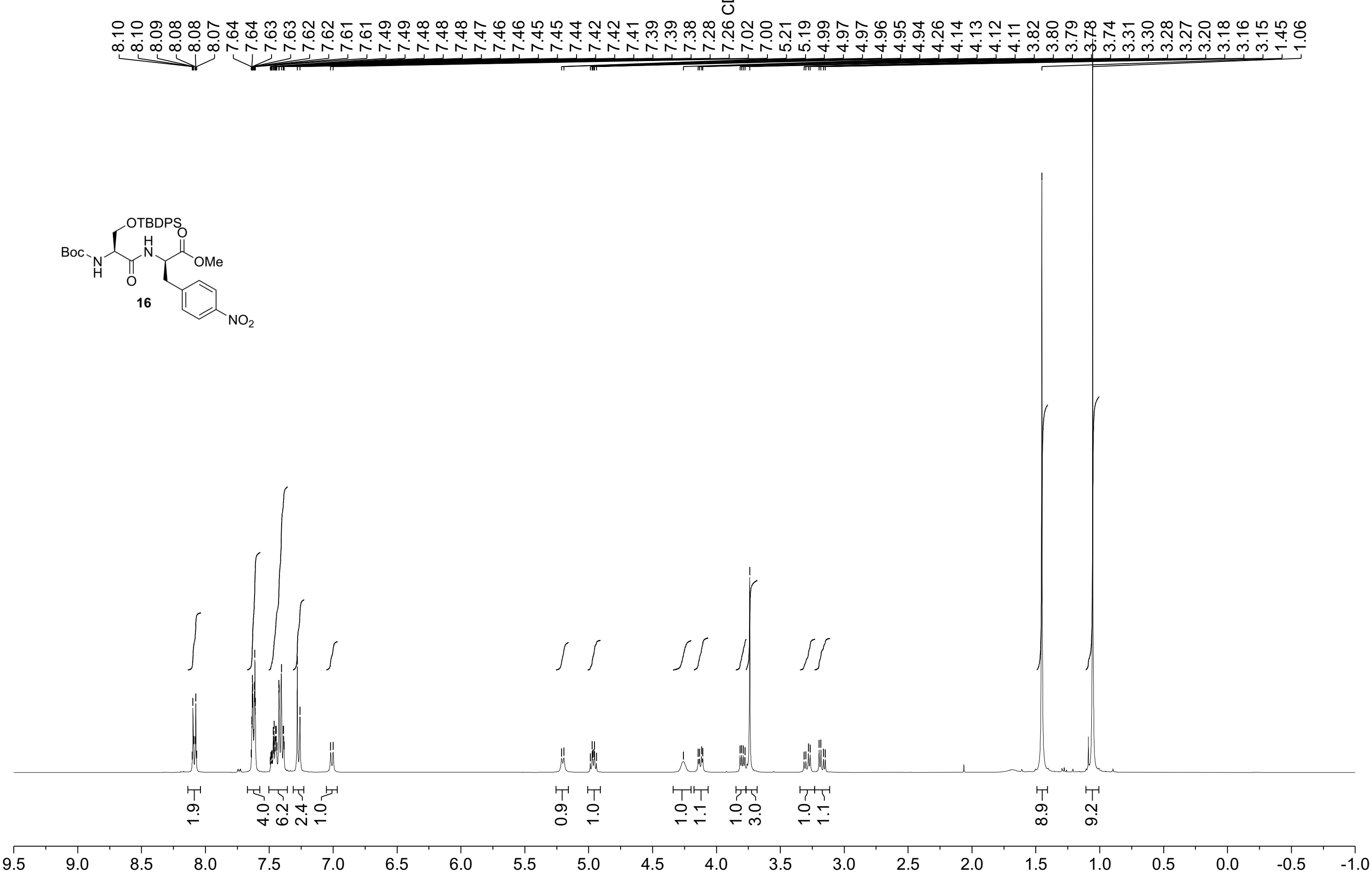

^{13}C NMR, 101 MHz, $CDCl_3$

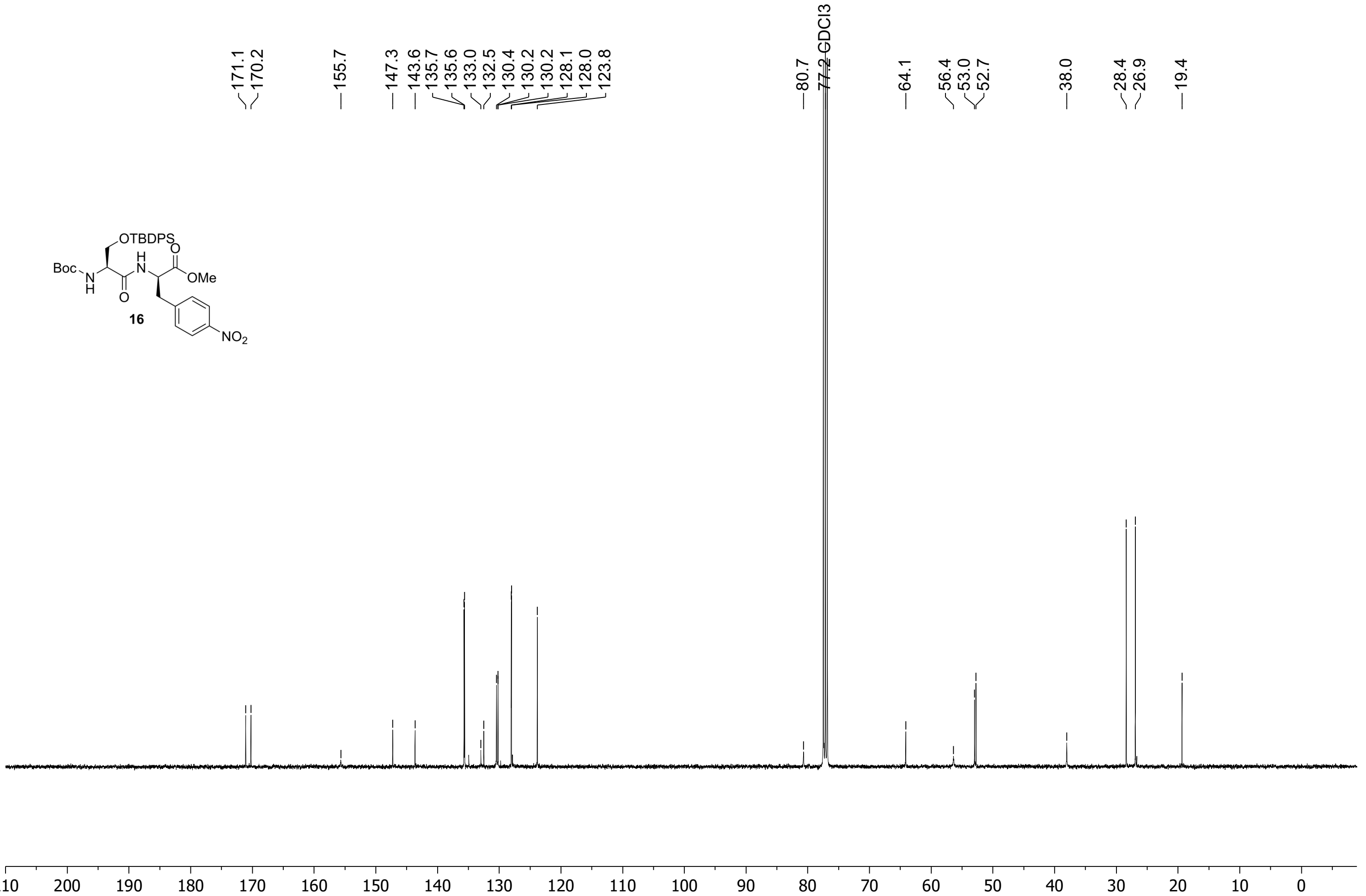

^{1}H NMR, 400 MHz, $CDCl_3$
Strand and co-workers 2022

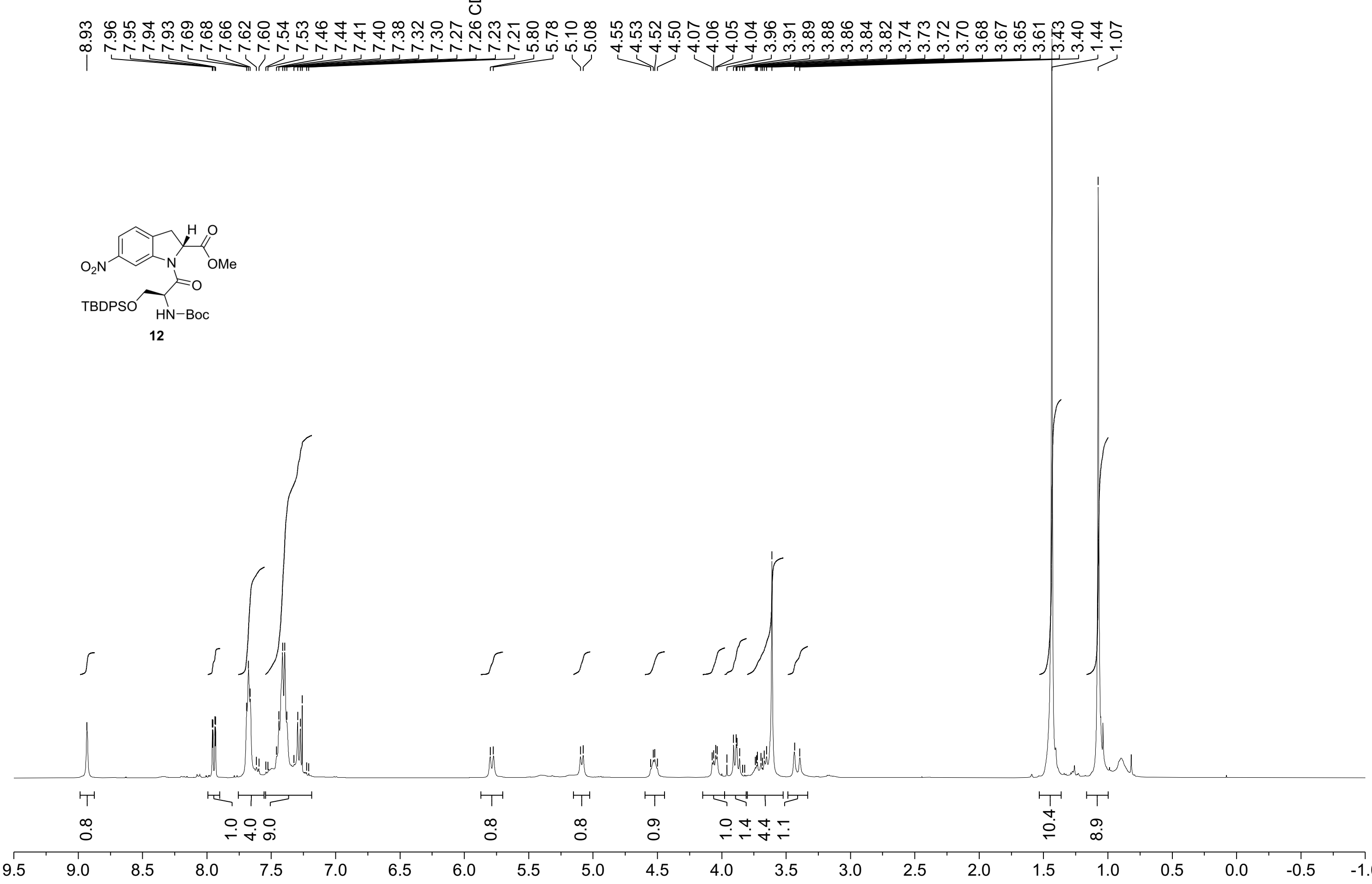

^{13}C NMR, 101 MHz, $CDCl_3$

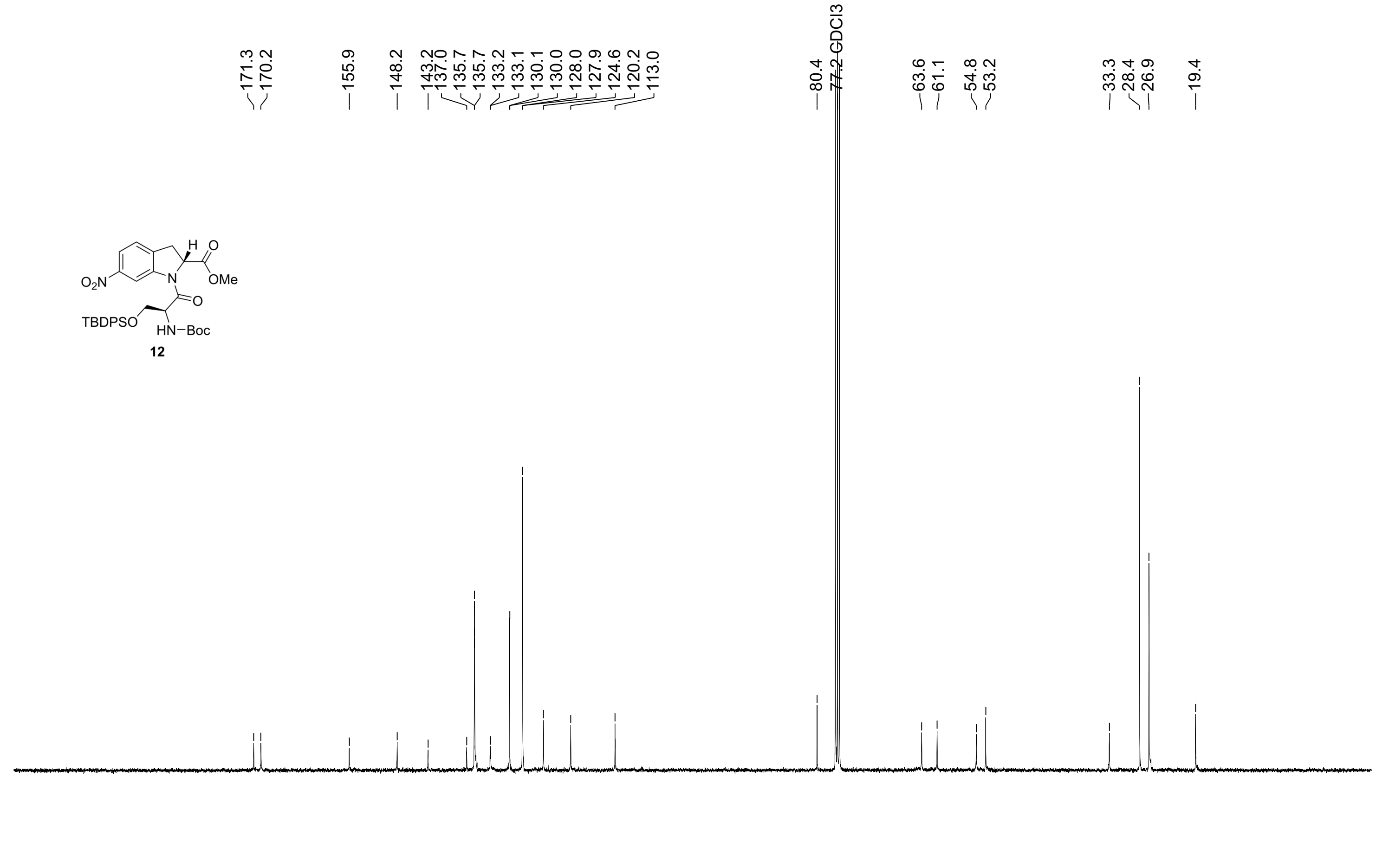

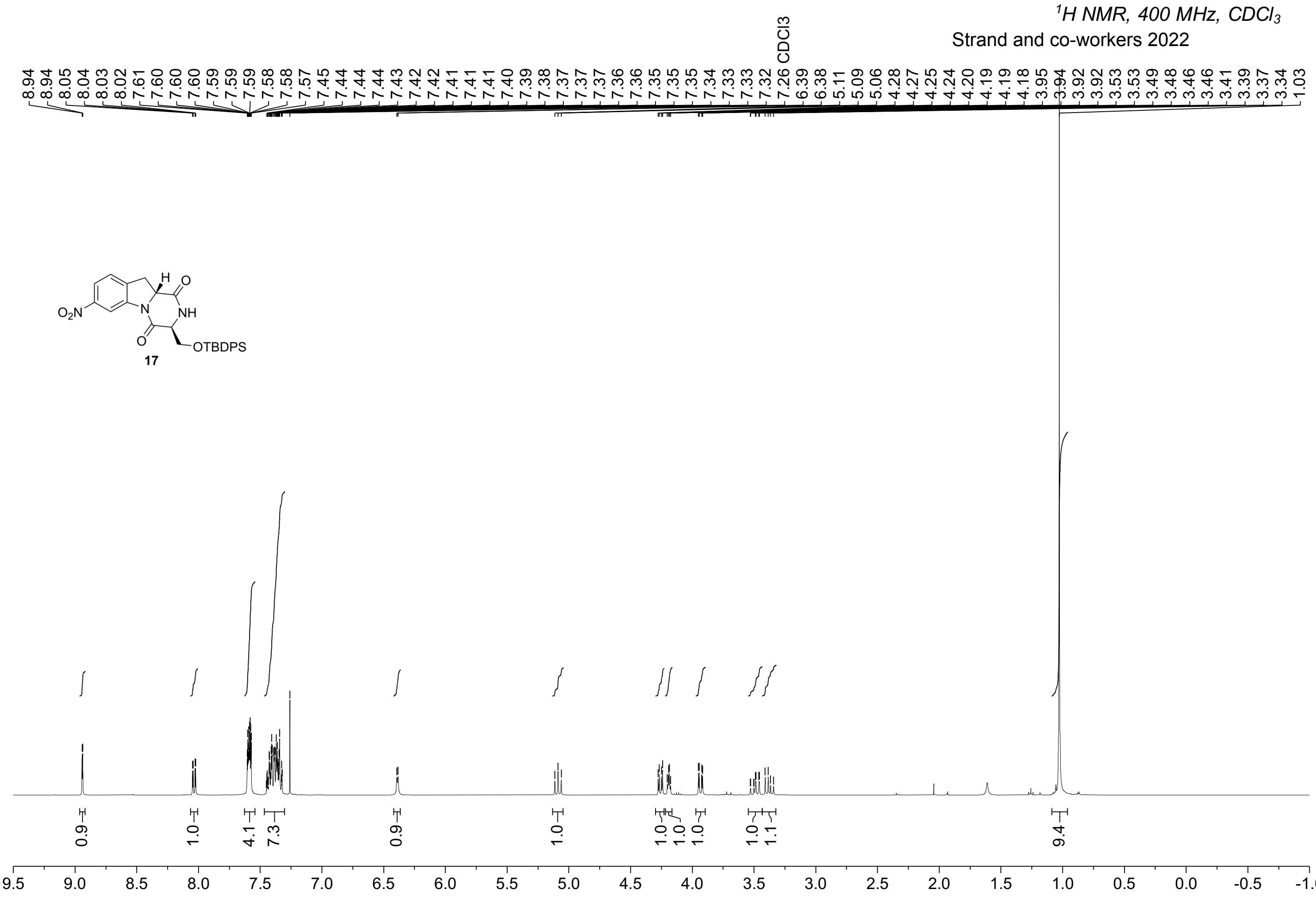
^{1}H NMR, 400 MHz, $CDCl_3$
Strand and co-workers 2022
CDCl3
8.94 8.94 8.05 8.04 8.03 8.02 7.61 7.60 7.60 7.59 7.59 7.59 7.58 7.58 7.57 7.45 7.44 7.44 7.43 7.42 7.42 7.41 7.41 7.41 7.40 7.39 7.38 7.37 7.37 7.37 7.36 7.36 7.35 7.35 7.35 7.34 7.33 7.33 7.32 7.26 6.39 6.38 5.11 5.09 5.06 4.28 4.27 4.25 4.24 4.20 4.19 4.19 4.18 3.95 3.94 3.92 3.92 3.53 3.49 3.48 3.46 3.46 3.41 3.39 3.37 3.34 1.03
O_2N
H
O
NH
N
O
OTBDPS
17
0.9 1.0 4.1 7.3 0.9 1.0 1.0 1.0 1.0 1.0 1.1 9.4
9.5 9.0 8.5 8.0 7.5 7.0 6.5 6.0 5.5 5.0 4.5 4.0 3.5 3.0 2.5 2.0 1.5 1.0 0.5 0.0 -0.5 -1.0

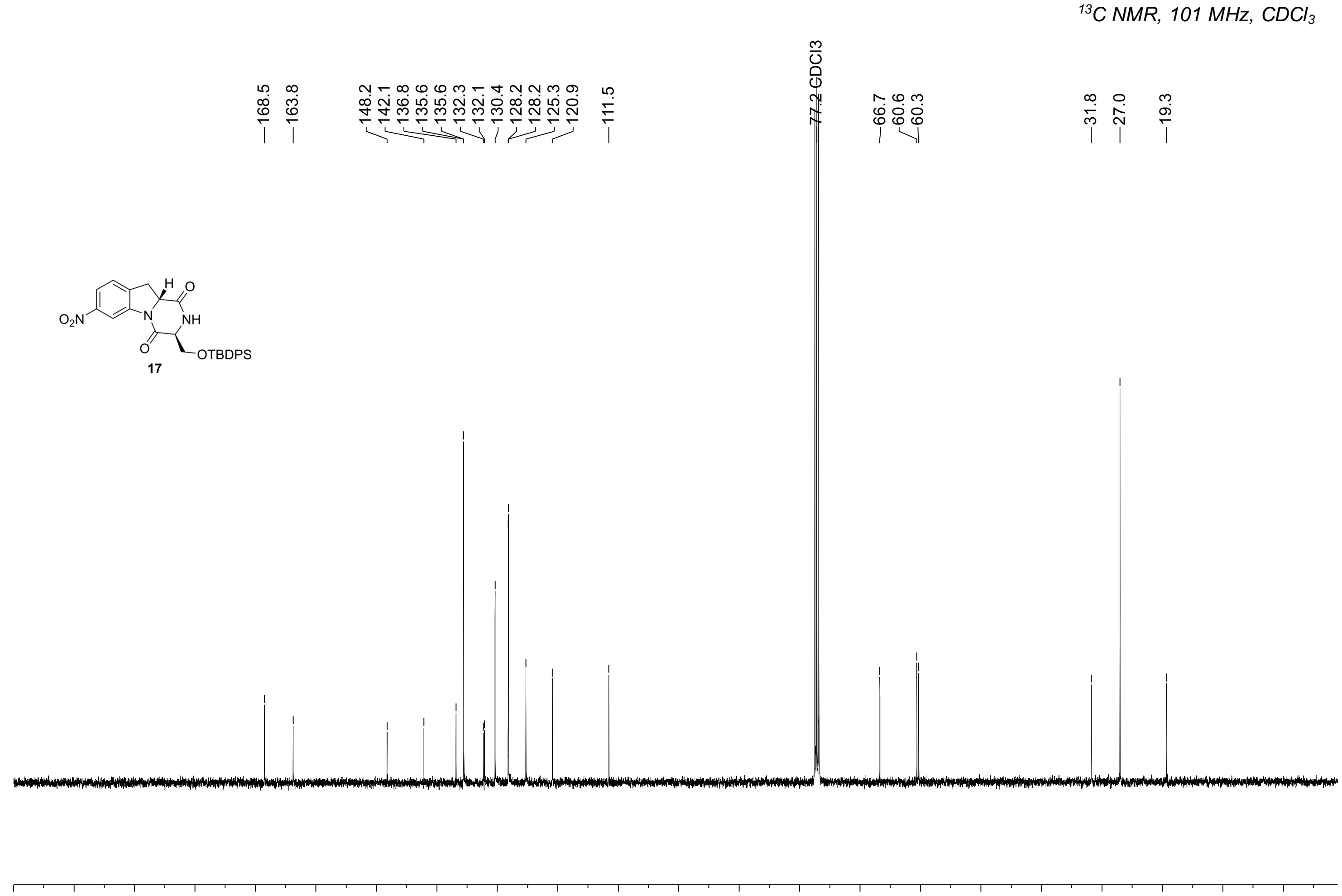
^{13}C NMR, 101 MHz, $CDCl_3$
168.5 163.8 148.2 142.1 136.8 135.6 135.6 132.3 132.1 130.4 128.2 128.2 125.3 120.9 111.5
77.2 CDCl3
66.7 60.6 60.3 31.8 27.0 19.3
O_2N
H
O
NH
N
O
OTBDPS
17
210 200 190 180 170 160 150 140 130 120 110 100 90 80 70 60 50 40 30 20 10 0

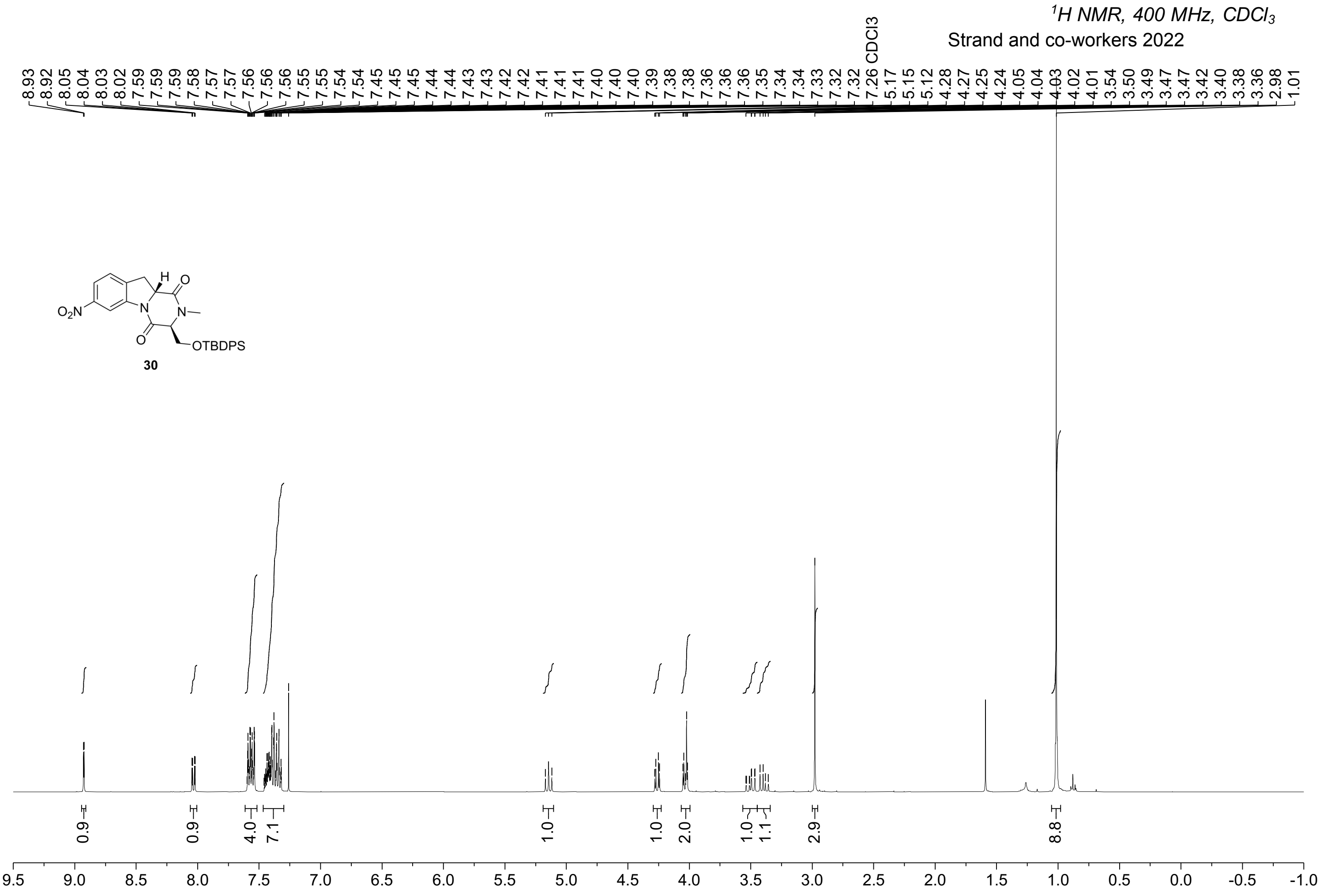

1H NMR, 400 MHz, CDCl3
Strand and co-workers 2022
8.93 8.92 8.05 8.04 8.03 8.02 7.59 7.59 7.59 7.58 7.57 7.57 7.56 7.56 7.56 7.55 7.55 7.54 7.45 7.45 7.45 7.44 7.44 7.43 7.43 7.42 7.42 7.41 7.41 7.41 7.40 7.40 7.39 7.38 7.38 7.36 7.36 7.35 7.34 7.34 7.33 7.32 7.32 7.26 CDCl3 5.17 5.15 5.12 4.28 4.27 4.25 4.24 4.05 4.04 4.03 4.02 4.01 3.54 3.50 3.49 3.47 3.42 3.40 3.38 3.36 2.98 1.01
O2N
N
H
O
N
O
OTBDPS
30
0.9 0.9 4.0 7.1 1.0 1.0 2.0 1.0 1.1 2.9 8.8
9.5 9.0 8.5 8.0 7.5 7.0 6.5 6.0 5.5 5.0 4.5 4.0 3.5 3.0 2.5 2.0 1.5 1.0 0.5 0.0 -0.5 -1.0

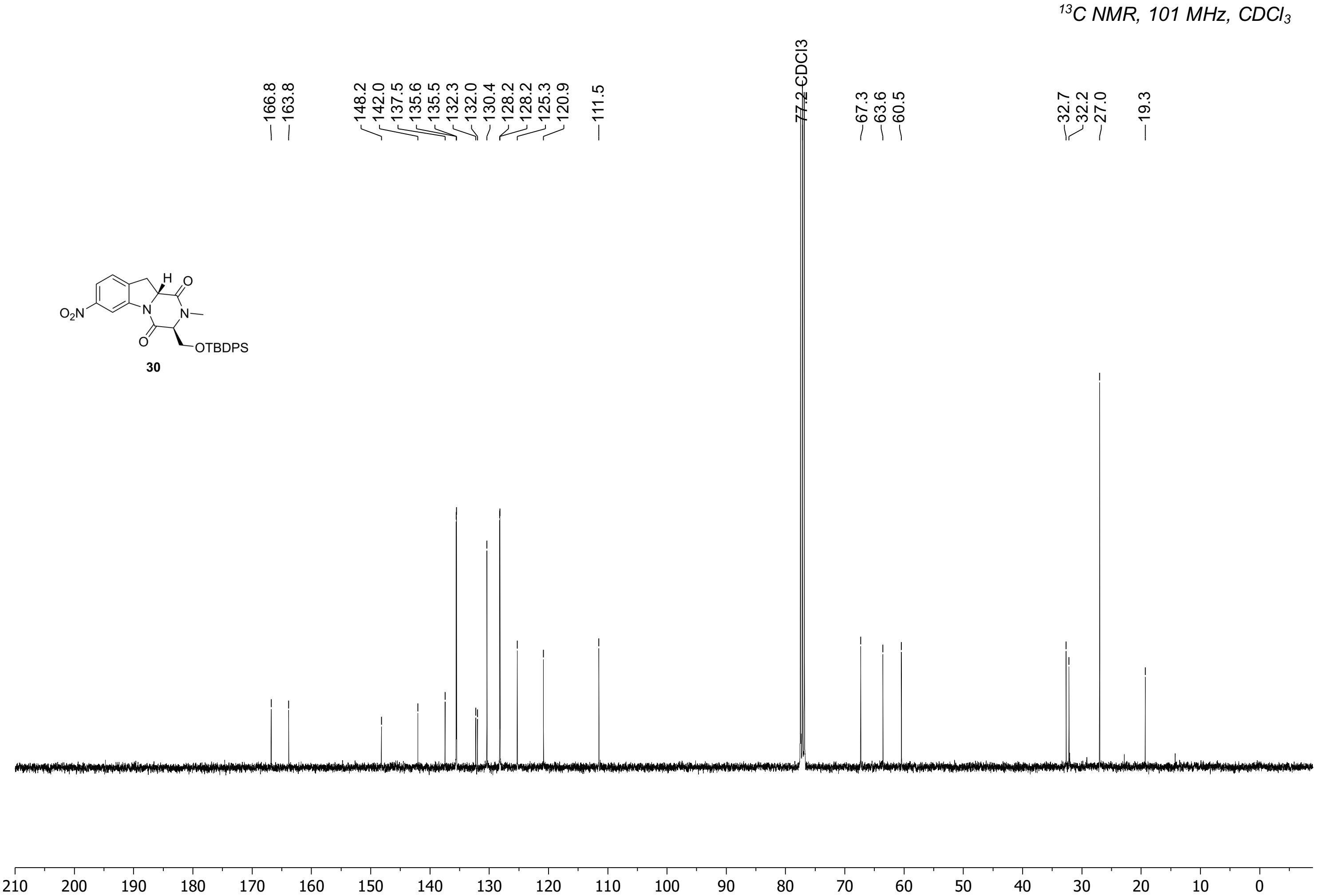

13C NMR, 101 MHz, CDCl3
166.8 163.8 148.2 142.0 137.5 135.6 135.5 132.3 132.0 130.4 128.2 128.2 125.3 120.9 111.5 77.2 CDCl3 67.3 63.6 60.5 32.7 32.2 27.0 19.3
O2N
N
H
O
N
O
OTBDPS
30
210 200 190 180 170 160 150 140 130 120 110 100 90 80 70 60 50 40 30 20 10 0

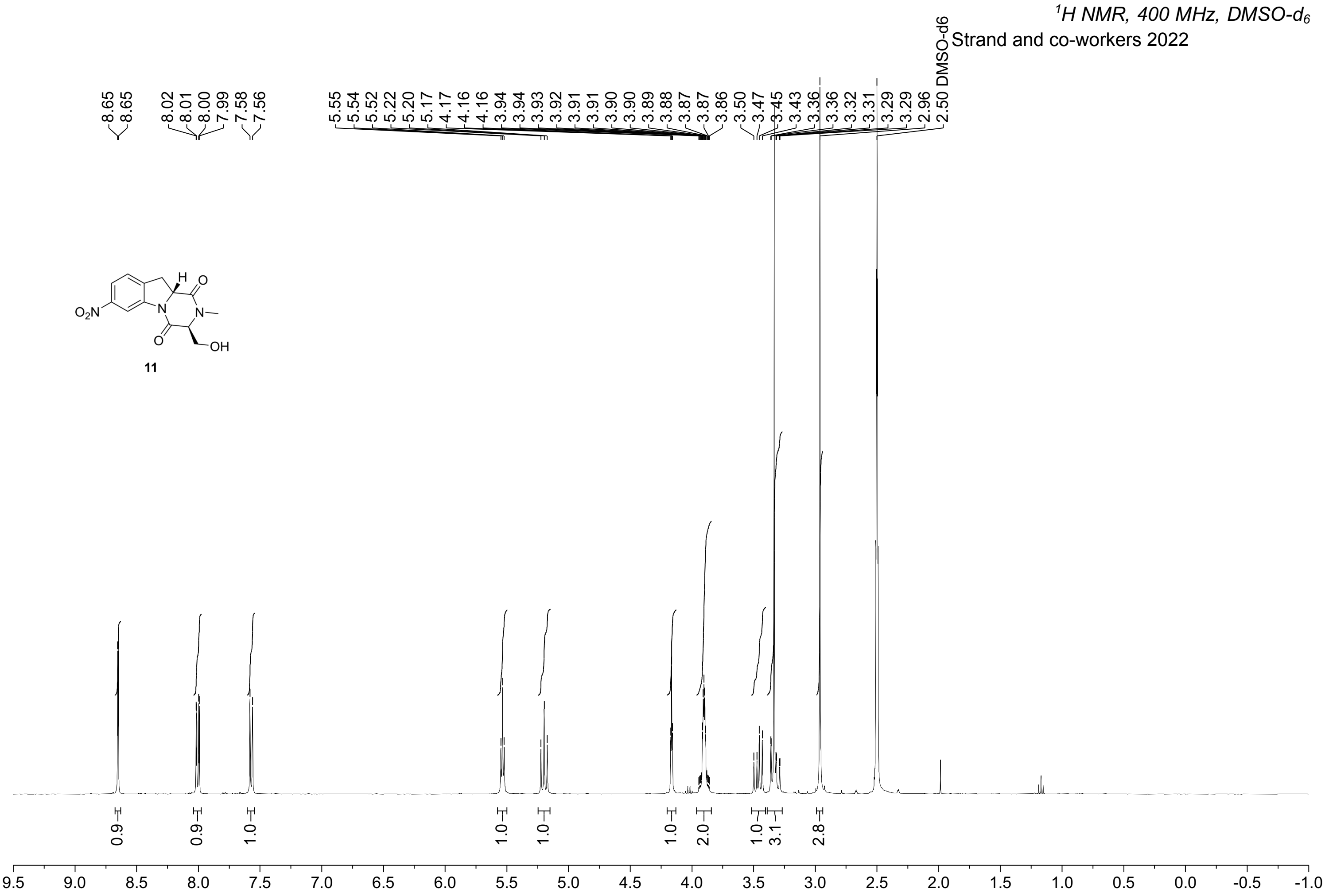
1H NMR, 400 MHz, DMSO-d6
Strand and co-workers 2022
8.65 8.65 8.02 8.01 8.00 7.99 7.58 7.56
5.55 5.54 5.52 5.22 5.20 5.17 4.17 4.16 3.94 3.94 3.93 3.92 3.91 3.91 3.90 3.90 3.89 3.88 3.87 3.87 3.86 3.50 3.47 3.45 3.43 3.36 3.36 3.32 3.31 3.29 3.29 2.96 2.50 DMSO-d6
0.9 0.9 1.0 1.0 1.0 1.0 2.0 1.0 3.1 2.8
O2N
OH
11

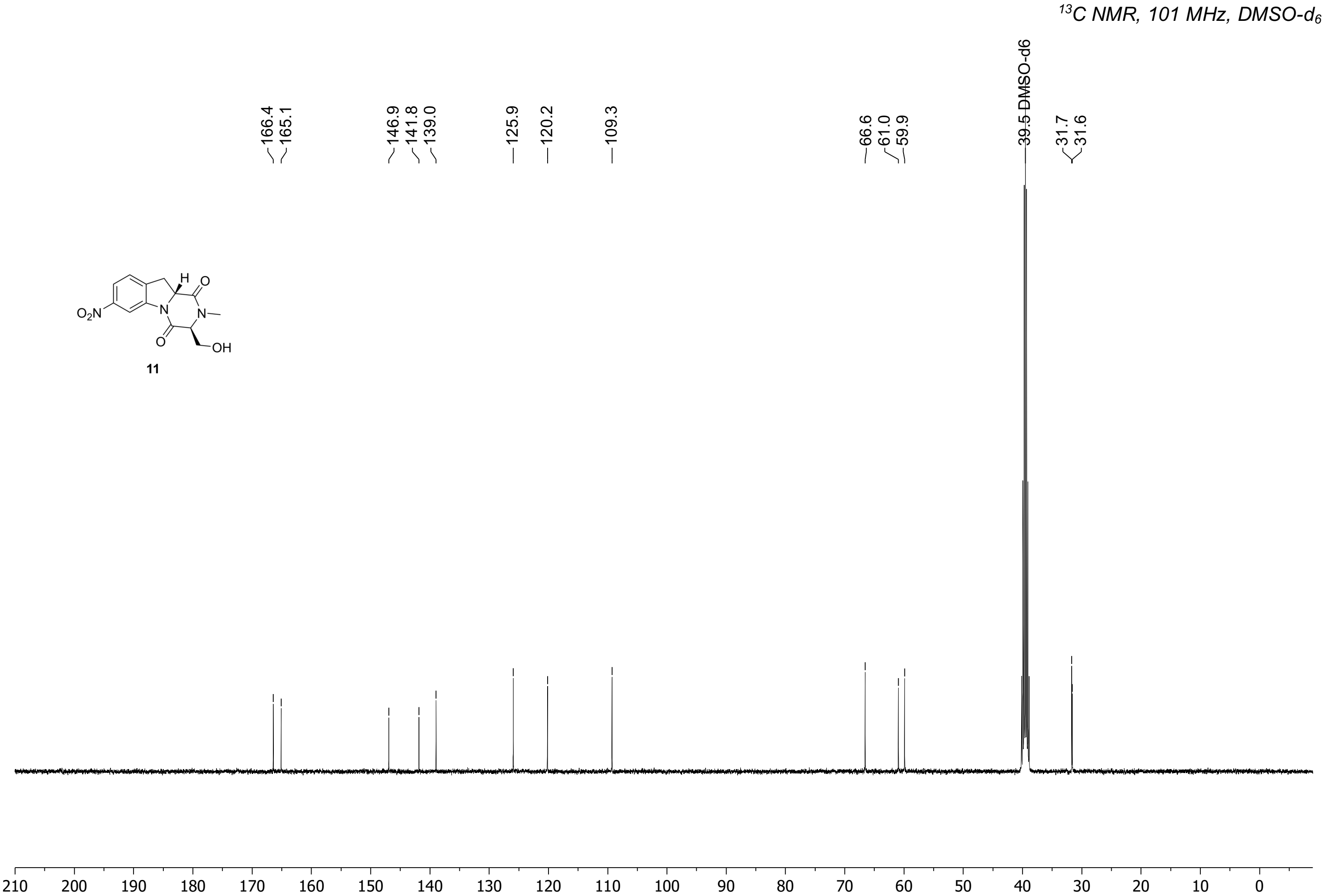
13C NMR, 101 MHz, DMSO-d6
166.4 165.1 146.9 141.8 139.0 125.9 120.2 109.3 66.6 61.0 59.9 39.5 DMSO-d6 31.7 31.6
O2N
OH
11

^{1}H NMR, 400 MHz, $CDCl_3$
Strand and co-workers 2022

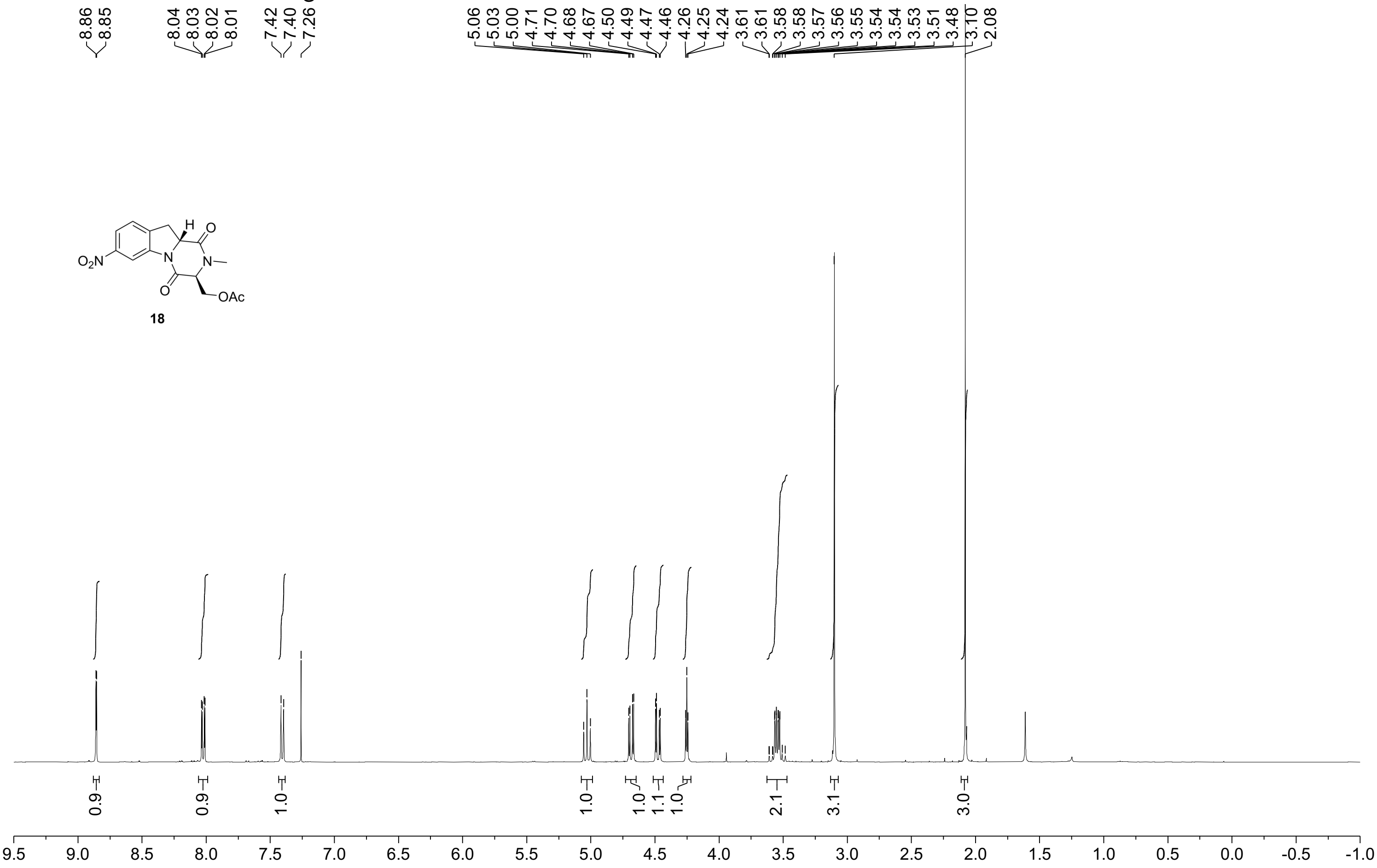

^{13}C NMR, 101 MHz, $CDCl_3$

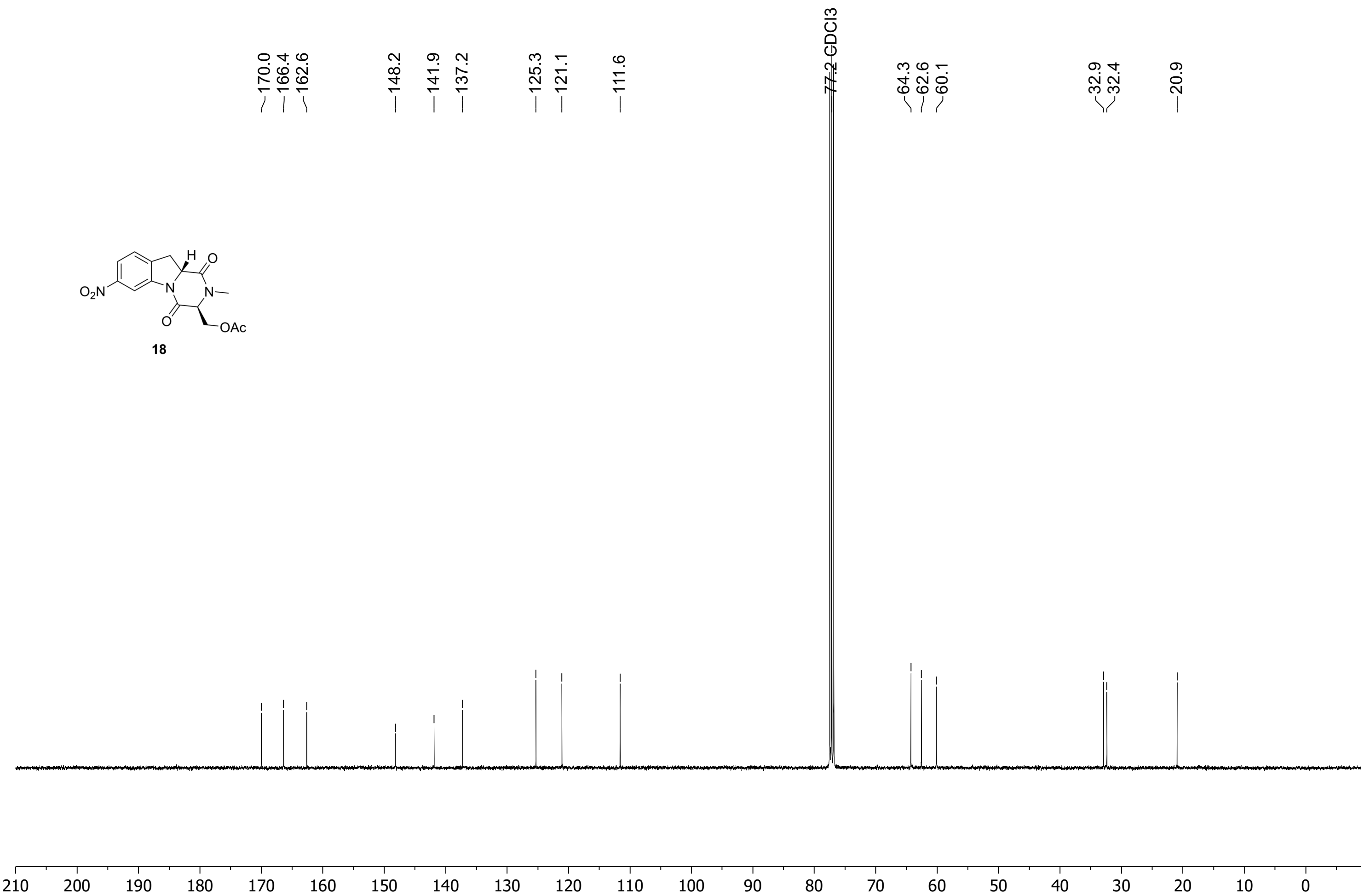

¹H NMR, 400 MHz, CDCl₃
Strand and co-workers 2022

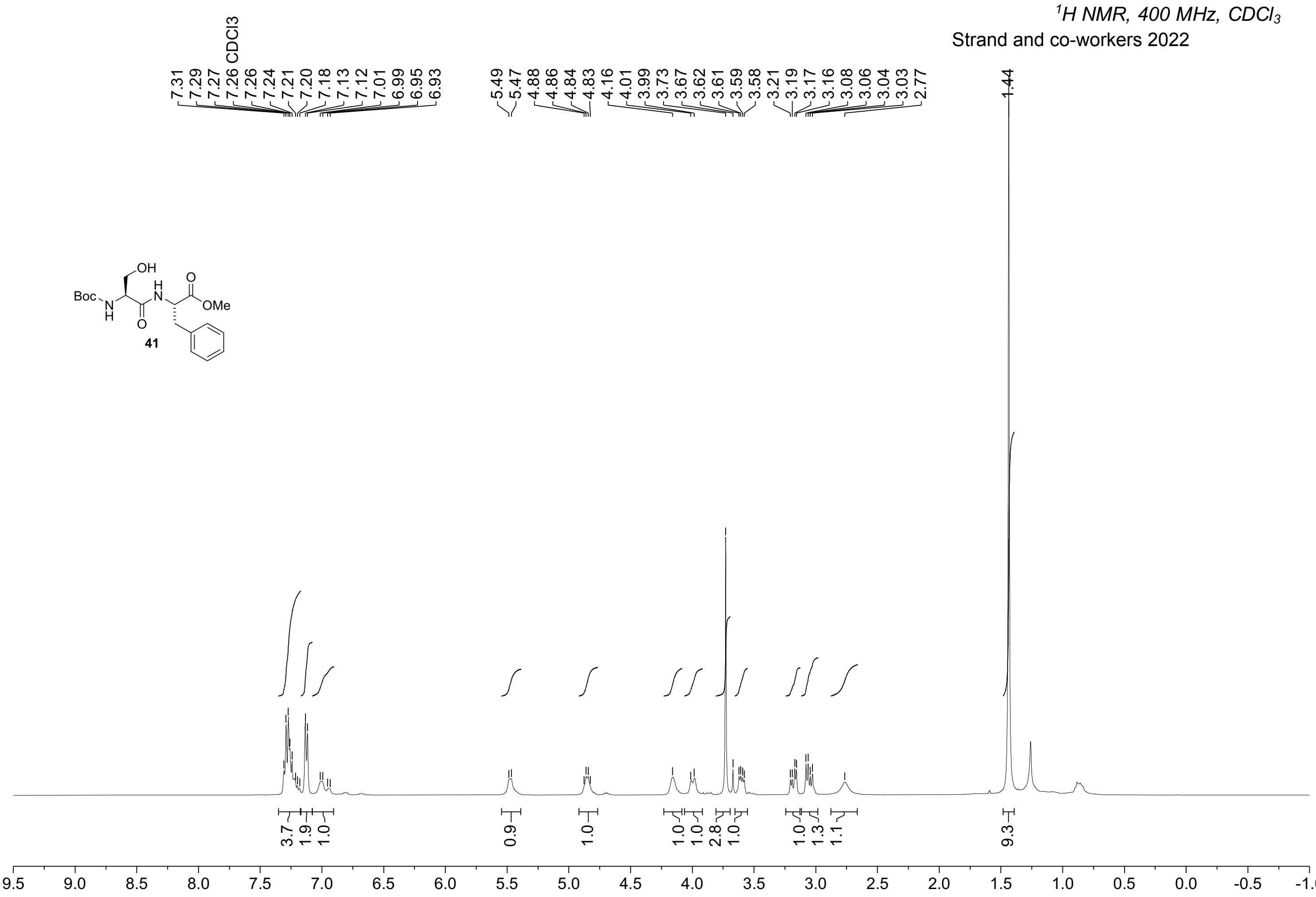

¹³C NMR, 101 MHz, CDCl₃

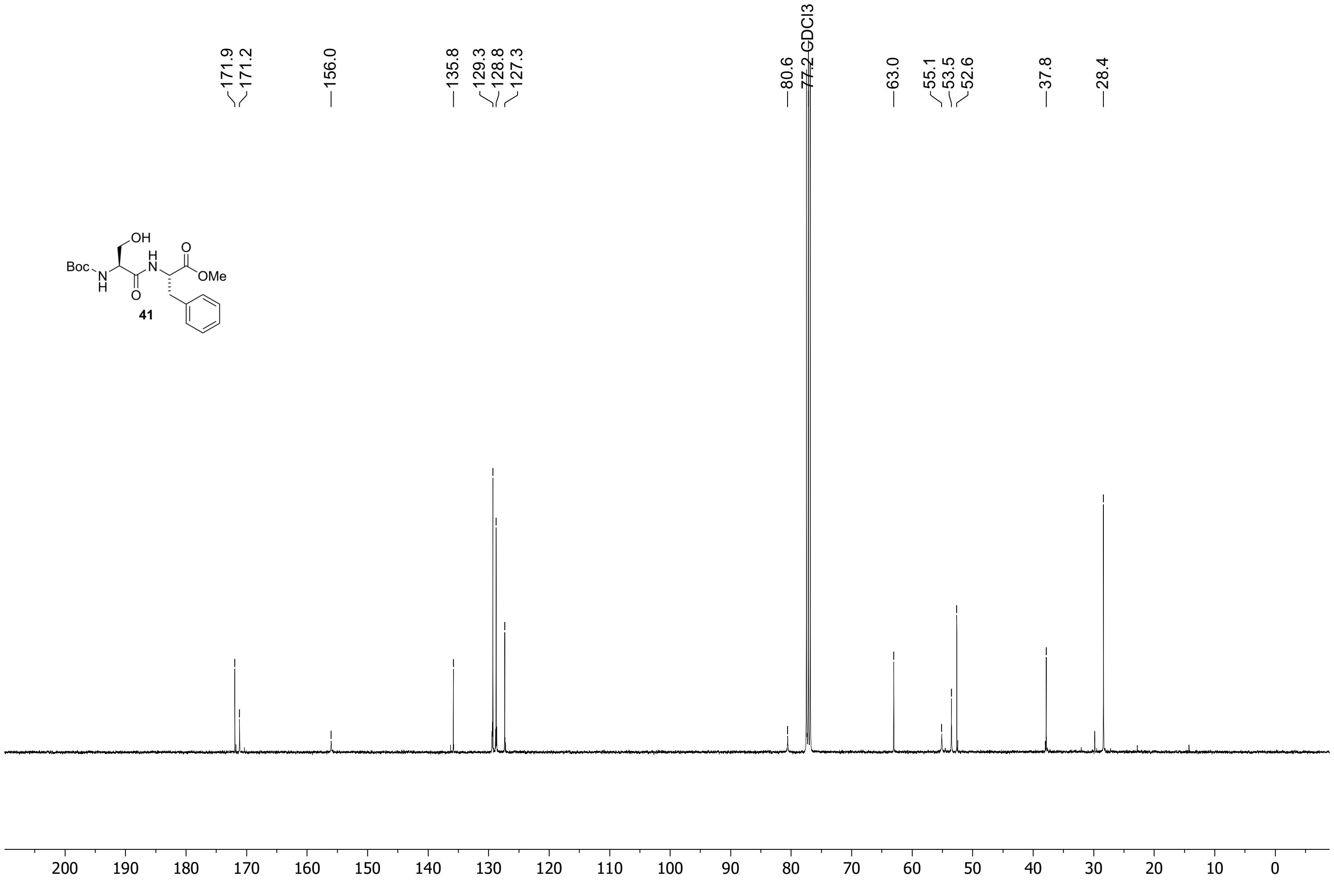

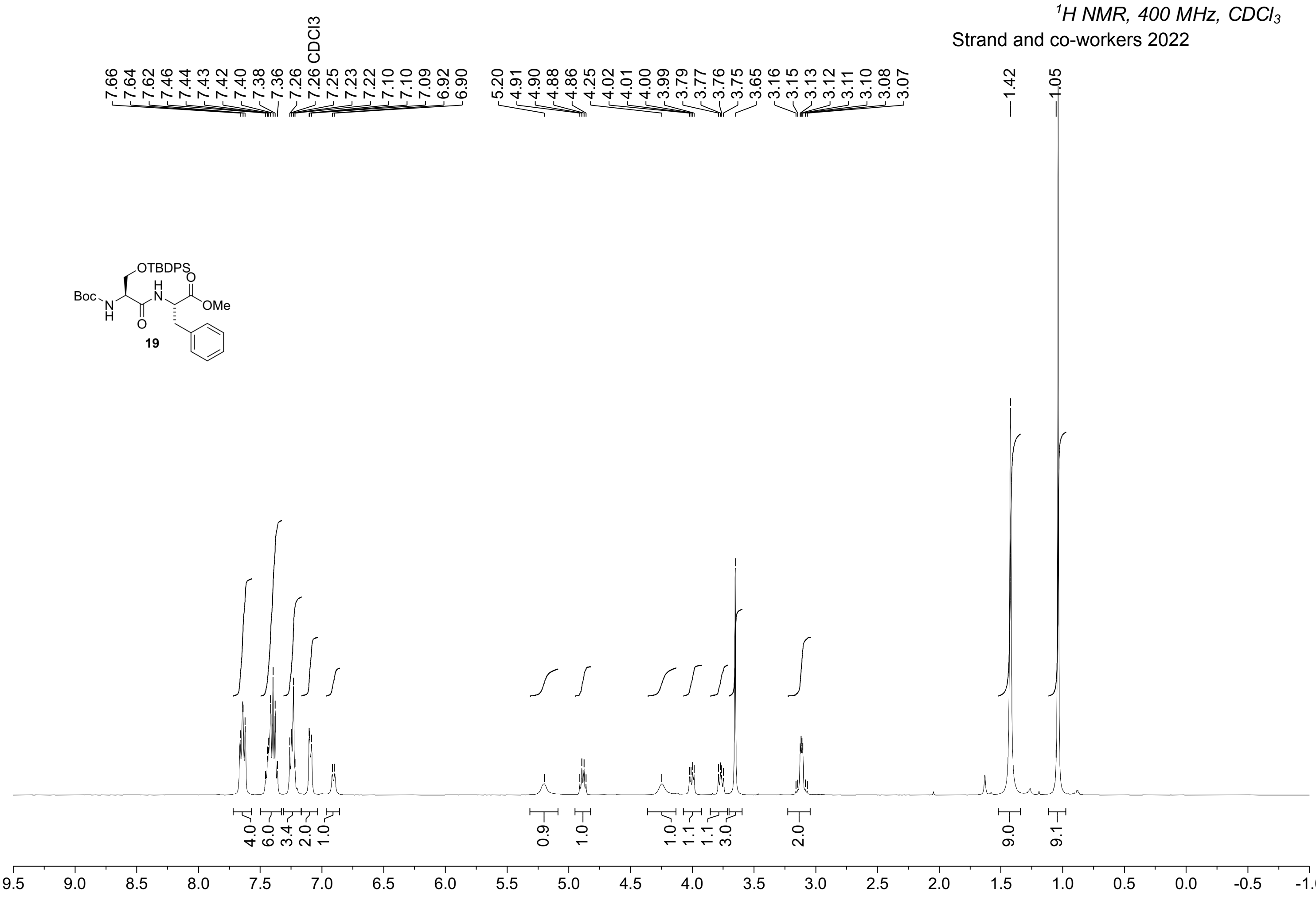
^1H NMR, 400 MHz, $CDCl_3$
Strand and co-workers 2022
7.66 7.64 7.62 7.46 7.44 7.43 7.42 7.40 7.38 7.36 7.26 CDCl3 7.26 7.25 7.23 7.22 7.10 7.09 6.92 6.90 5.20 4.91 4.90 4.88 4.86 4.25 4.02 4.01 4.00 3.99 3.79 3.77 3.76 3.75 3.65 3.16 3.15 3.13 3.12 3.11 3.10 3.08 3.07 1.42 1.05
OTBDPS
Boc
N
H
O
OMe
19
4.0 6.0 3.4 2.0 1.0 0.9 1.0 1.0 1.1 1.1 3.0 2.0 9.0 9.1
9.5 9.0 8.5 8.0 7.5 7.0 6.5 6.0 5.5 5.0 4.5 4.0 3.5 3.0 2.5 2.0 1.5 1.0 0.5 0.0 -0.5 -1.0

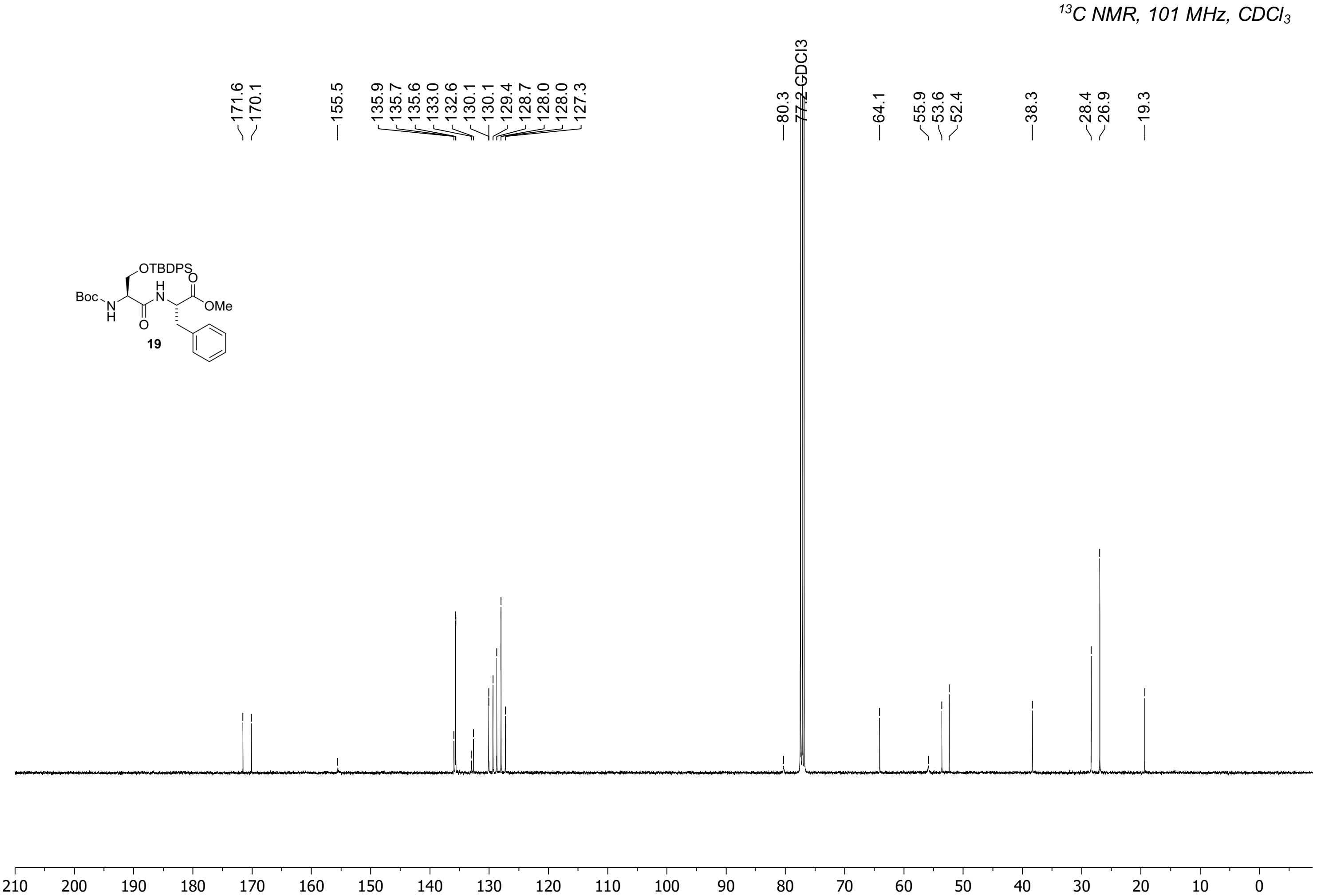
^{13}C NMR, 101 MHz, $CDCl_3$
171.6 170.1 155.5 135.9 135.7 135.6 133.0 132.6 130.1 130.1 129.4 128.7 128.0 128.0 127.3 80.3 77.2 CDCl3 64.1 55.9 53.6 52.4 38.3 28.4 26.9 19.3
OTBDPS
Boc
N
H
O
OMe
19
210 200 190 180 170 160 150 140 130 120 110 100 90 80 70 60 50 40 30 20 10 0

¹H NMR, 400 MHz, CDCl₃
Strand and co-workers 2022

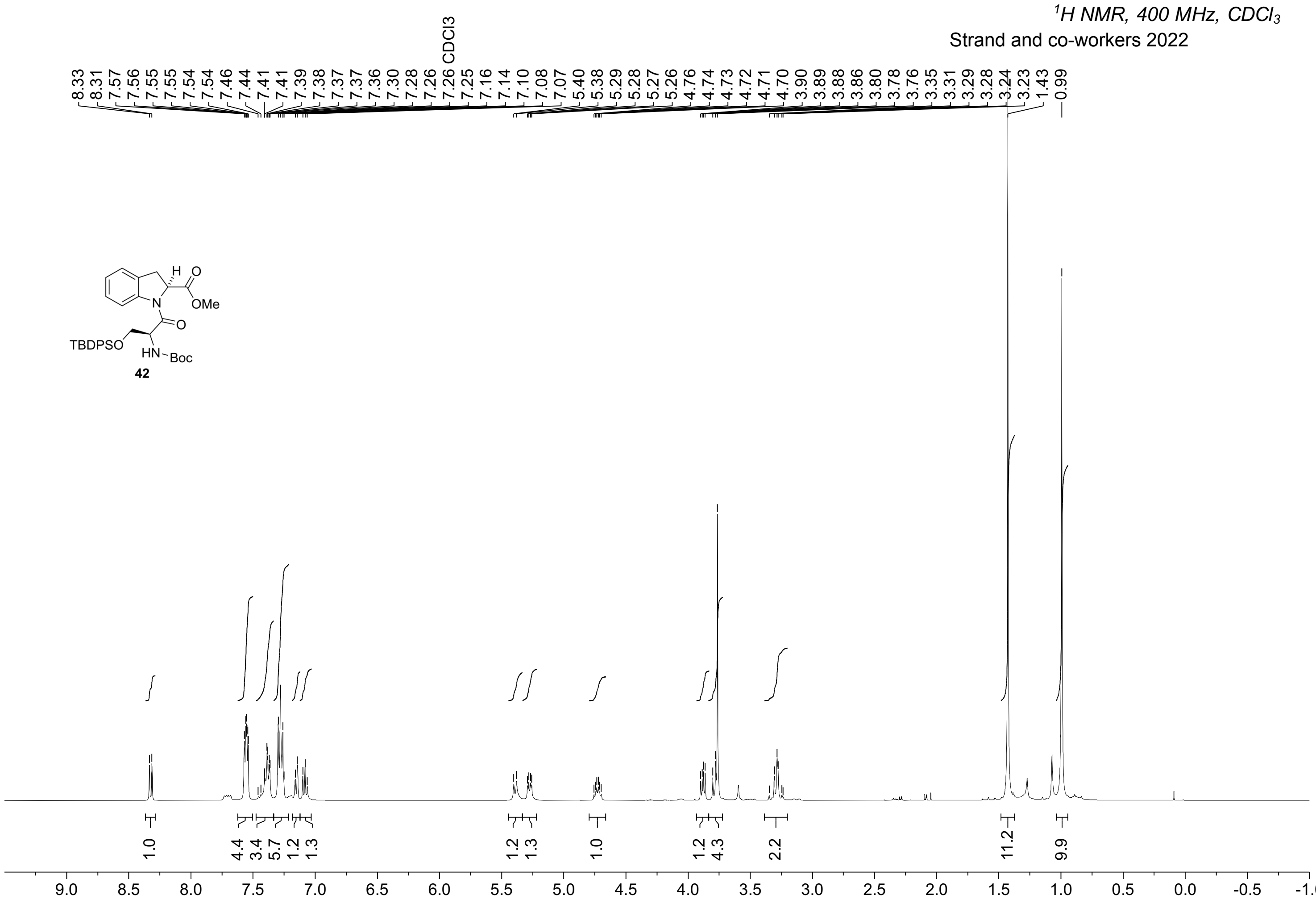

¹³C NMR, 101 MHz, CDCl₃

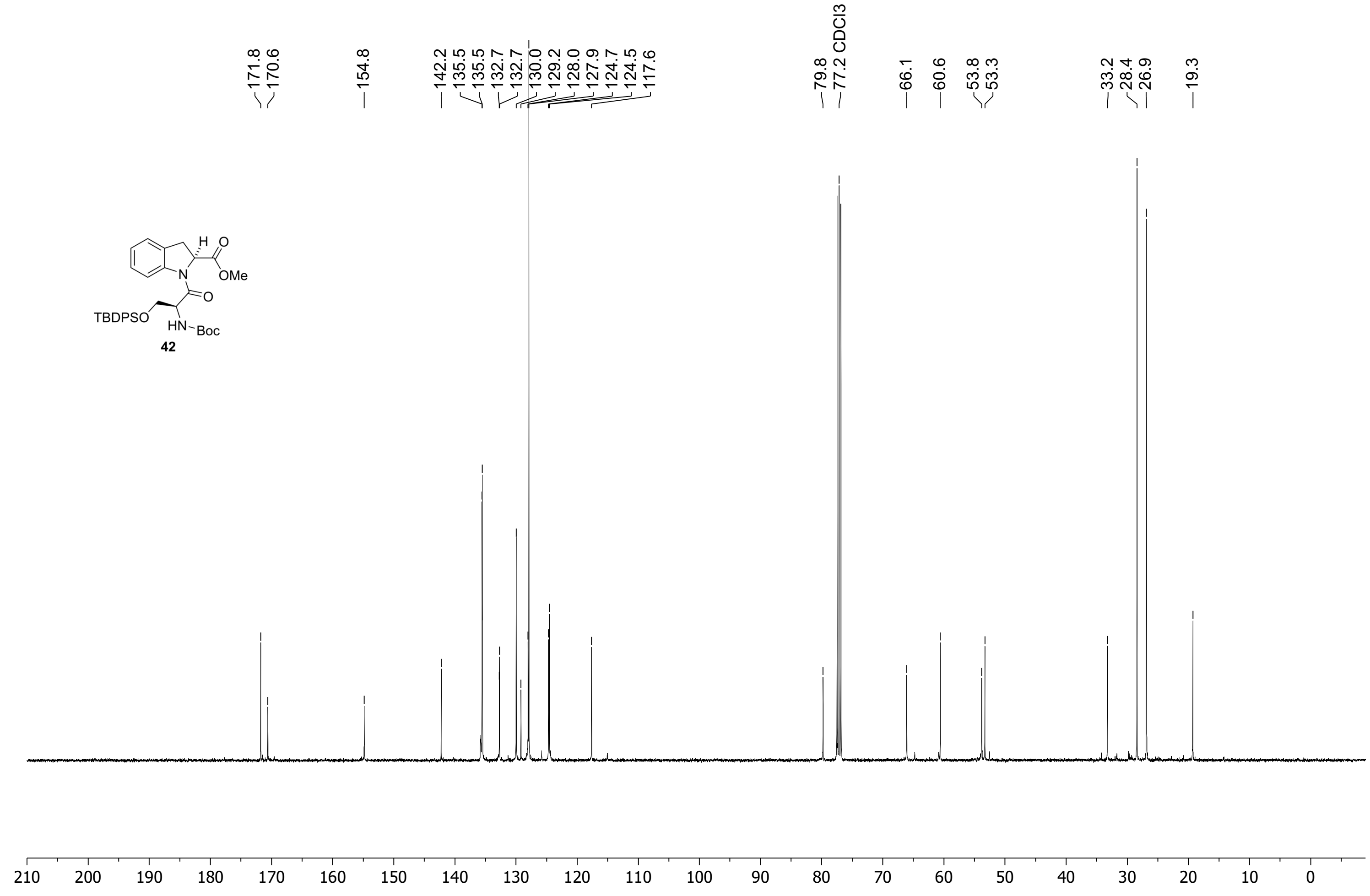

^{1}H NMR, 400 MHz, $CDCl_3$

Strand and co-workers 2022

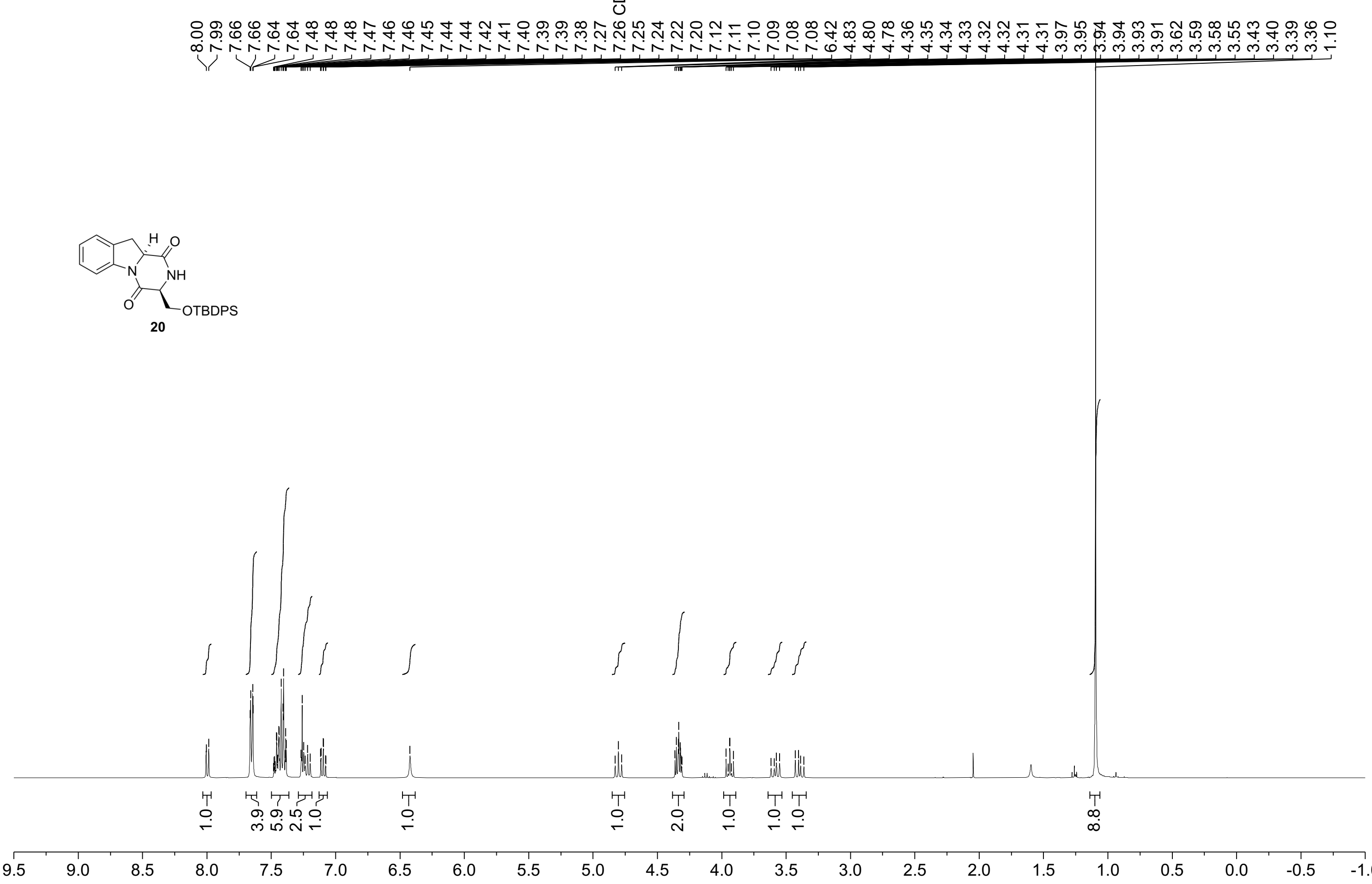

^{13}C NMR, 101 MHz, $CDCl_3$

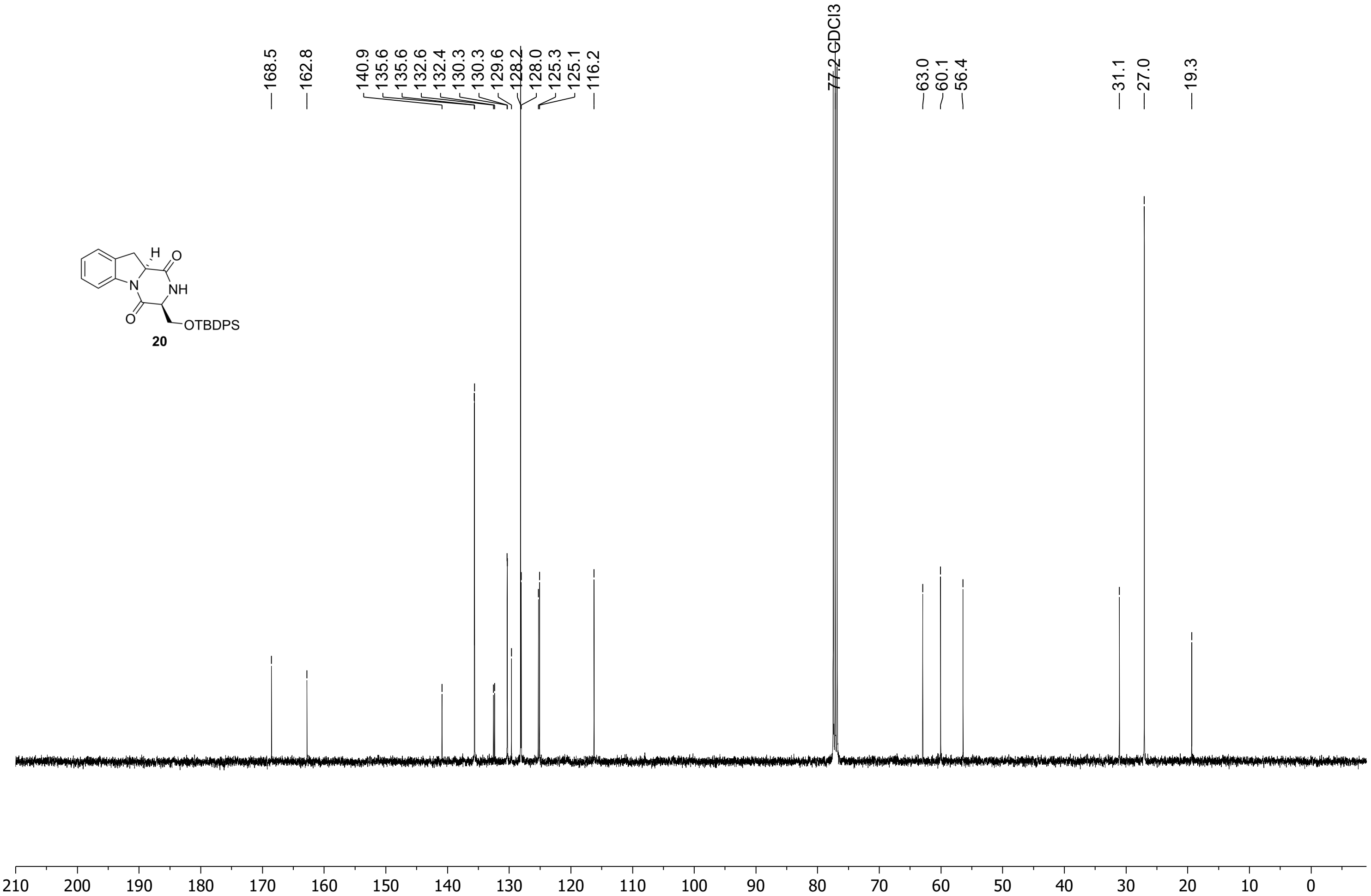

^{1}H NMR, 400 MHz, $CDCl_3$
Strand and co-workers 2022

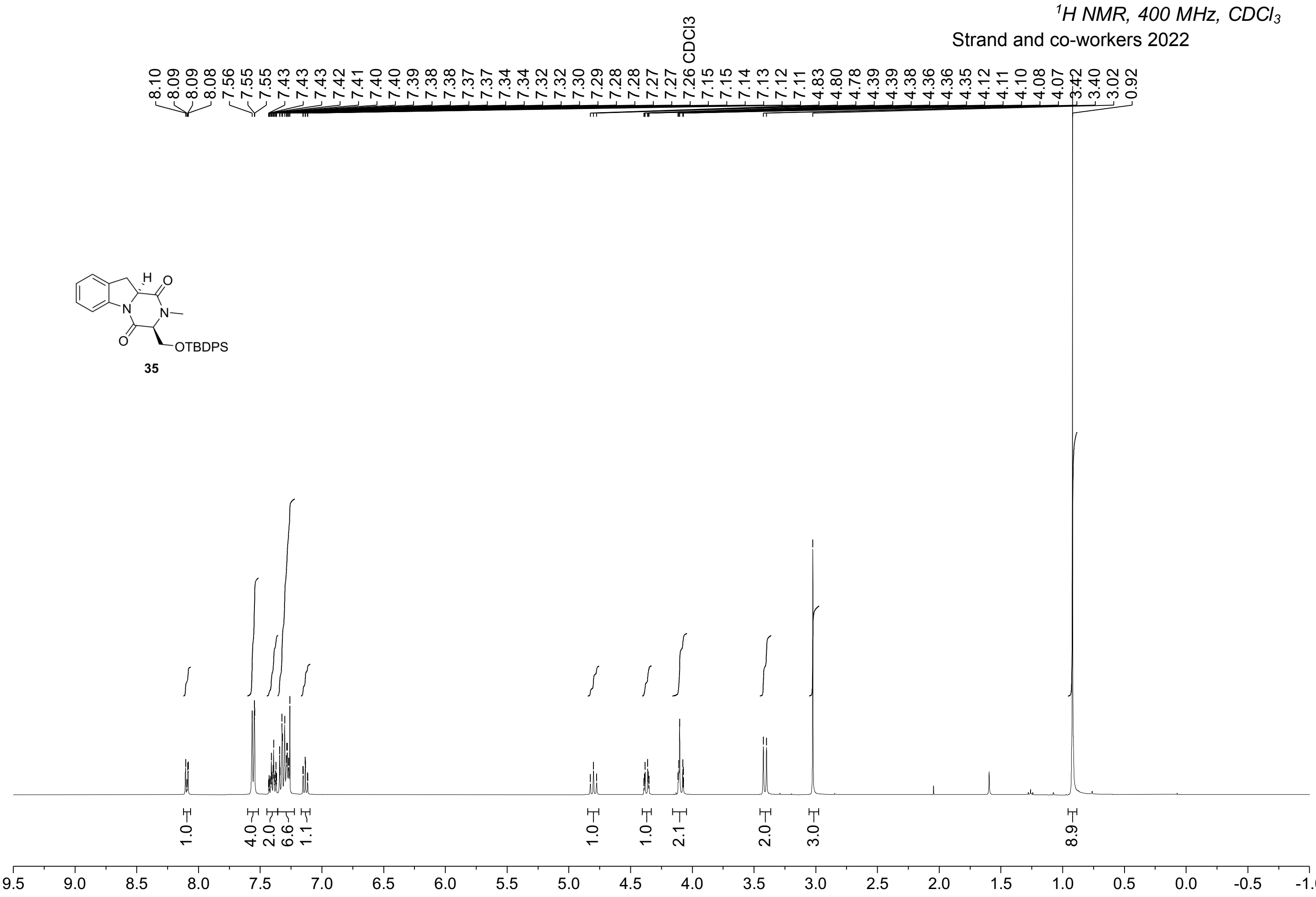

^{13}C NMR, 101 MHz, $CDCl_3$

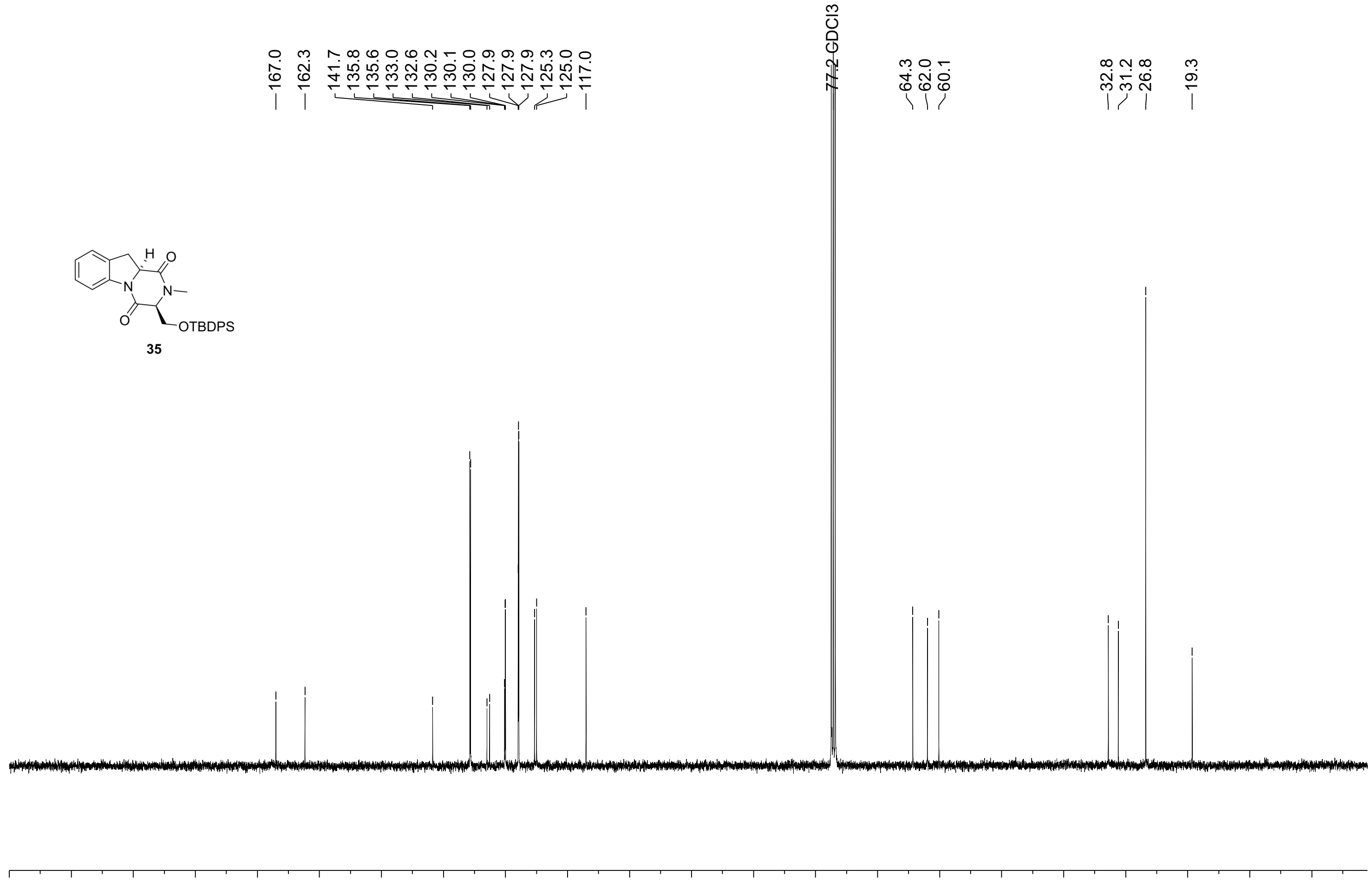

^{1}H *NMR, 400 MHz,* $CDCl_3$
Strand and co-workers 2022

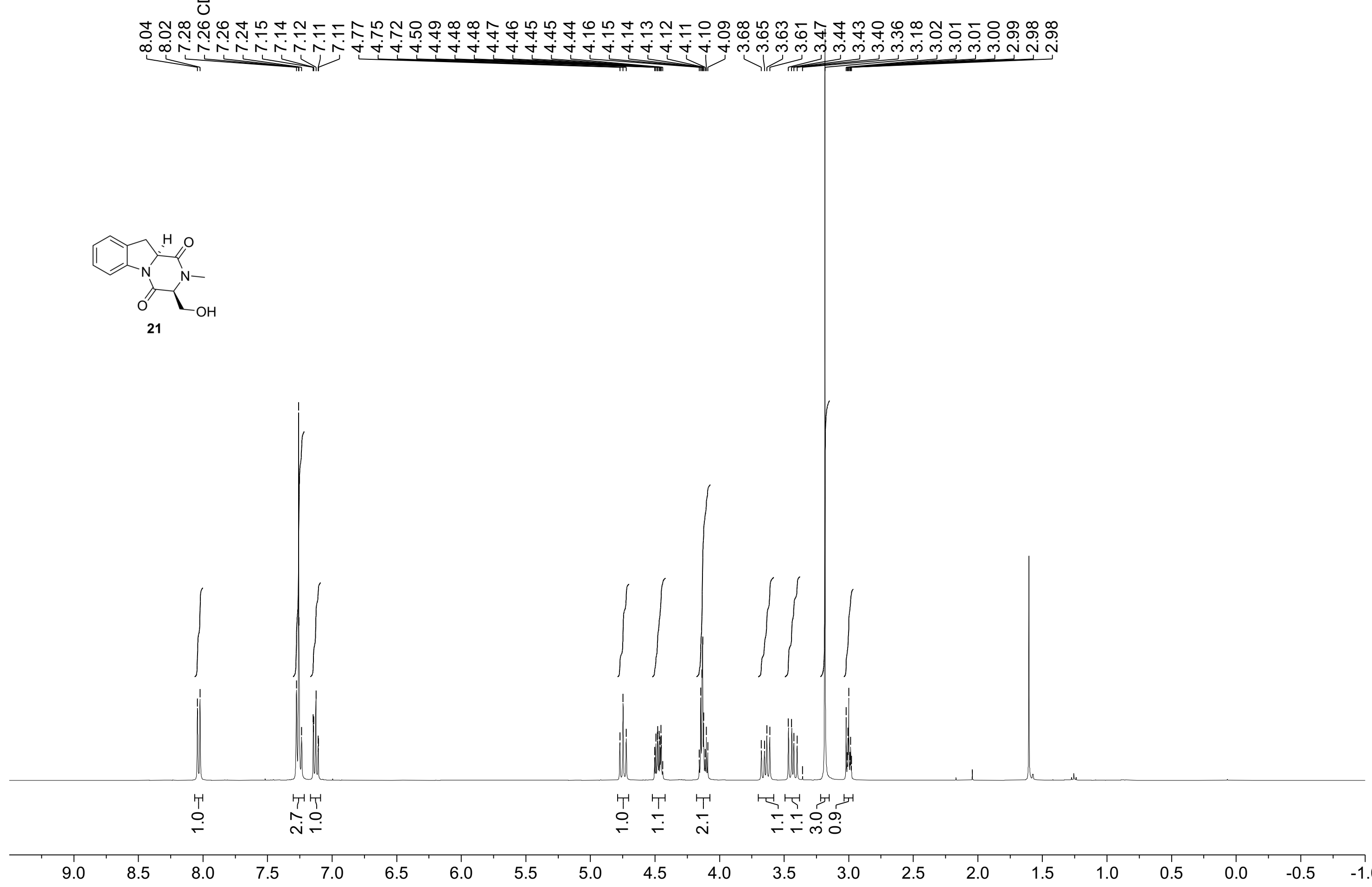

^{13}C *NMR, 101 MHz,* $CDCl_3$

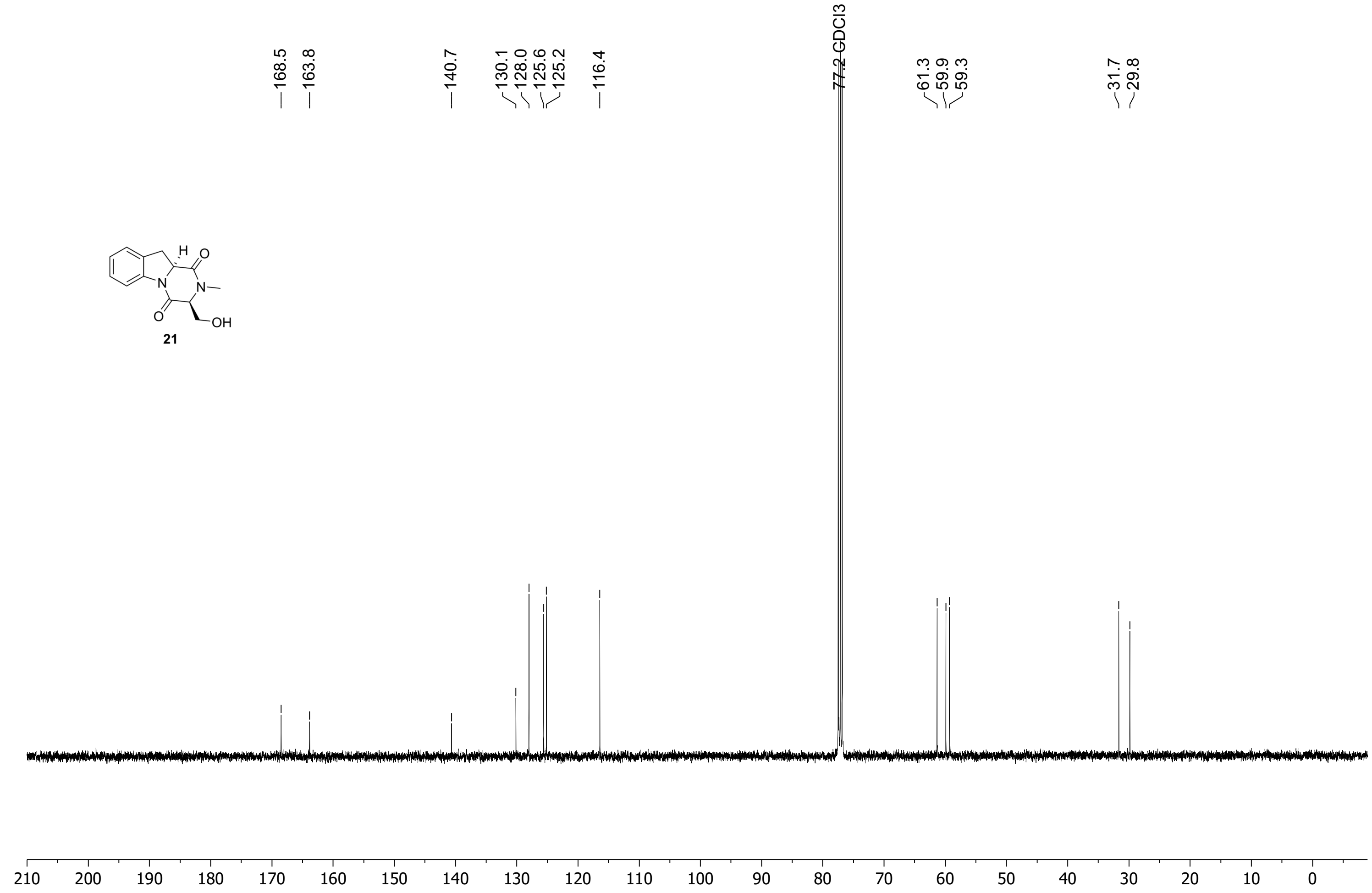

^{1}H NMR, 400 MHz, $CDCl_3$

Strand and co-workers 2022

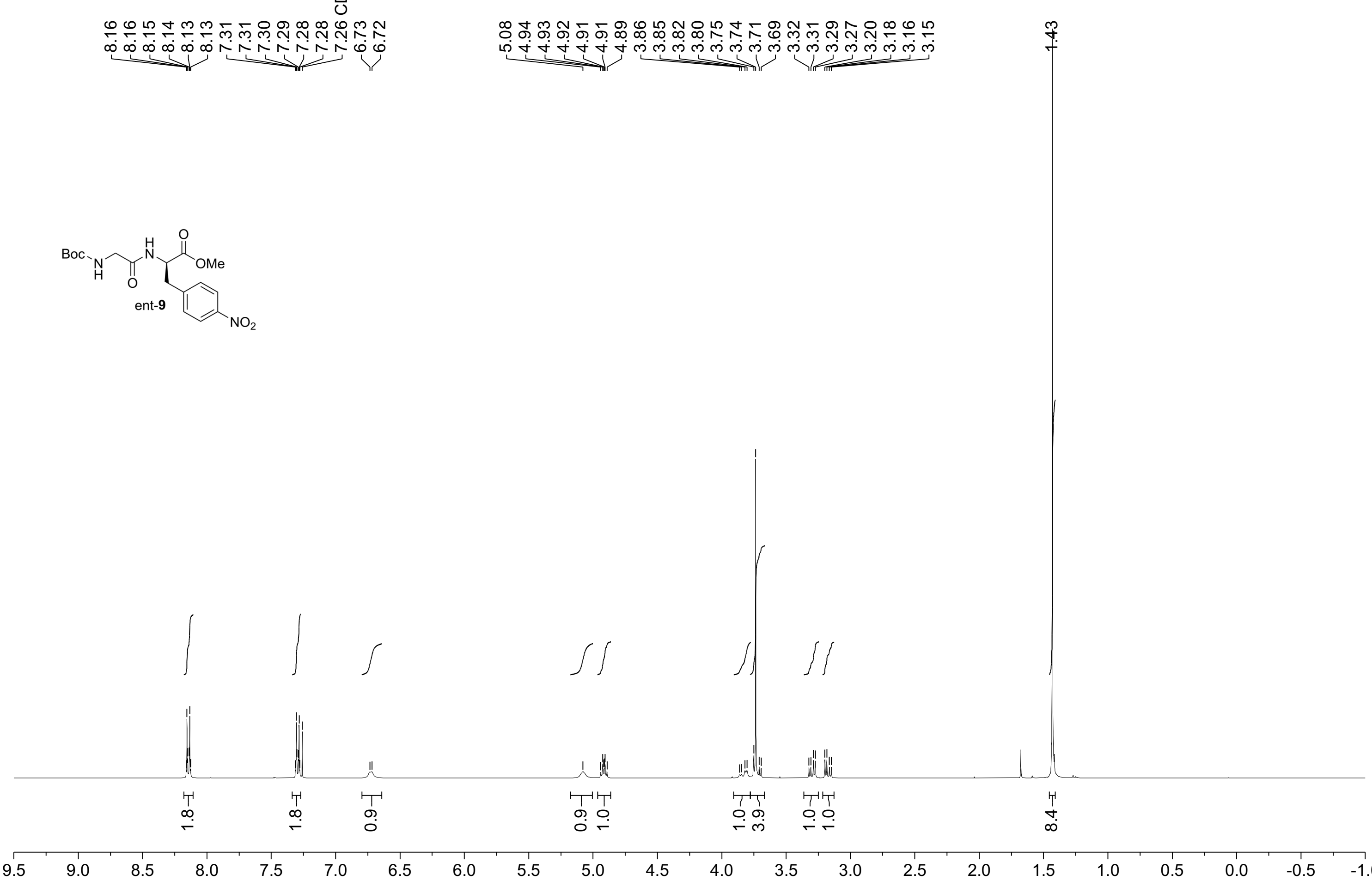

^{13}C NMR, 101 MHz, $CDCl_3$

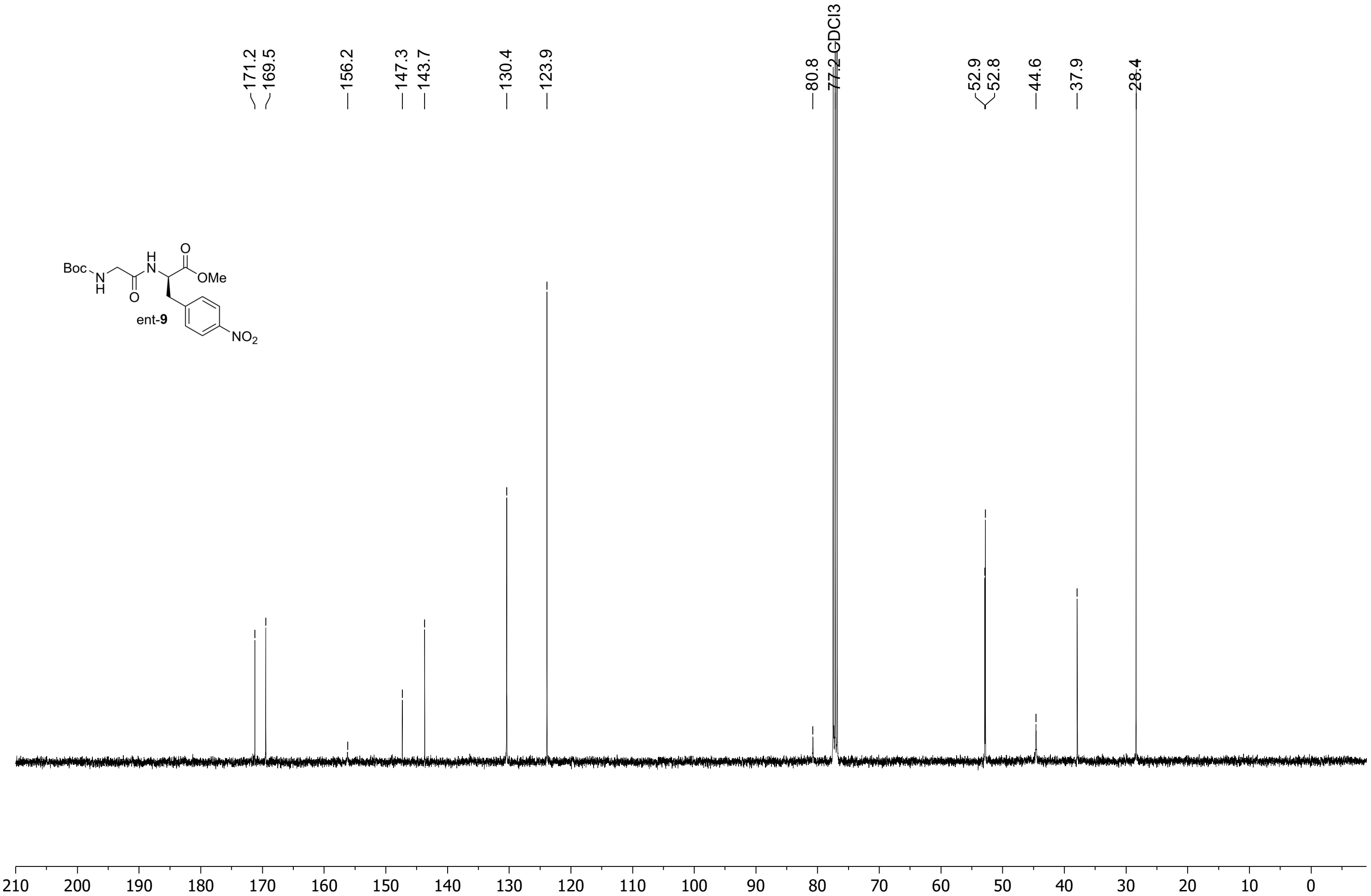

^{1}H NMR, 400 MHz, CDCl$_3$

Strand and co-workers 2022

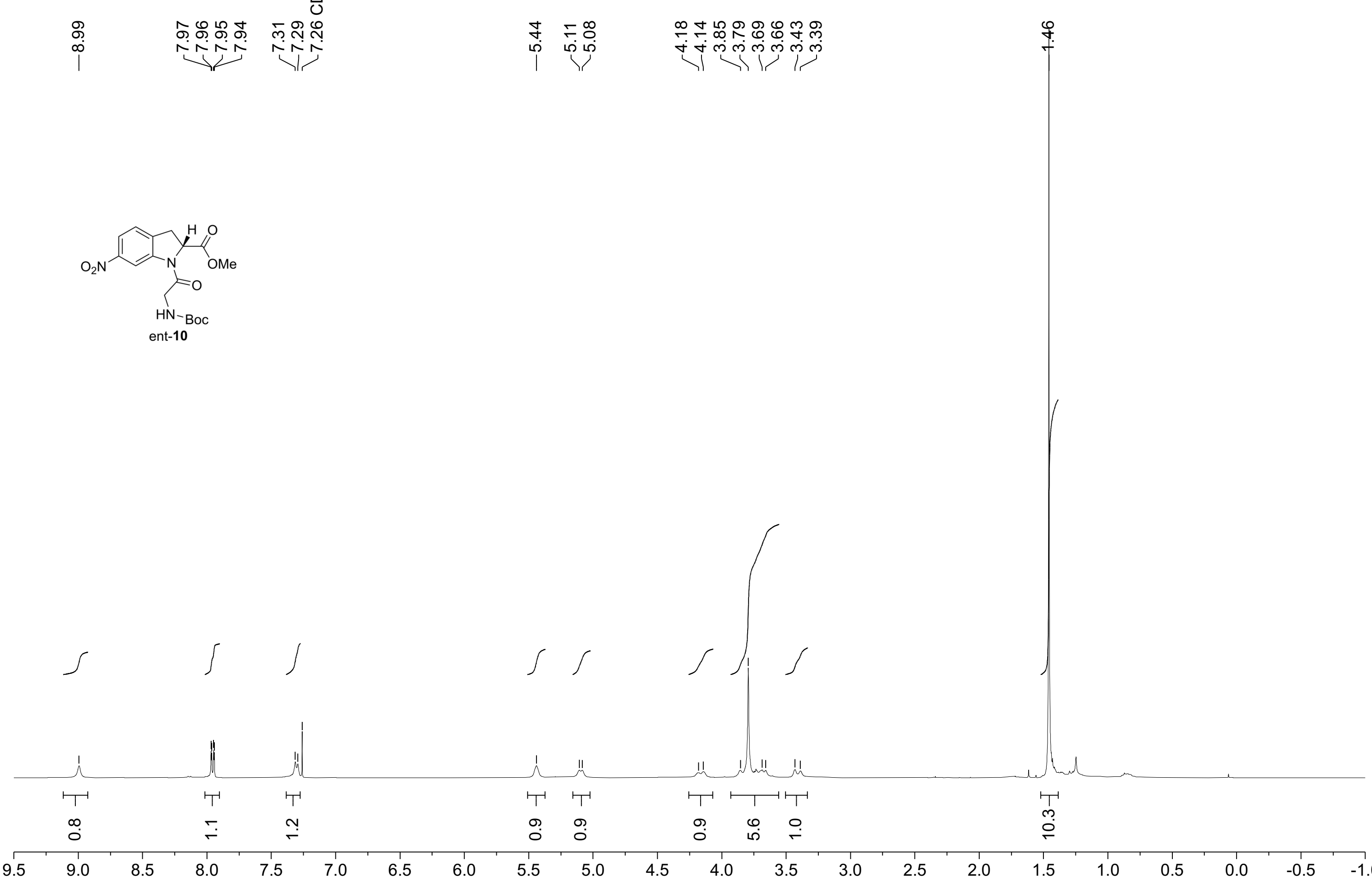

^{13}C NMR, 101 MHz, CDCl$_3$

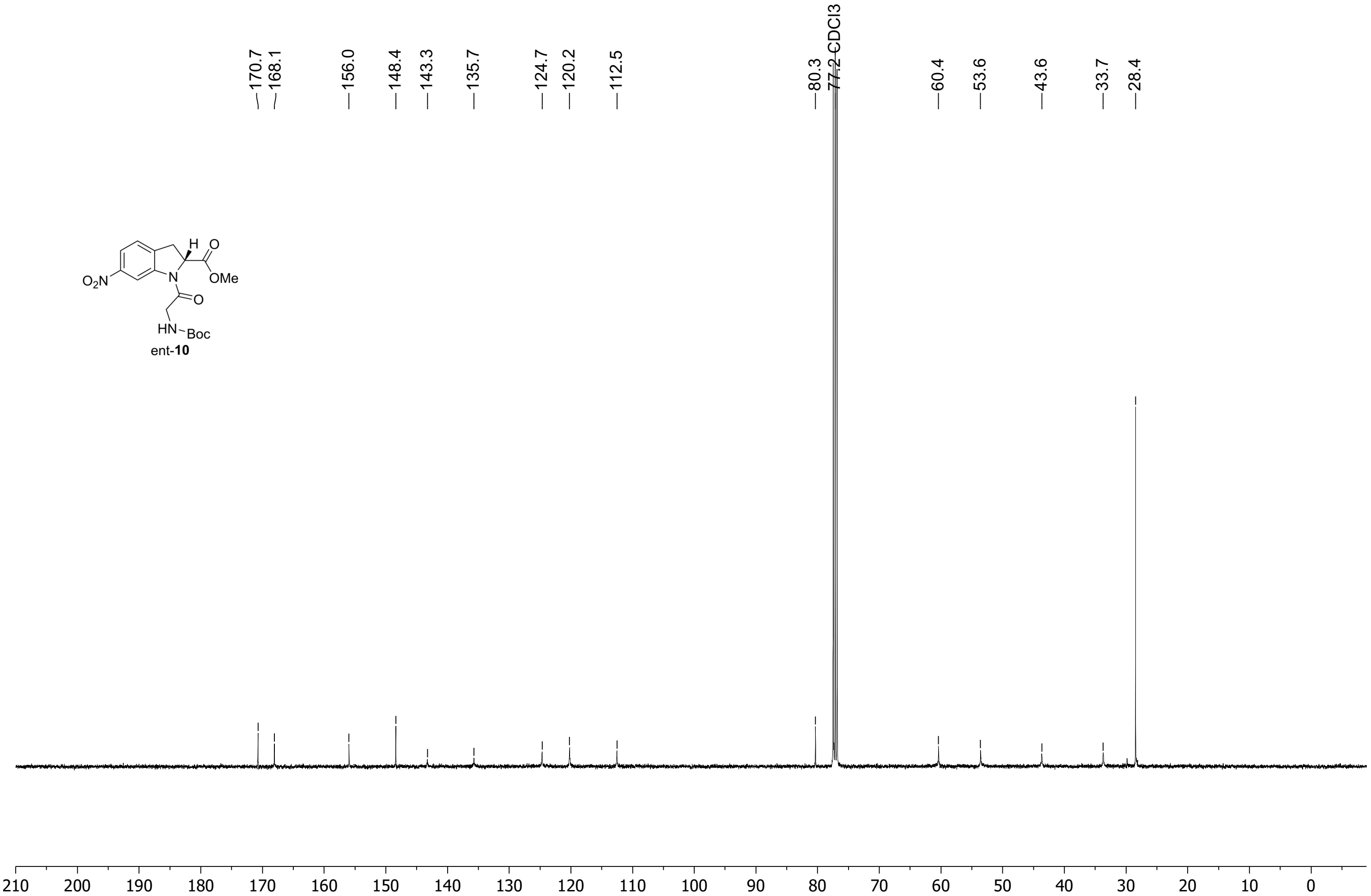

^{1}H NMR, 400 MHz, DMSO-d_6
Strand and co-workers 2022

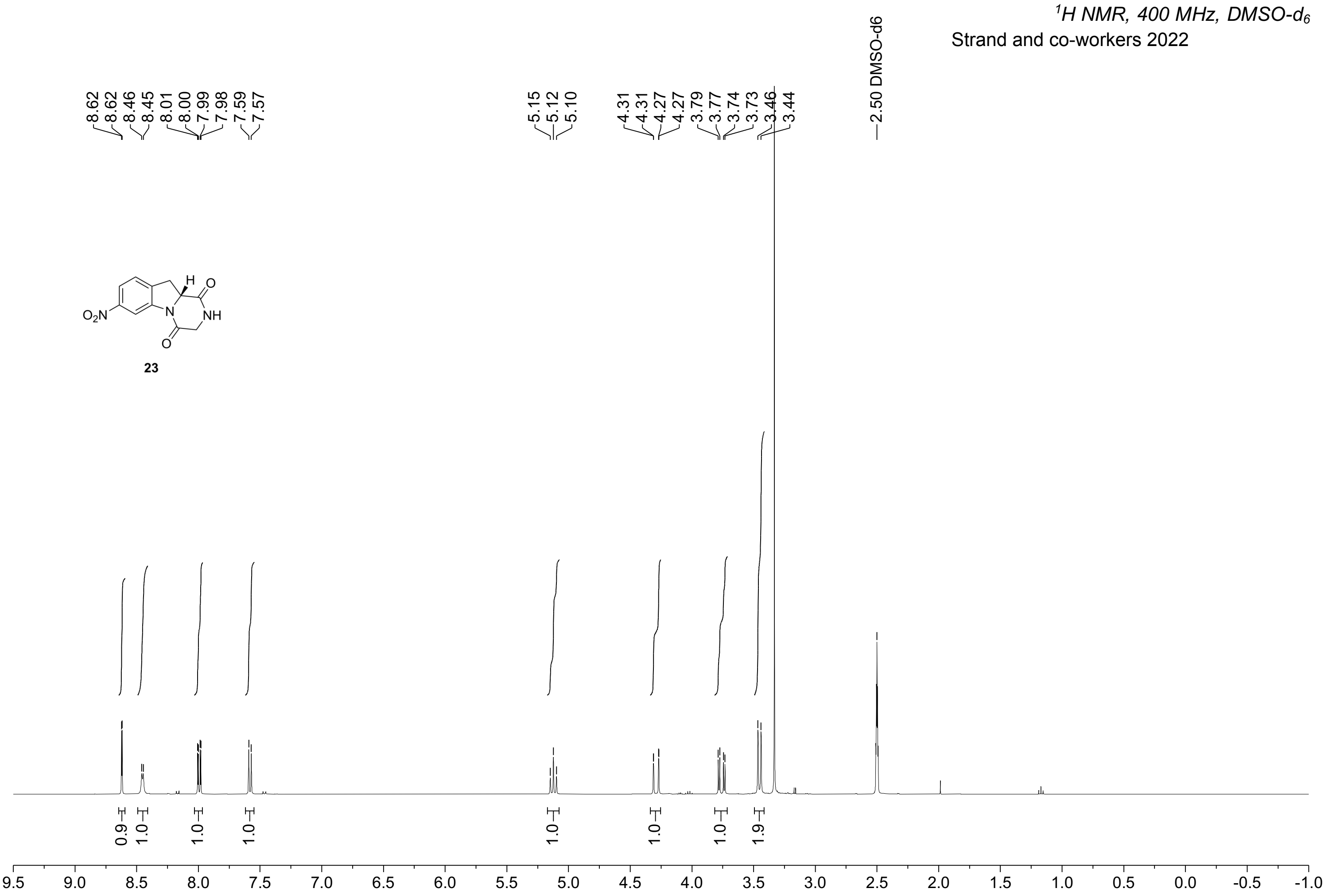

^{13}C NMR, 101 MHz, DMSO-d_6

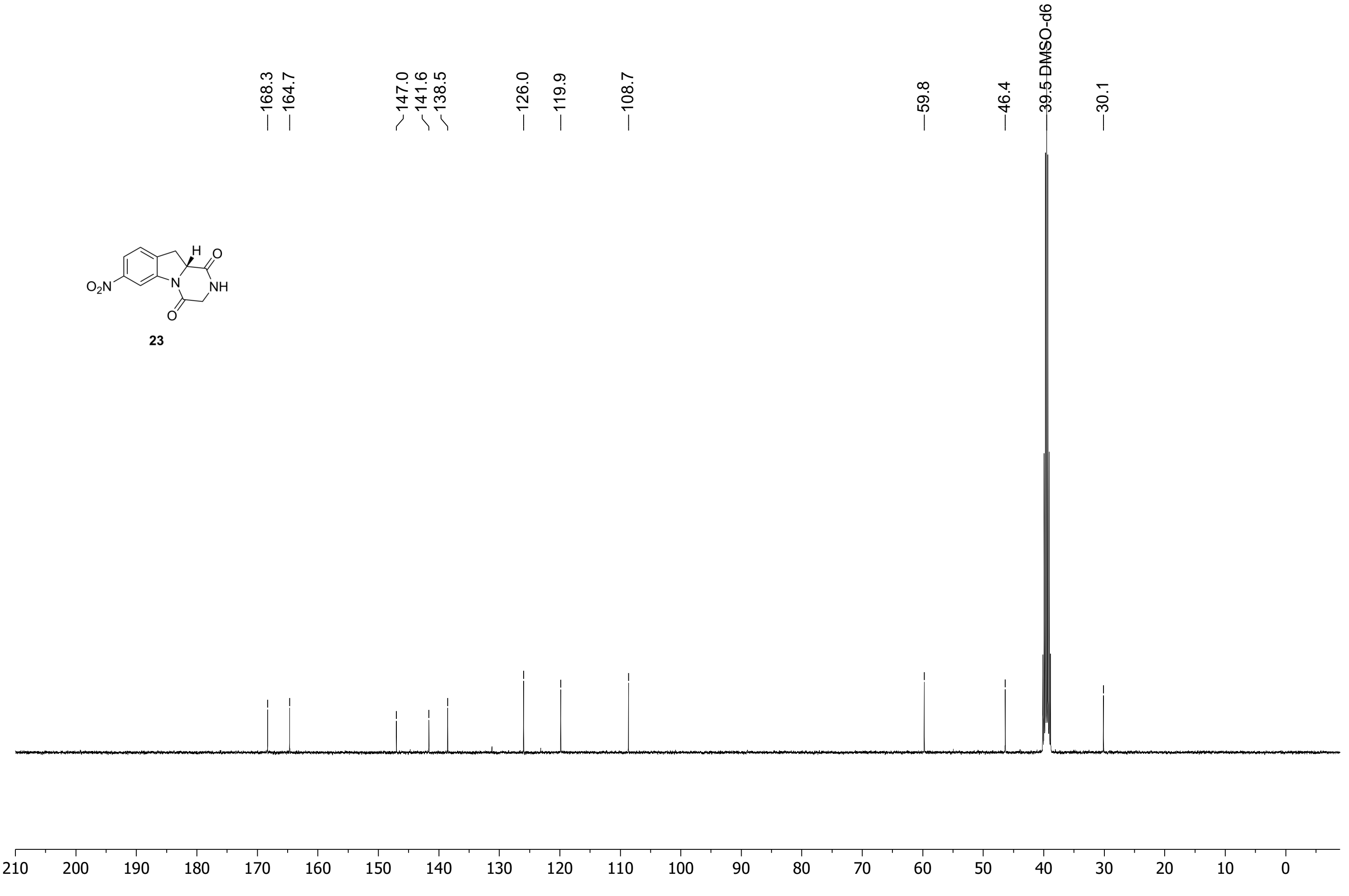

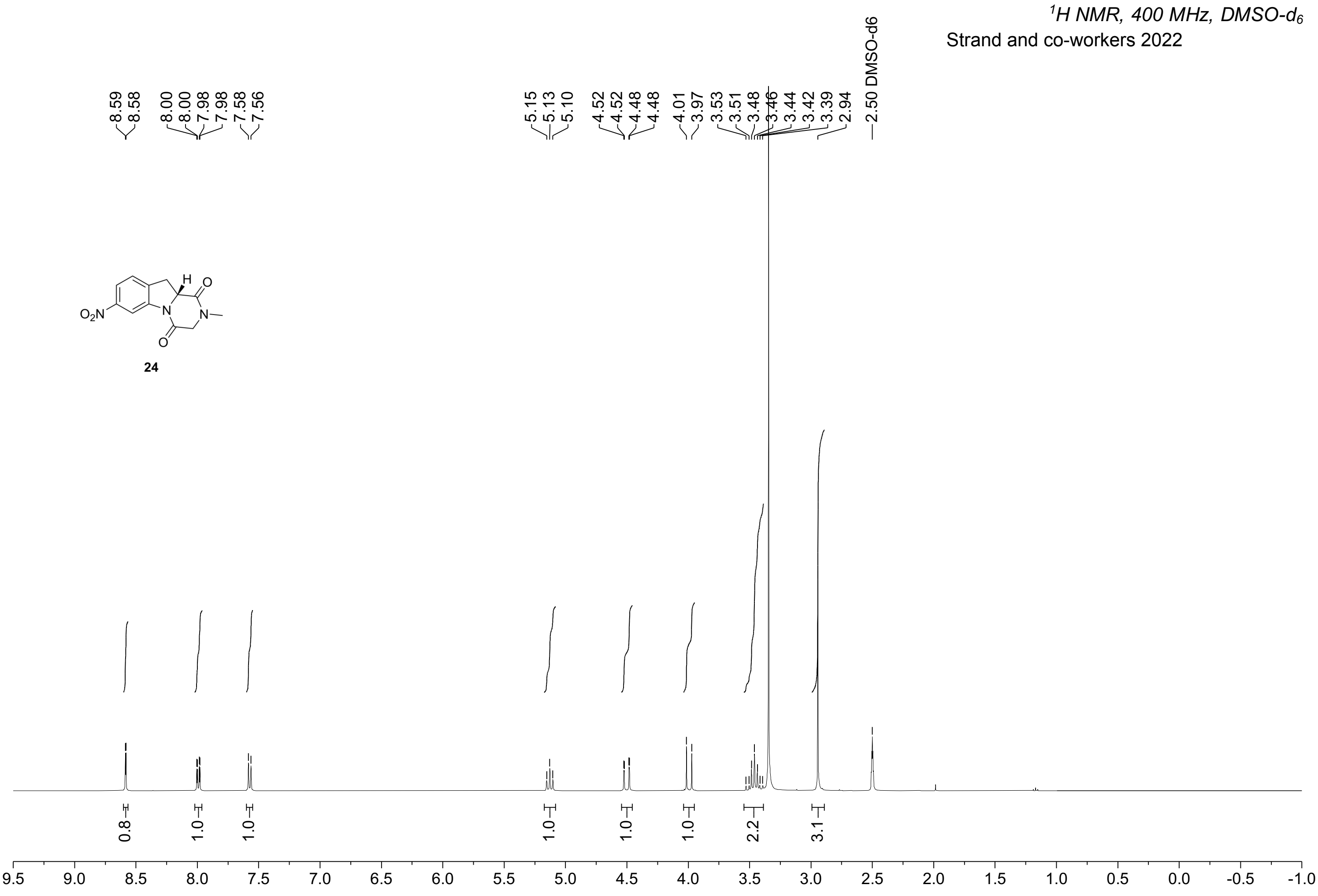
1H NMR, 400 MHz, DMSO-d6
Strand and co-workers 2022
8.59 8.58 8.00 8.00 7.98 7.98 7.58 7.56
5.15 5.13 5.10 4.52 4.52 4.48 4.48 4.01 3.97 3.53 3.51 3.48 3.46 3.44 3.42 3.39 2.94
2.50 DMSO-d6
0.8 1.0 1.0 1.0 1.0 1.0 2.2 3.1
O2N
H
O
N
N
O
24

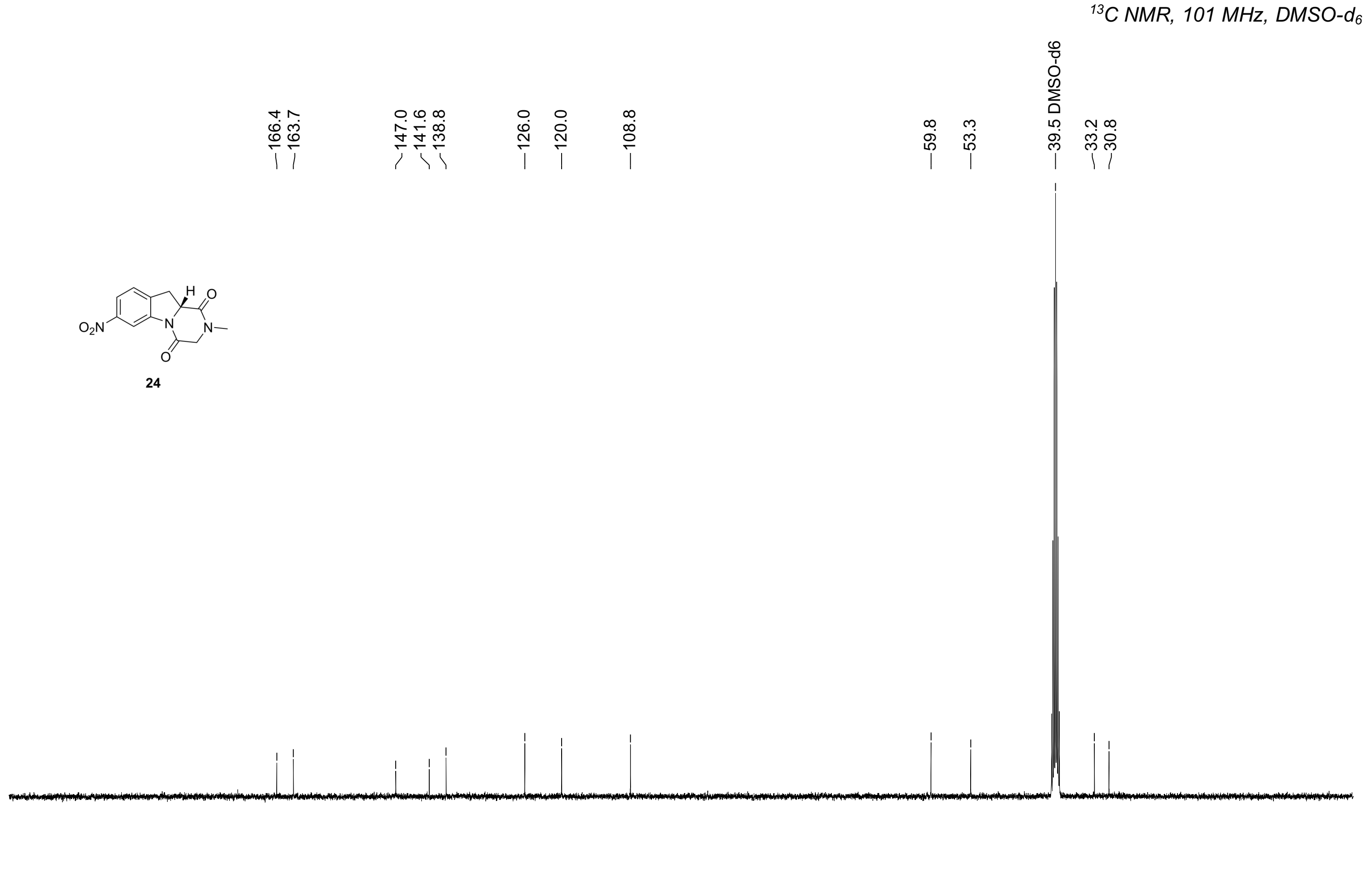
13C NMR, 101 MHz, DMSO-d6
166.4 163.7 147.0 141.6 138.8 126.0 120.0 108.8 59.8 53.3
39.5 DMSO-d6
33.2 30.8
O2N
H
O
N
N
O
24

^{1}H NMR, 400 MHz, DMSO-d_6
Strand and co-workers 2022

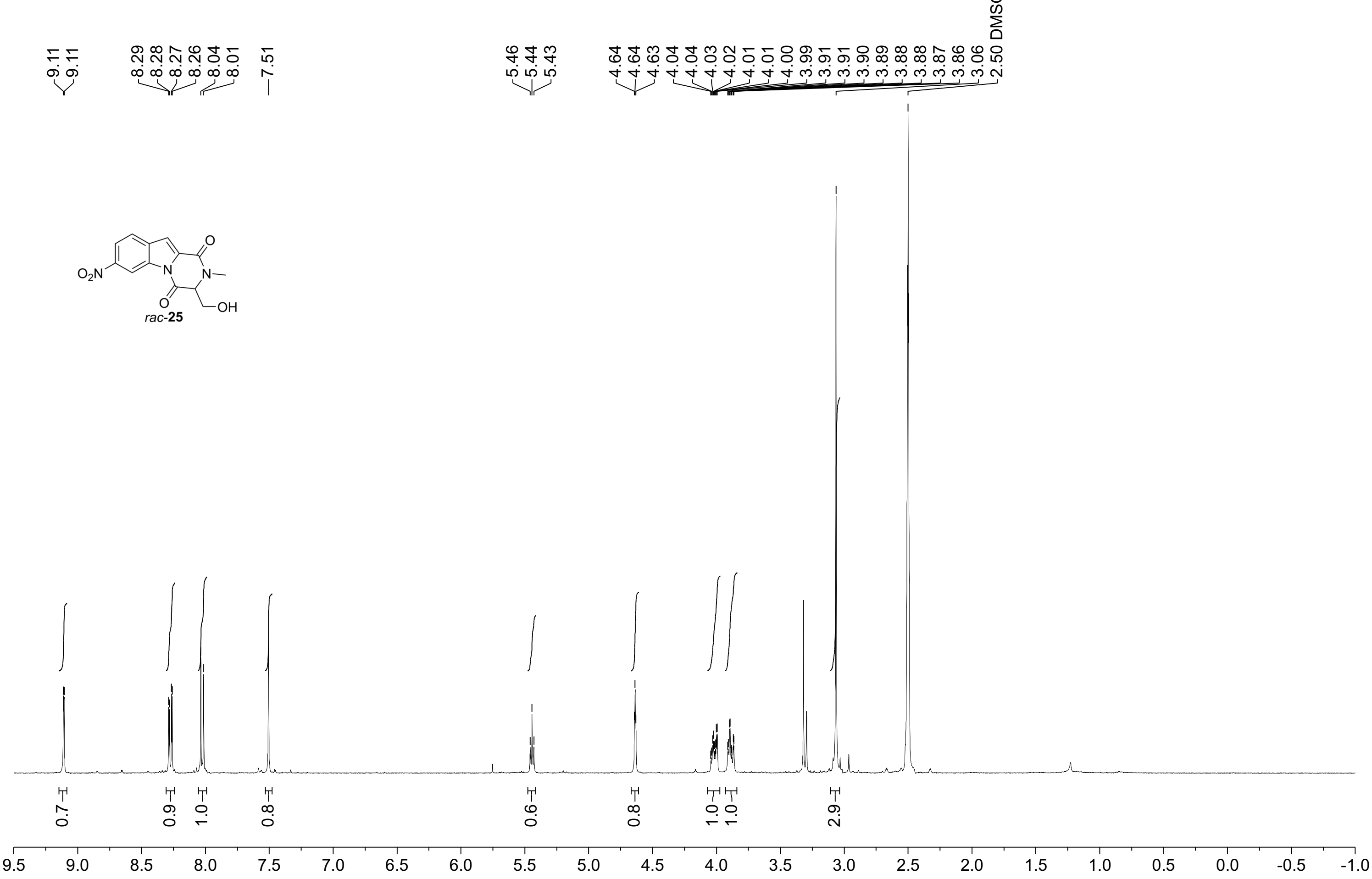

^{13}C NMR, 101 MHz, DMSO-d_6

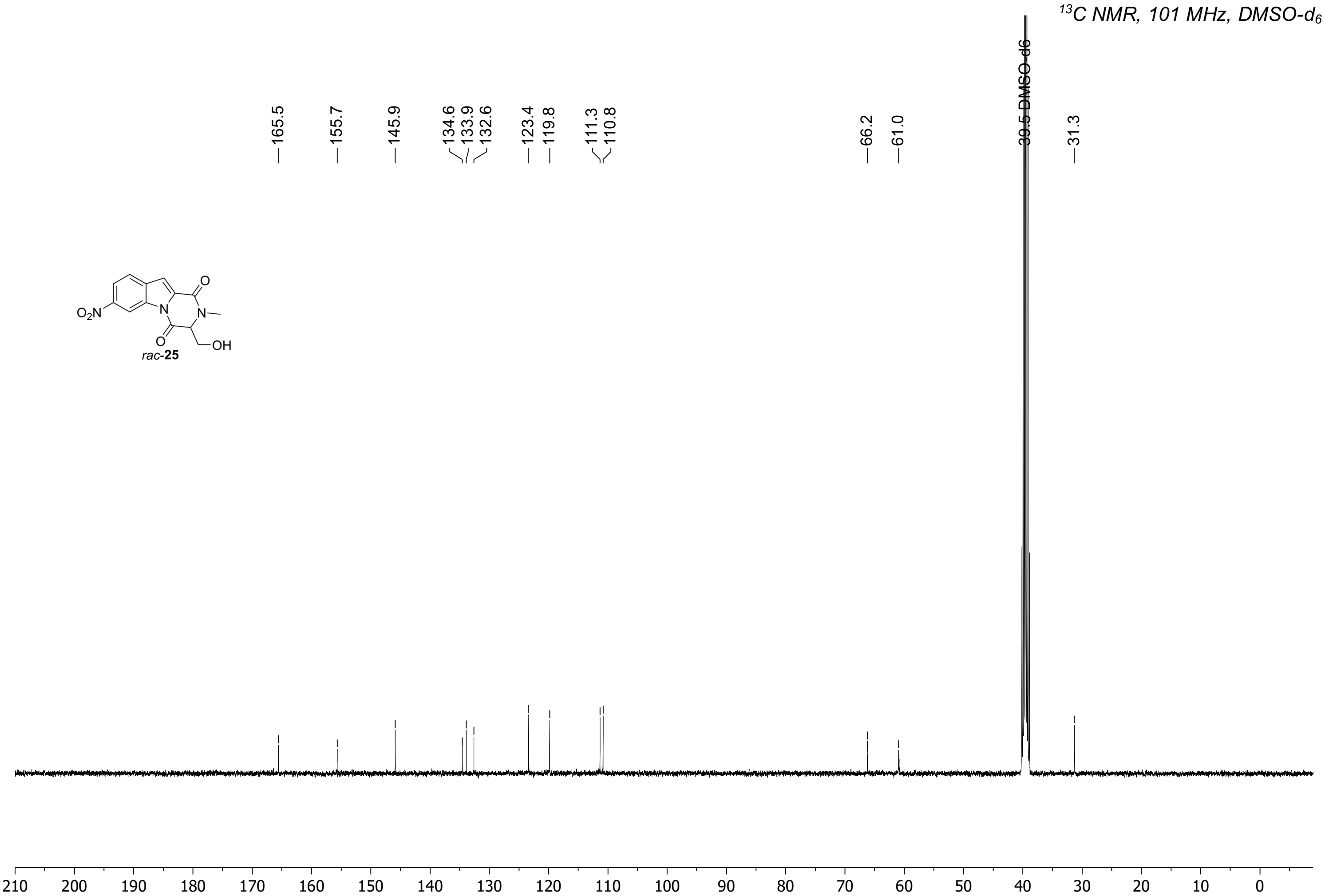

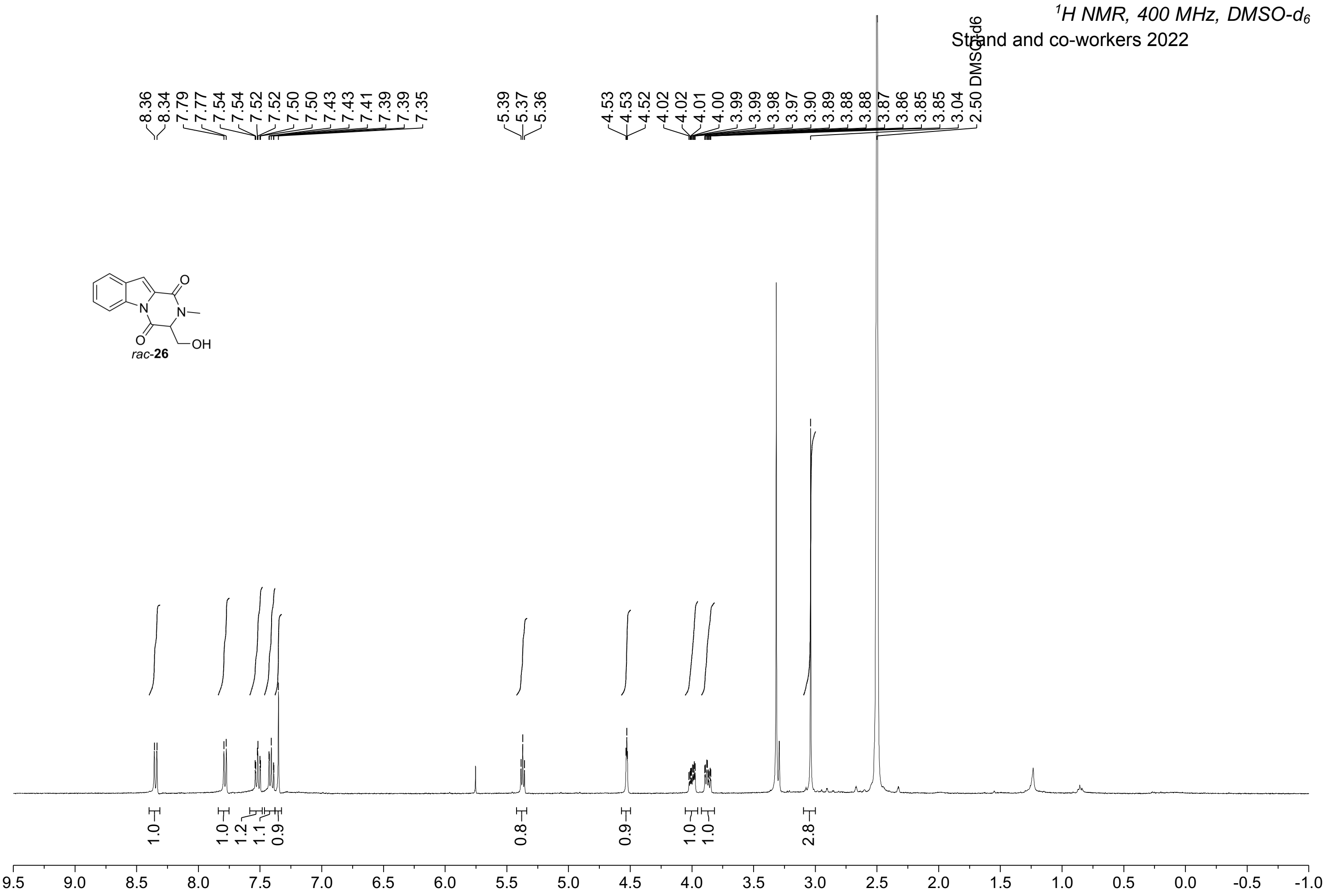
^{1}H NMR, 400 MHz, DMSO-d6
Strand and co-workers 2022
8.36 8.34 7.79 7.77 7.54 7.54 7.52 7.52 7.50 7.50 7.43 7.43 7.41 7.39 7.39 7.35
5.39 5.37 5.36
4.53 4.53 4.52 4.02 4.02 4.01 4.00 3.99 3.99 3.98 3.97 3.90 3.89 3.88 3.88 3.87 3.86 3.85 3.85 3.04
2.50 DMSO-d6
rac-26
1.0 1.0 1.2 1.1 0.9 0.8 0.9 1.0 1.0 2.8
9.5 9.0 8.5 8.0 7.5 7.0 6.5 6.0 5.5 5.0 4.5 4.0 3.5 3.0 2.5 2.0 1.5 1.0 0.5 0.0 -0.5 -1.0

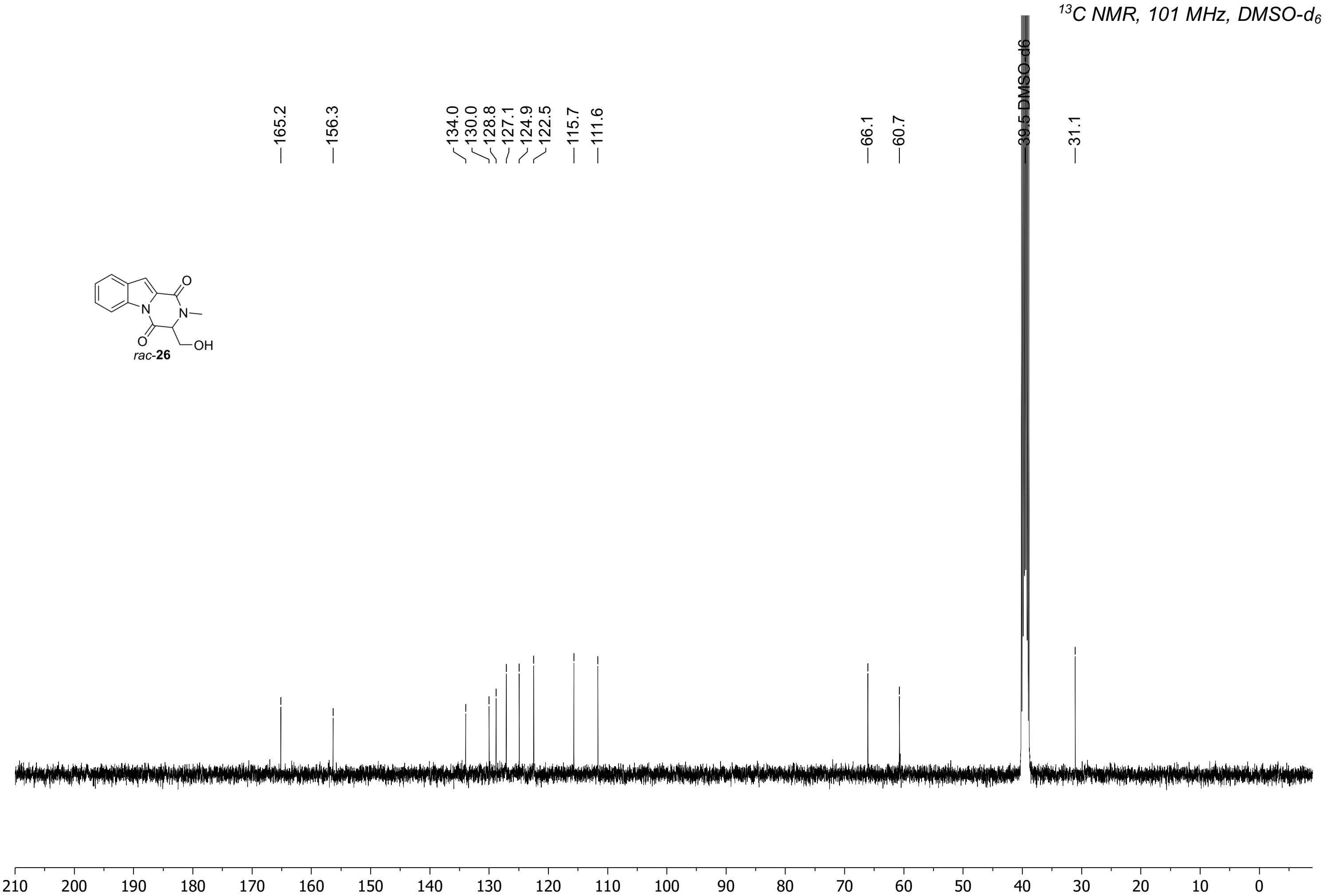
^{13}C NMR, 101 MHz, DMSO-d6
165.2 156.3 134.0 130.0 128.8 127.1 124.9 122.5 115.7 111.6 66.1 60.7 39.5 DMSO-d6 31.1
rac-26
210 200 190 180 170 160 150 140 130 120 110 100 90 80 70 60 50 40 30 20 10 0

^{1}H NMR, 400 MHz, $CDCl_3$
Strand and co-workers 2022

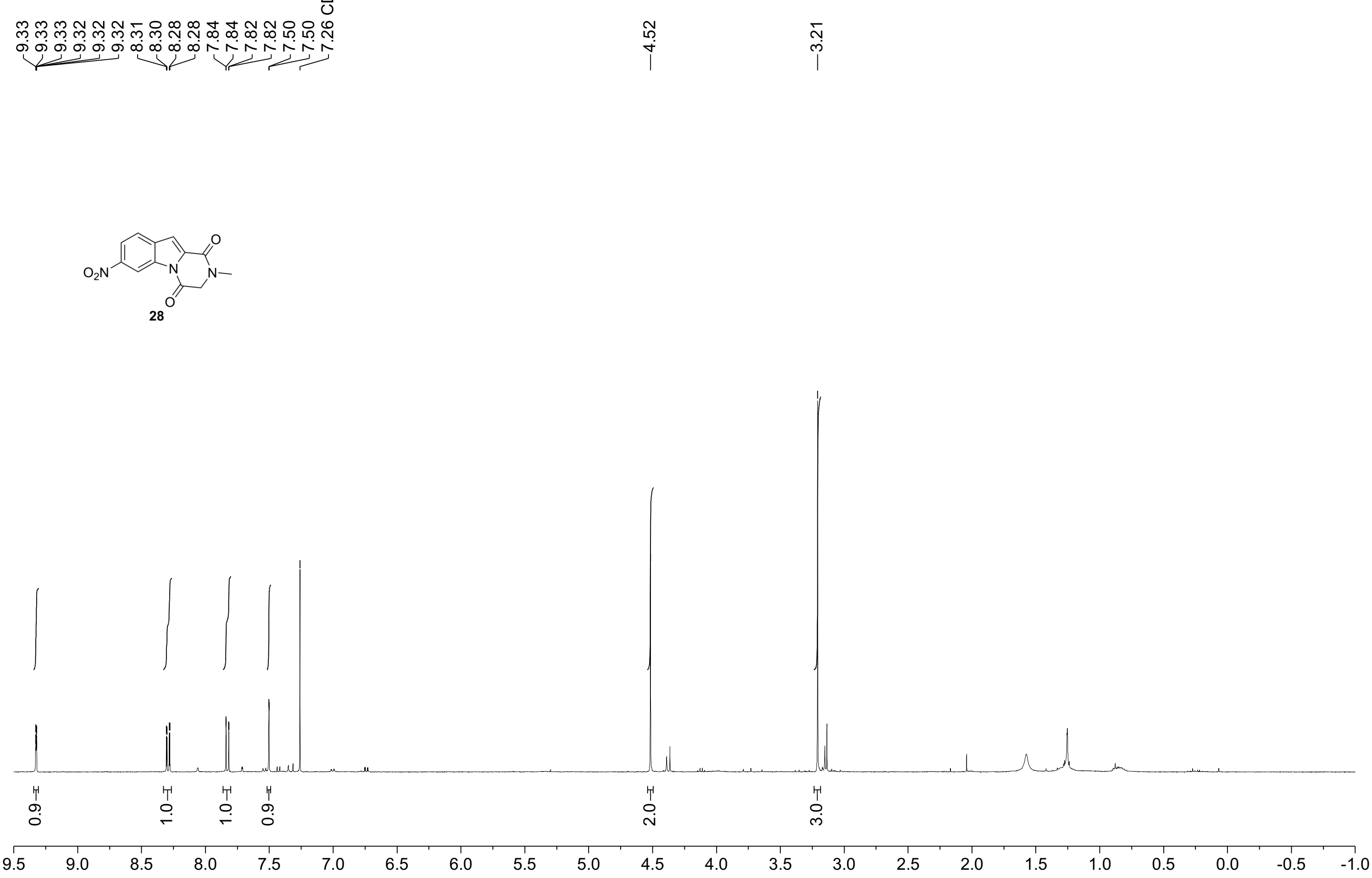

^{13}C NMR, 101 MHz, $CDCl_3$

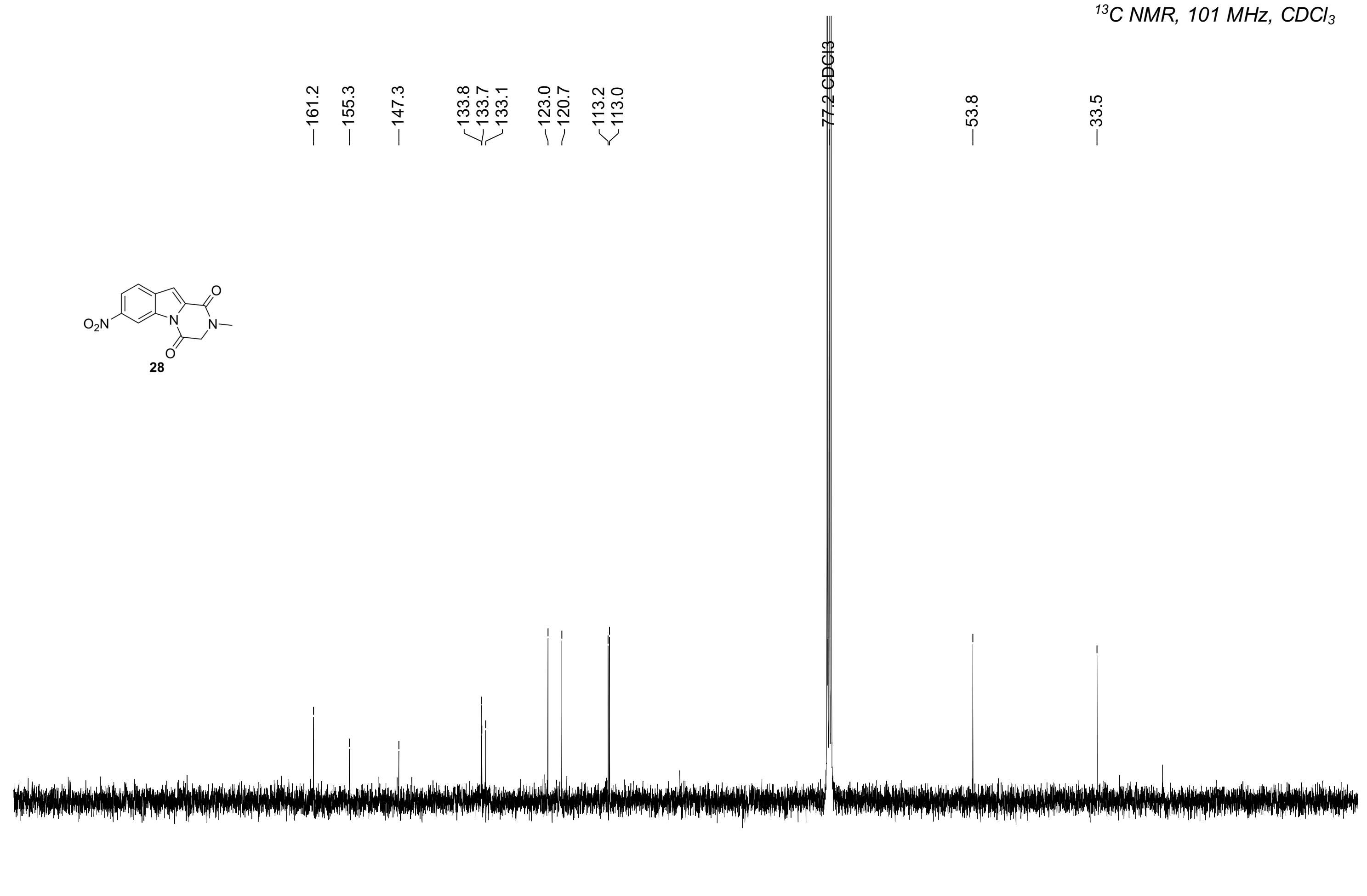

^{1}H NMR, 400 MHz, $CDCl_3$

Strand and co-workers 2022

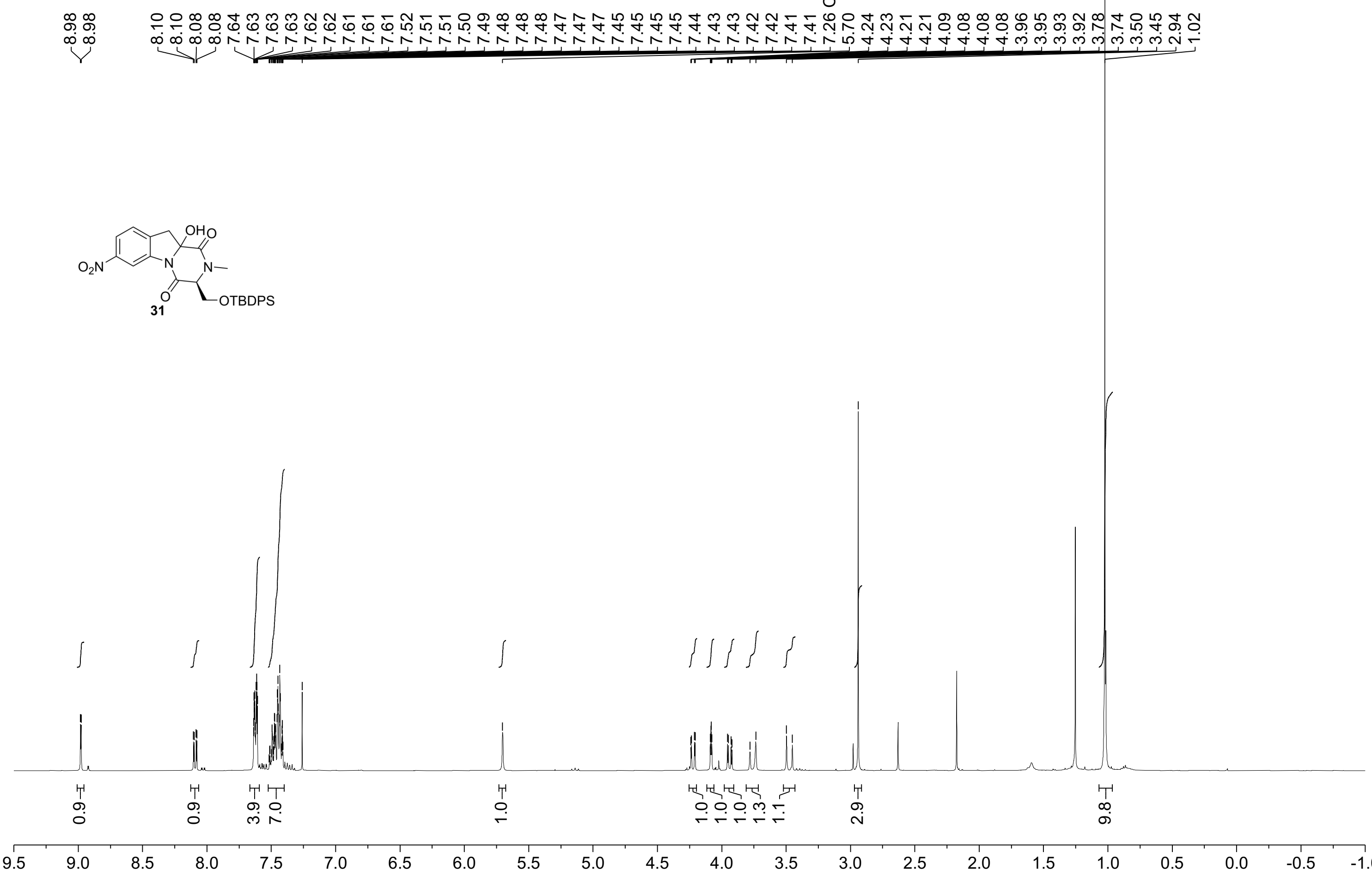

^{13}C NMR, 101 MHz, $CDCl_3$

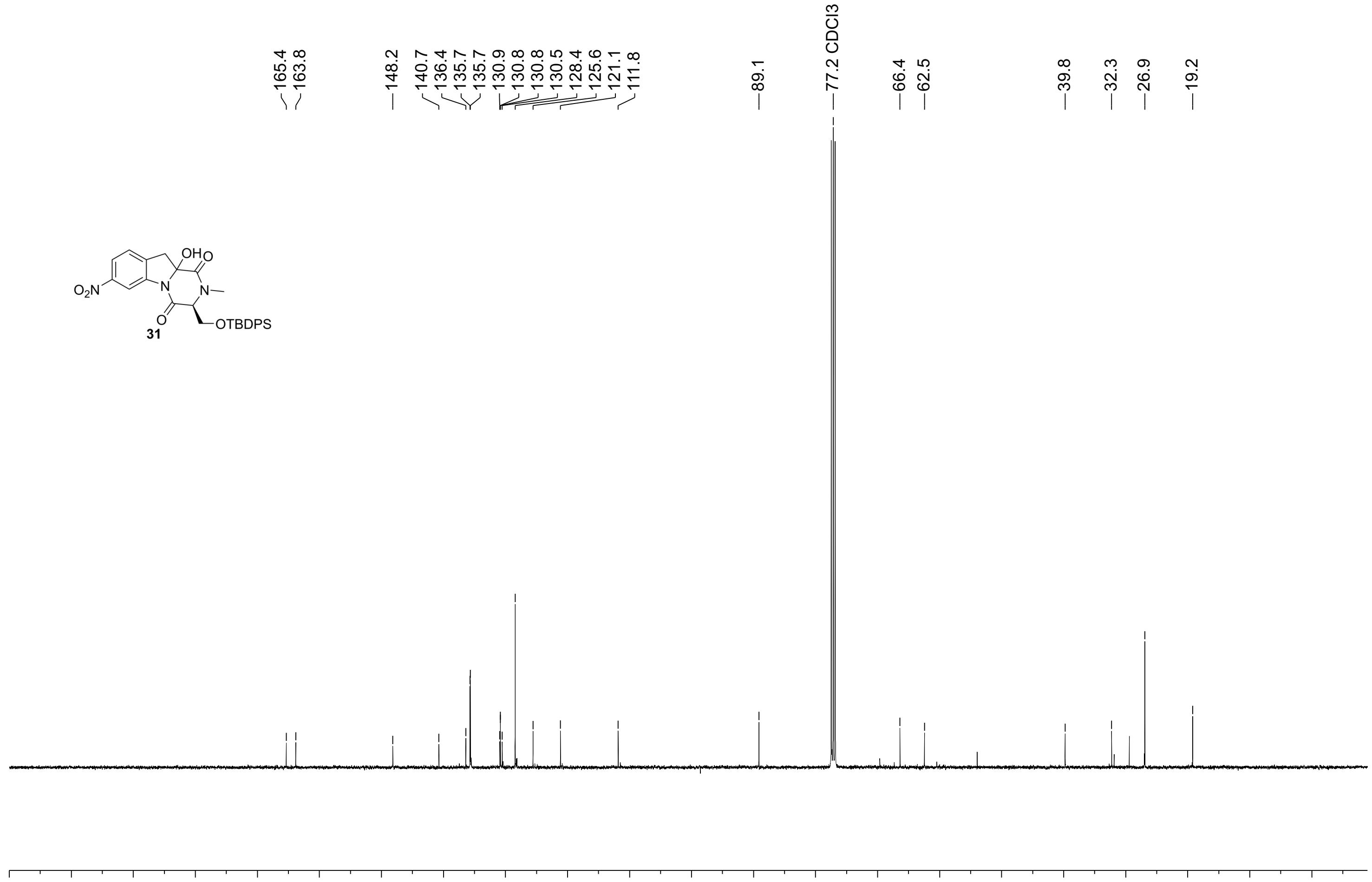

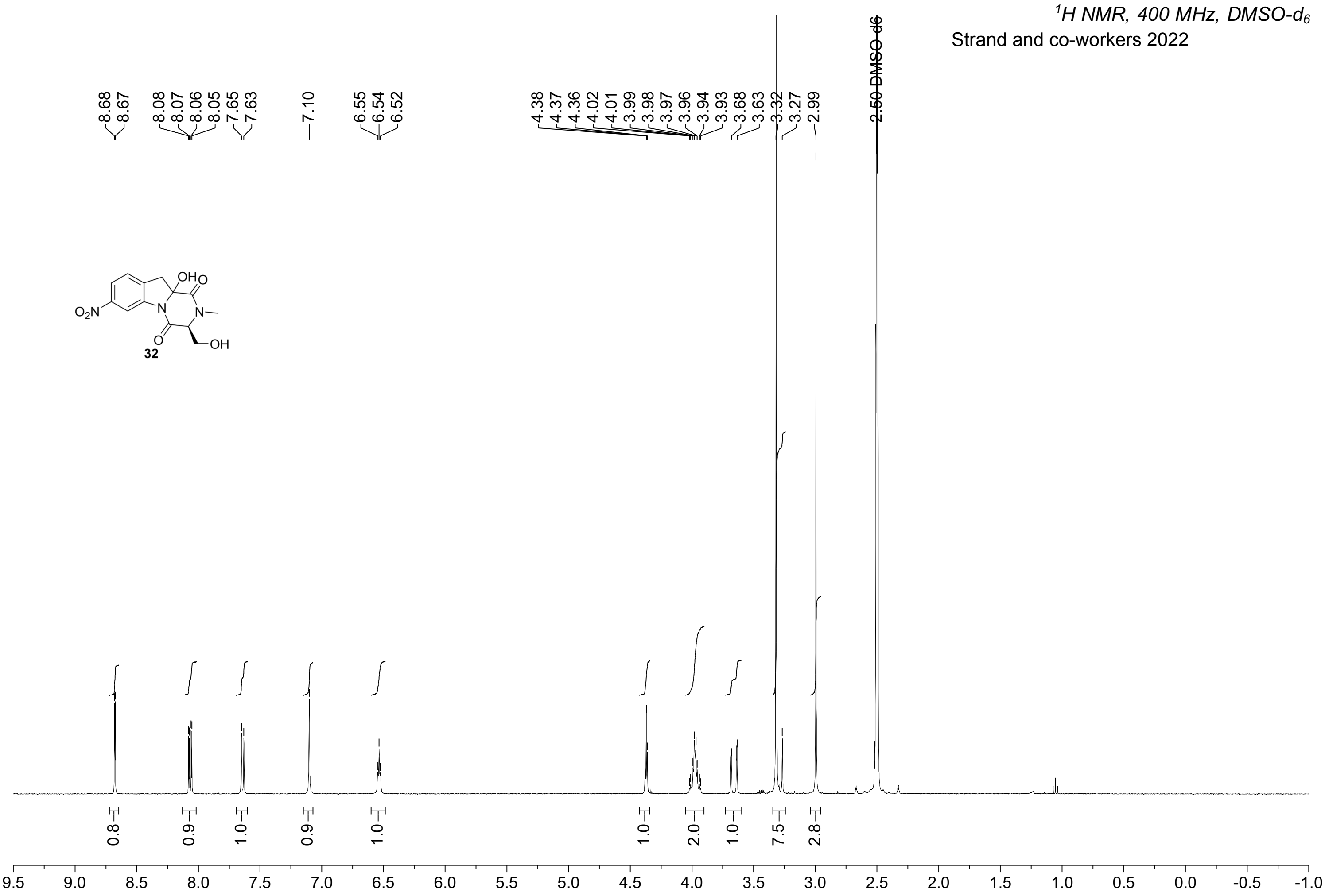
1H NMR, 400 MHz, DMSO-d6
Strand and co-workers 2022
8.68
8.67
8.08
8.07
8.06
8.05
7.65
7.63
7.10
6.55
6.54
6.52
4.38
4.37
4.36
4.02
4.01
3.99
3.98
3.97
3.96
3.94
3.93
3.68
3.63
3.32
3.27
2.99
2.50 DMSO-d6
O2N
OH
O
N
N
O
OH
32
0.8
0.9
1.0
0.9
1.0
1.0
2.0
1.0
7.5
2.8
9.5
9.0
8.5
8.0
7.5
7.0
6.5
6.0
5.5
5.0
4.5
4.0
3.5
3.0
2.5
2.0
1.5
1.0
0.5
0.0
-0.5
-1.0

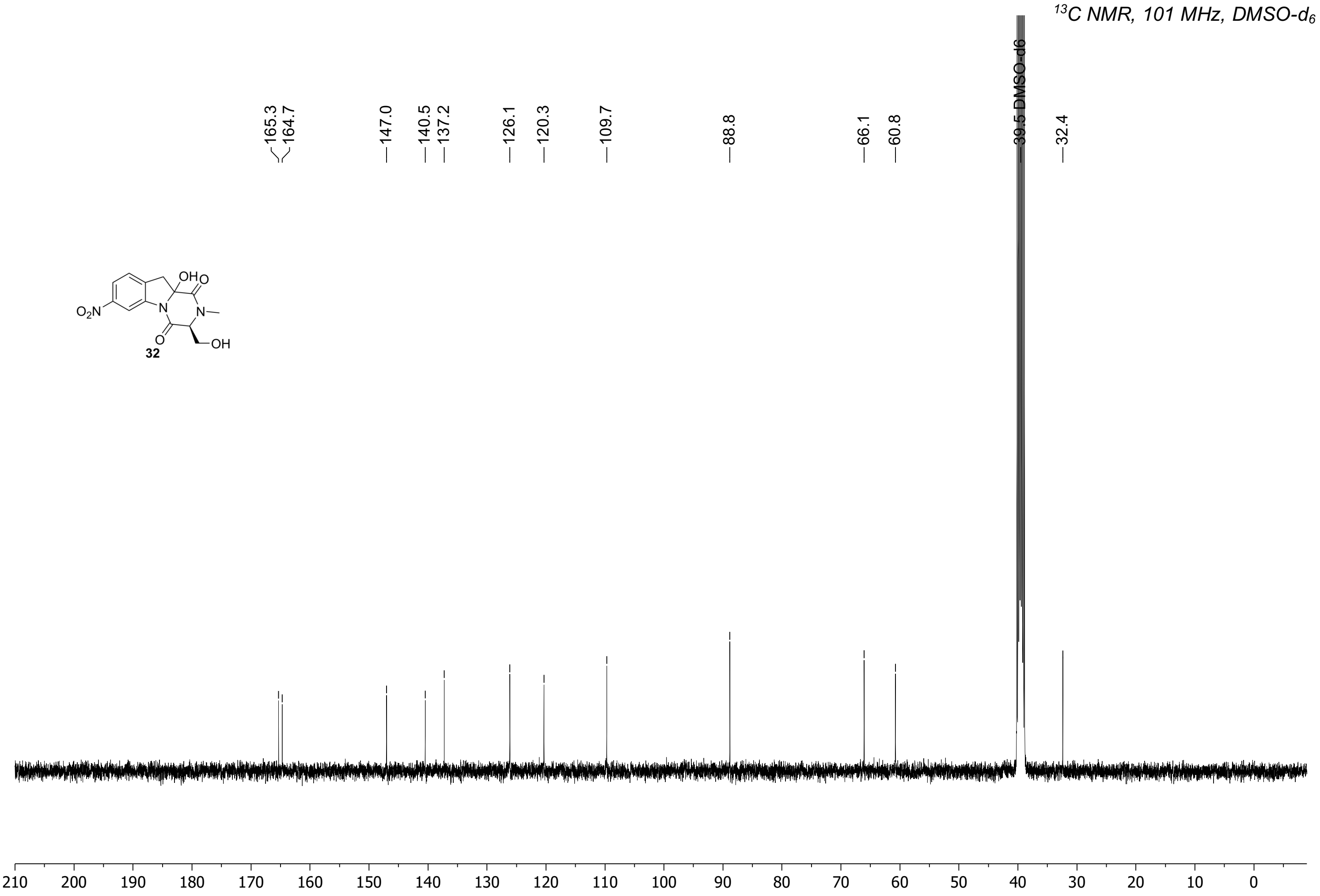
13C NMR, 101 MHz, DMSO-d6
165.3
164.7
147.0
140.5
137.2
126.1
120.3
109.7
88.8
66.1
60.8
39.5 DMSO-d6
32.4
O2N
OH
O
N
N
O
OH
32
210
200
190
180
170
160
150
140
130
120
110
100
90
80
70
60
50
40
30
20
10
0

^{1}H NMR, 400 MHz, $CDCl_3$

Strand and co-workers 2022

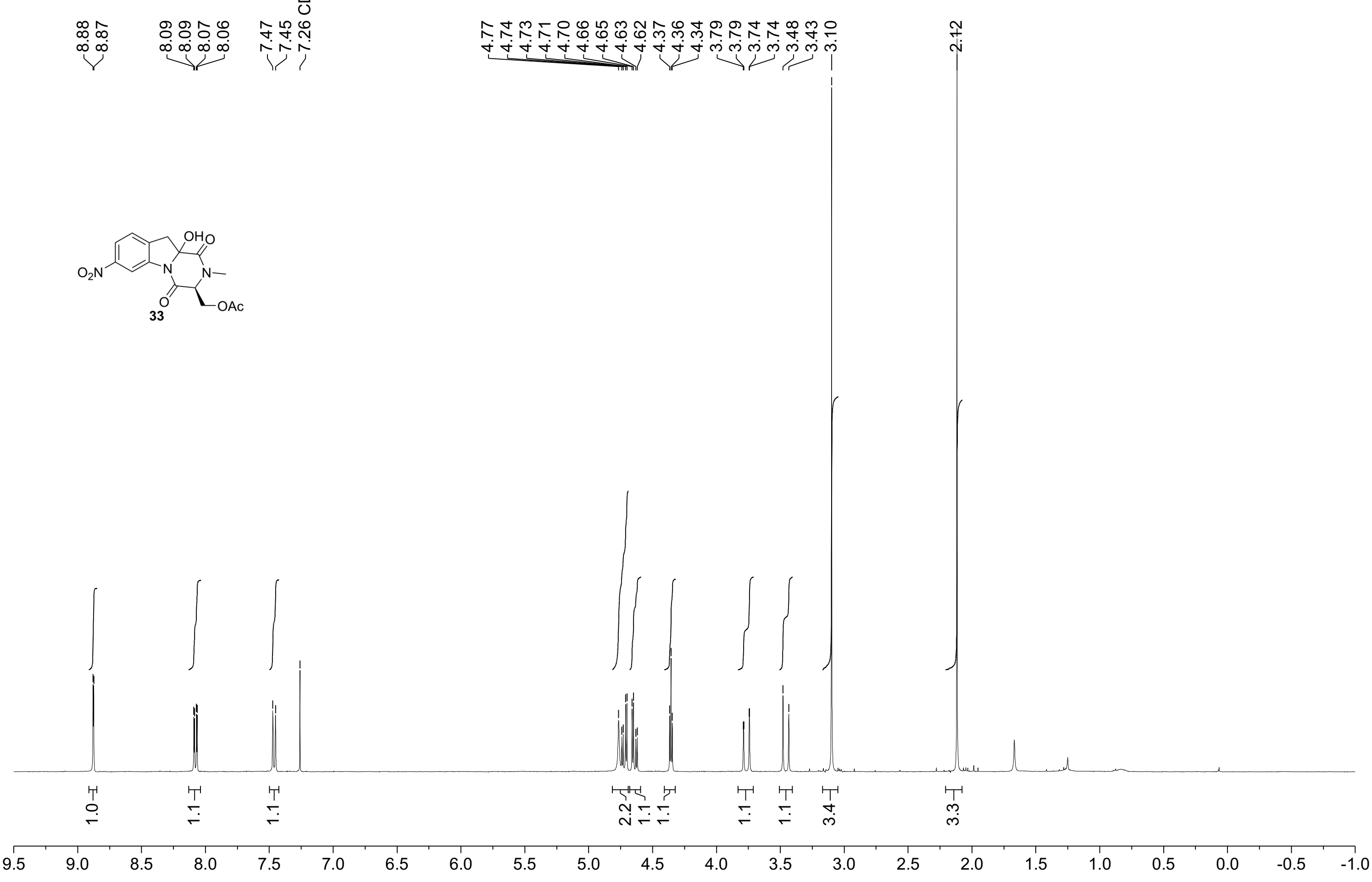

^{13}C NMR, 101 MHz, $CDCl_3$

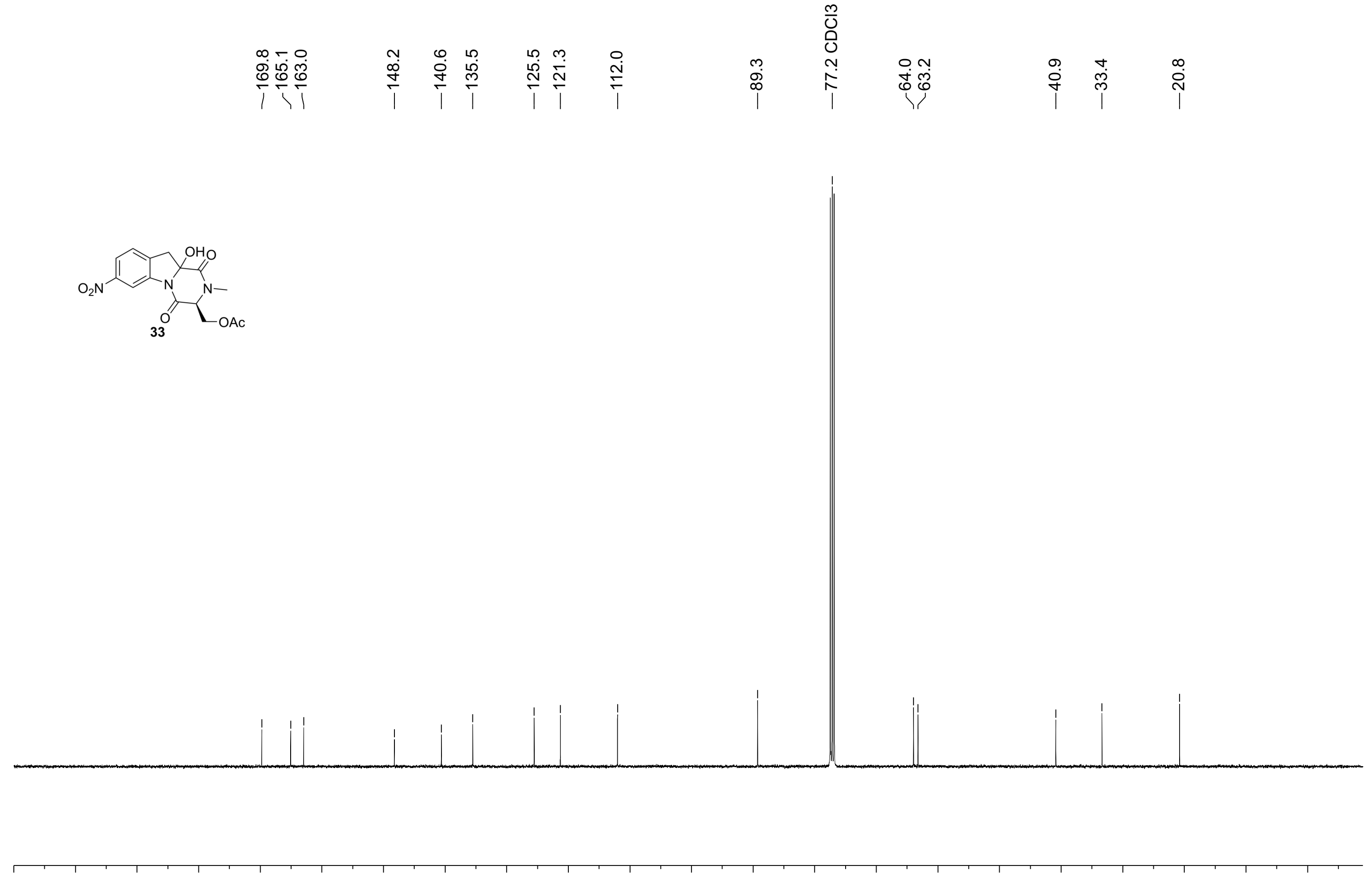

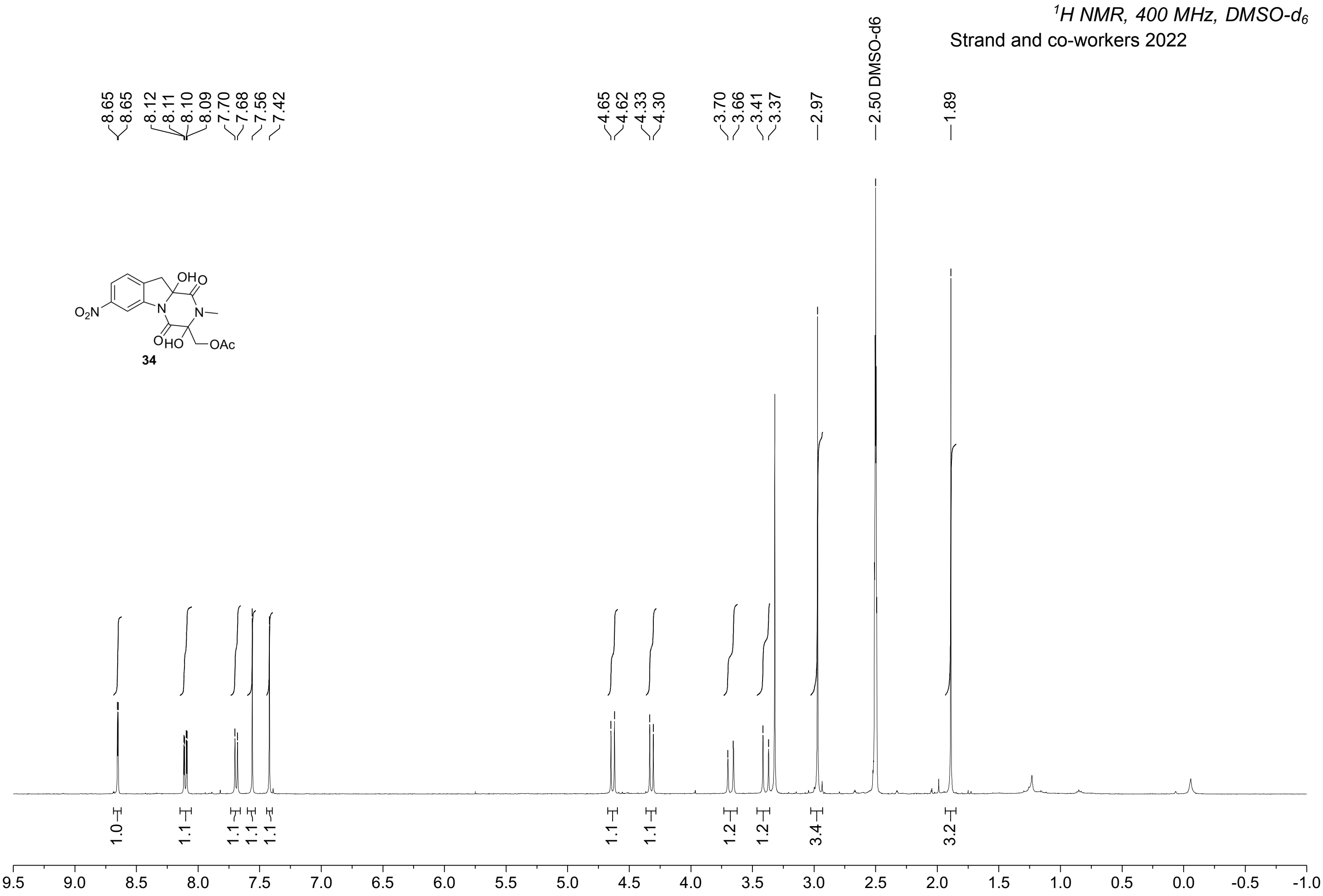

^{1}H NMR, 400 MHz, DMSO-d_6
Strand and co-workers 2022
8.65 8.65 8.12 8.11 8.10 8.09 7.70 7.68 7.56 7.42
4.65 4.62 4.33 4.30
3.70 3.66 3.41 3.37
2.97
2.50 DMSO-d6
1.89
O_2N OH O N N O HO OAc
34
1.0 1.1 1.1 1.1 1.1 1.1 1.1 1.2 1.2 3.4 3.2
9.5 9.0 8.5 8.0 7.5 7.0 6.5 6.0 5.5 5.0 4.5 4.0 3.5 3.0 2.5 2.0 1.5 1.0 0.5 0.0 -0.5 -1.0

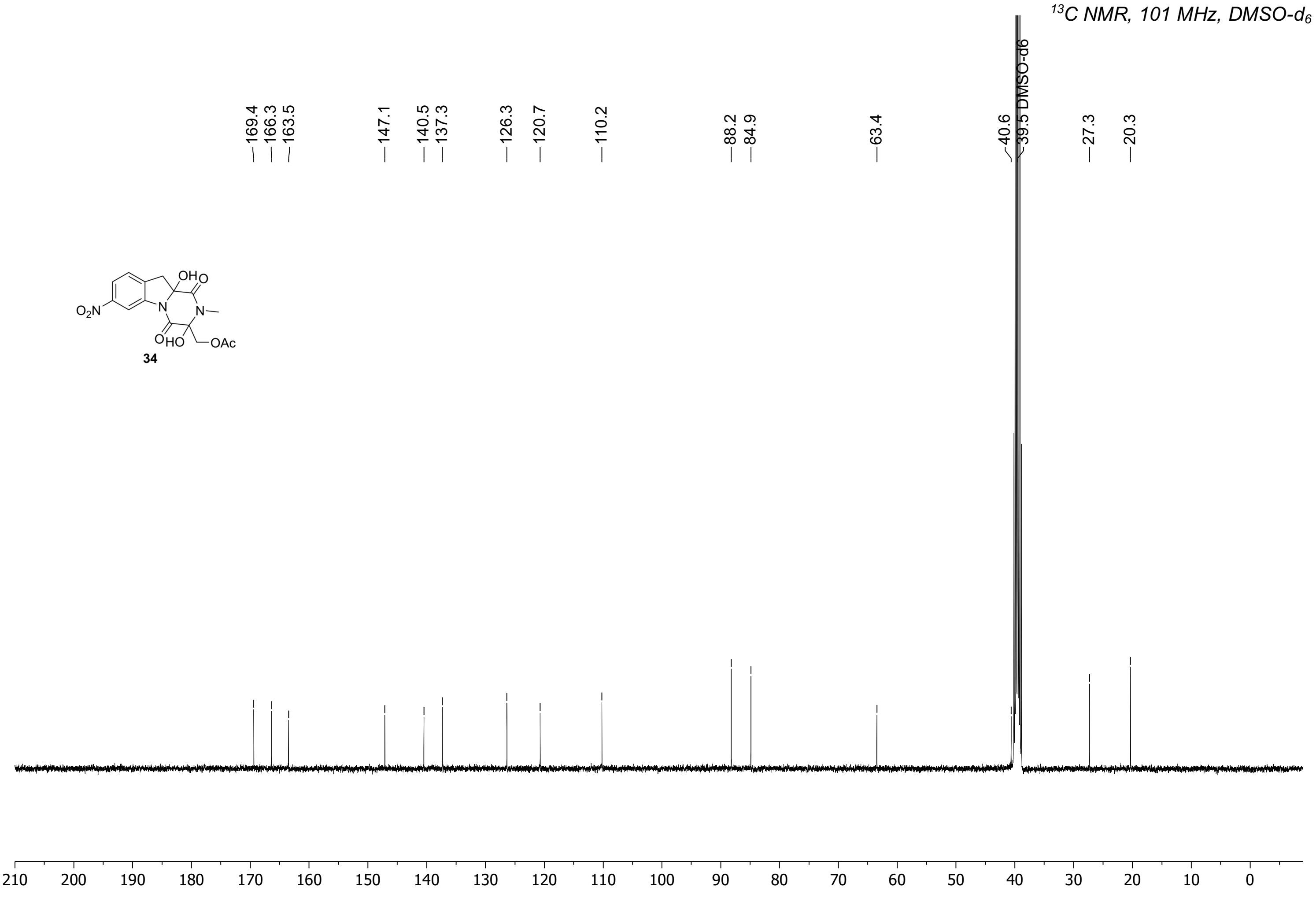

^{13}C NMR, 101 MHz, DMSO-d_6
169.4 166.3 163.5
147.1 140.5 137.3
126.3 120.7
110.2
88.2 84.9
63.4
40.6
39.5 DMSO-d6
27.3 20.3
O_2N OH O N N O HO OAc
34
210 200 190 180 170 160 150 140 130 120 110 100 90 80 70 60 50 40 30 20 10 0

^{1}H NMR, 400 MHz, $CDCl_3$
Strand and co-workers 2022

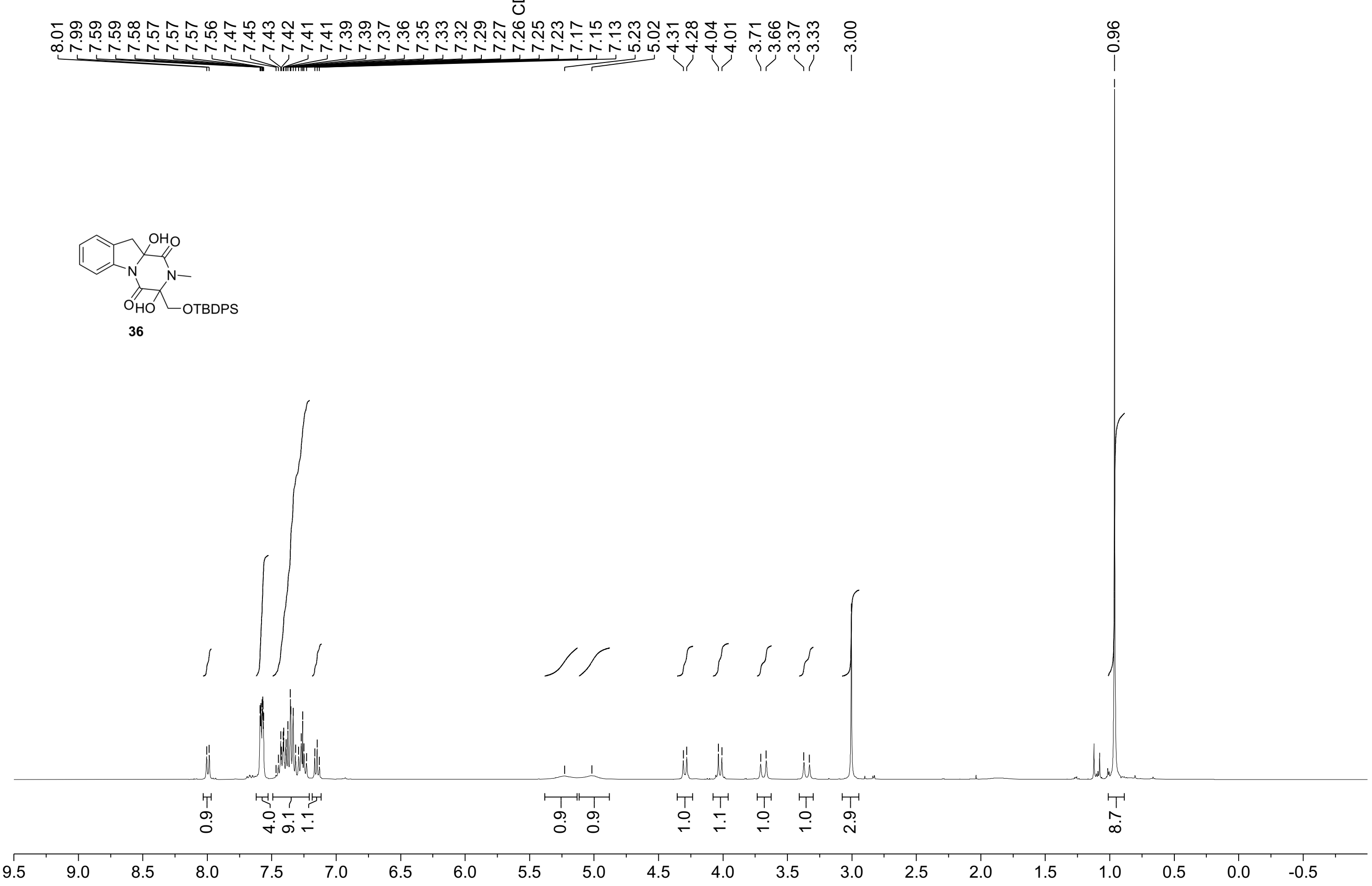

^{13}C NMR, 101 MHz, $CDCl_3$

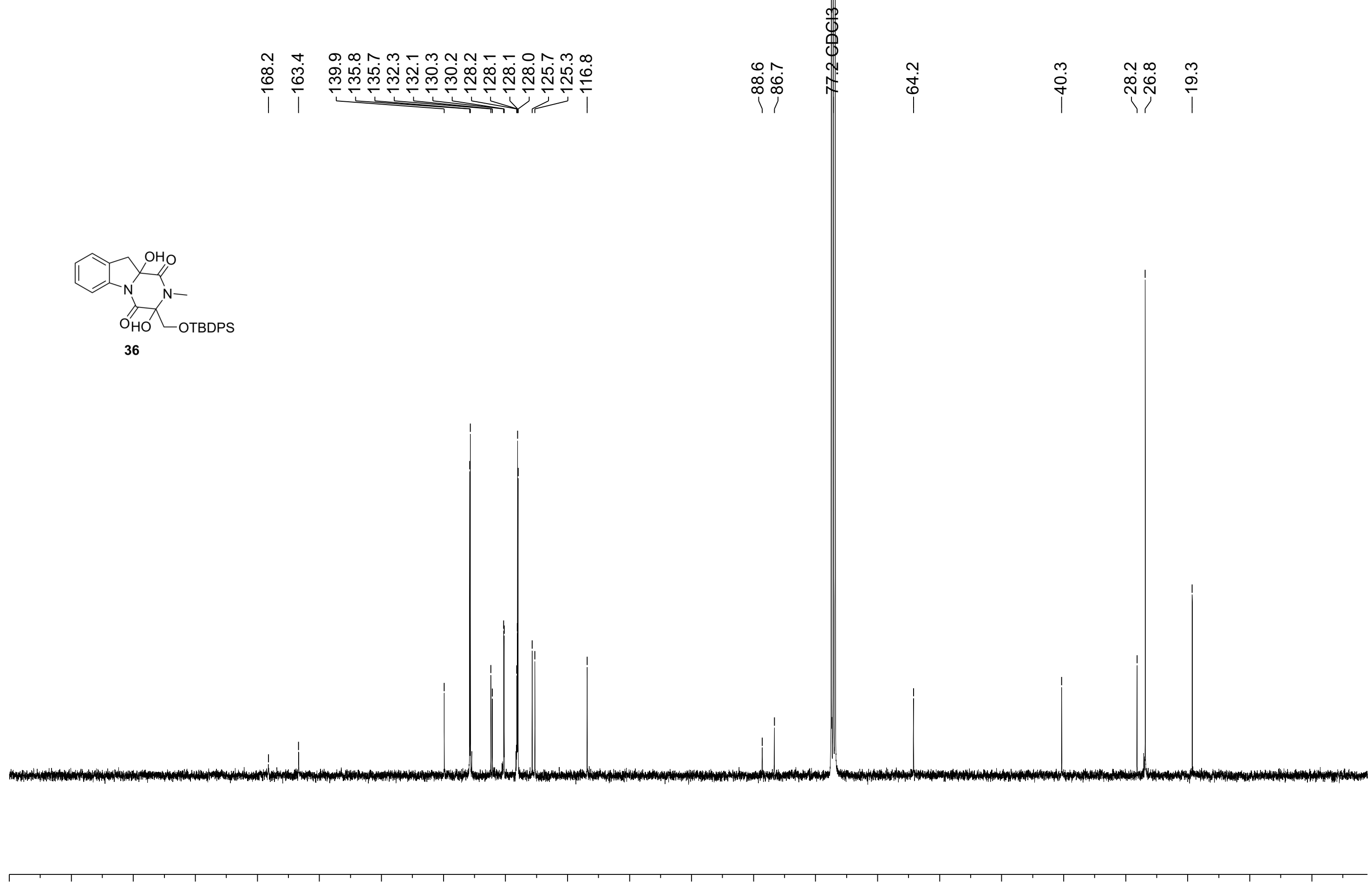

^{1}H NMR, 400 MHz, DMSO-d_6
Strand and co-workers 2022

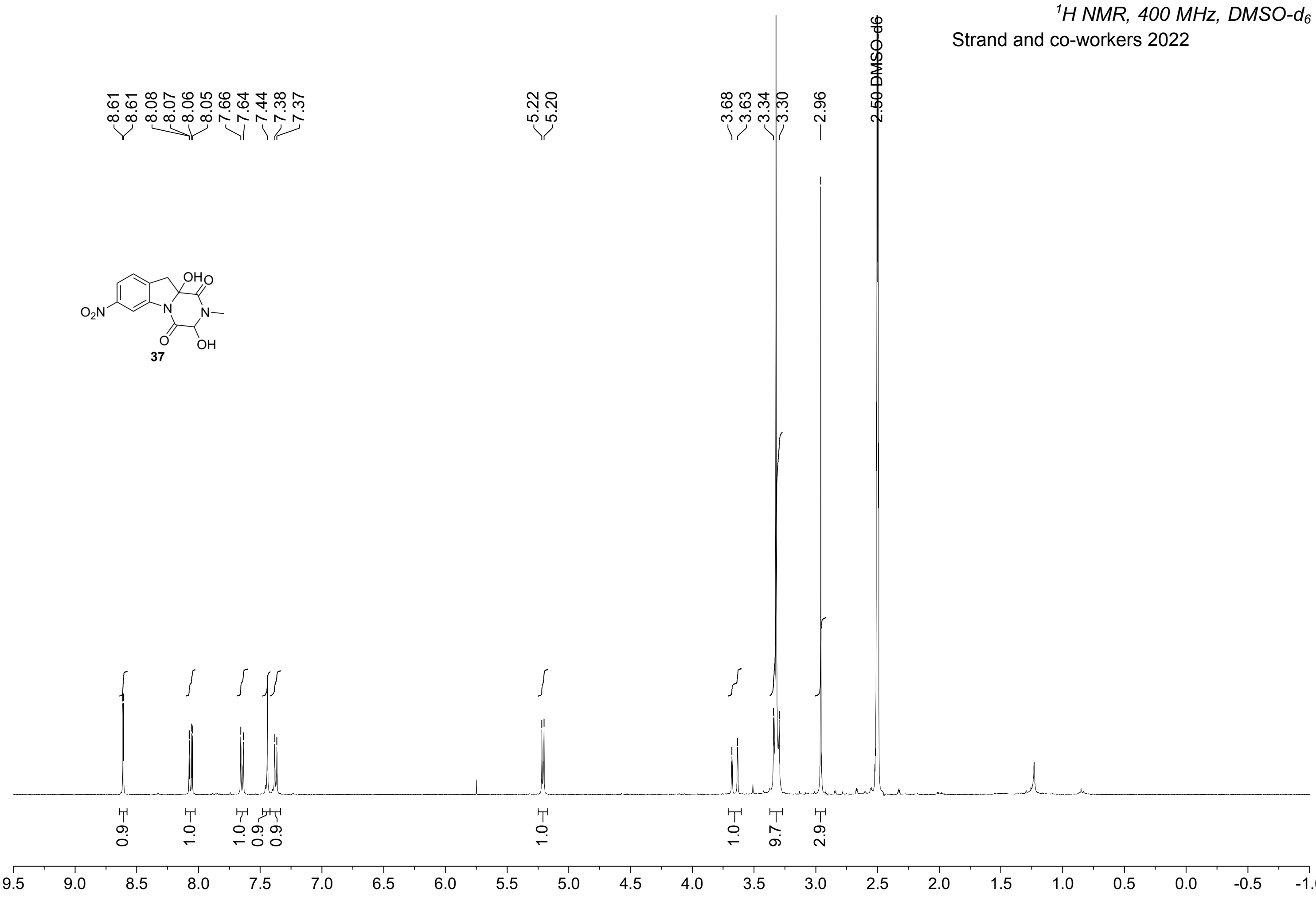

^{13}C NMR, 101 MHz, DMSO-d_6

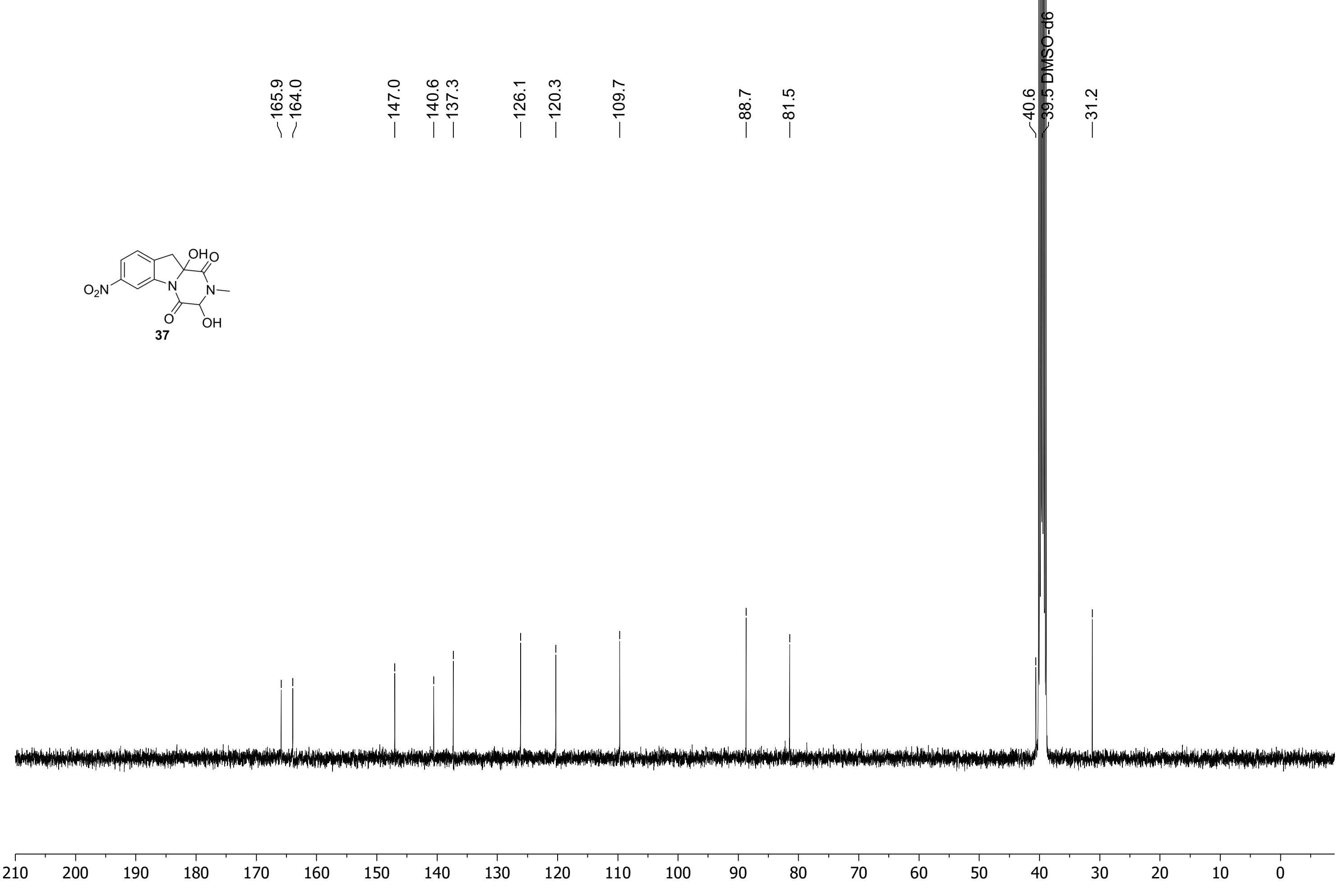

^{1}H NMR, 400 MHz, $CDCl_3$
Strand and co-workers 2022

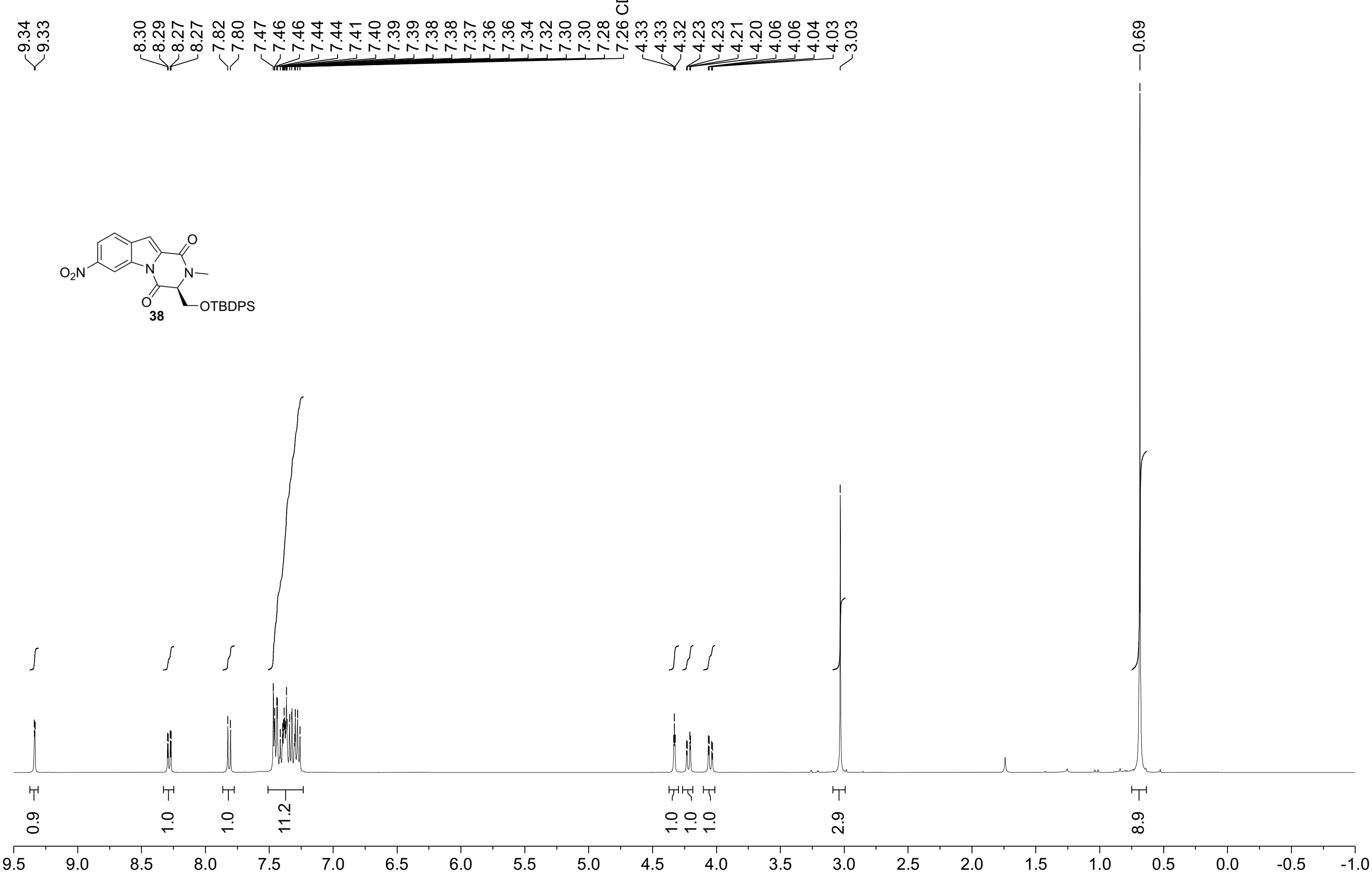

^{13}C NMR, 101 MHz, $CDCl_3$

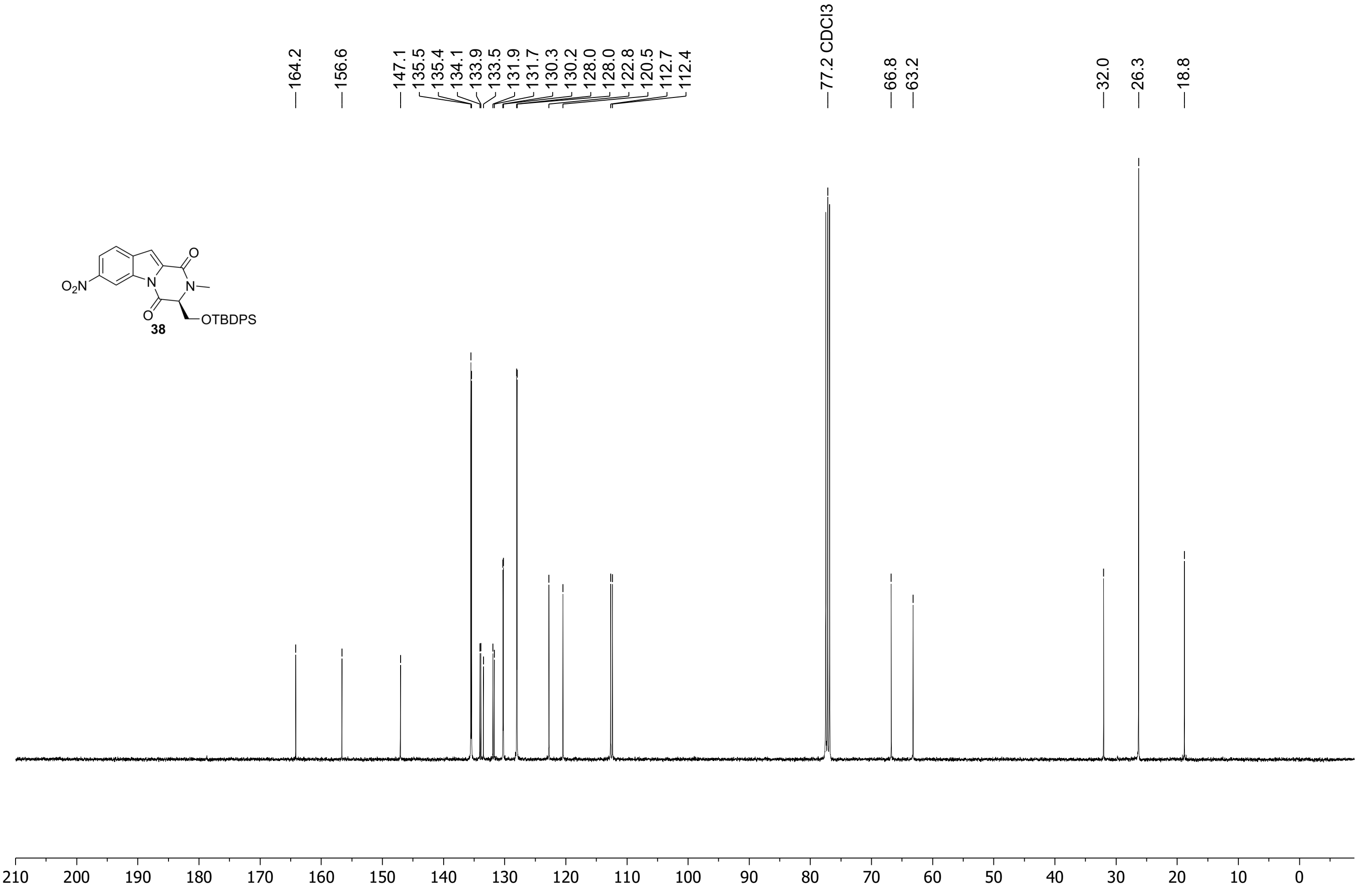

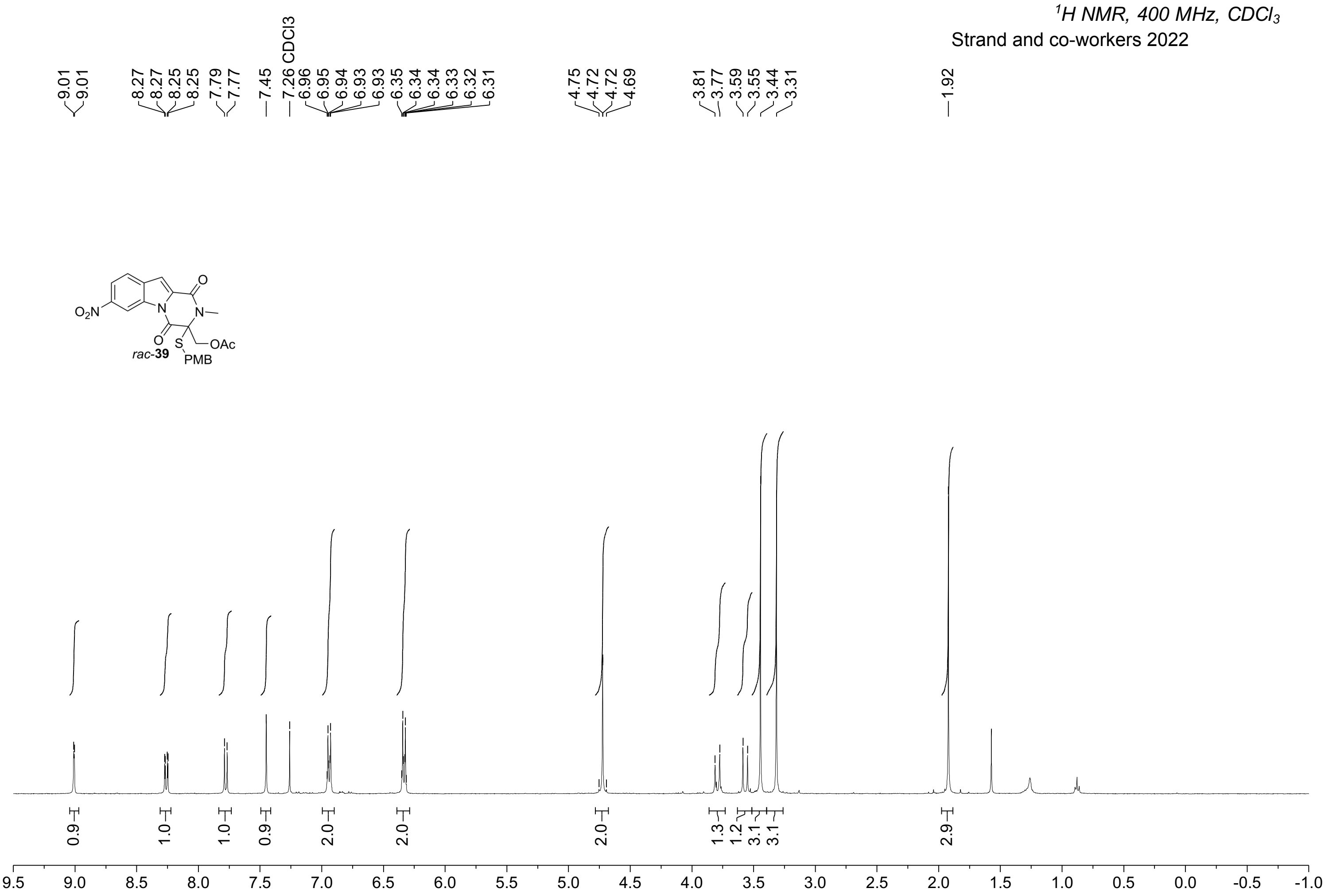

1H NMR, 400 MHz, CDCl3
Strand and co-workers 2022
9.01
9.01
8.27
8.27
8.25
8.25
7.79
7.77
7.45
7.26 CDCl3
6.96
6.95
6.94
6.93
6.93
6.35
6.34
6.34
6.33
6.32
6.31
4.75
4.72
4.72
4.69
3.81
3.77
3.59
3.55
3.44
3.31
1.92
O2N
N
N
O
O
S
PMB
OAc
rac-39
0.9
1.0
1.0
0.9
2.0
2.0
2.0
1.3
1.2
3.1
3.1
2.9
9.5 9.0 8.5 8.0 7.5 7.0 6.5 6.0 5.5 5.0 4.5 4.0 3.5 3.0 2.5 2.0 1.5 1.0 0.5 0.0 -0.5 -1.0

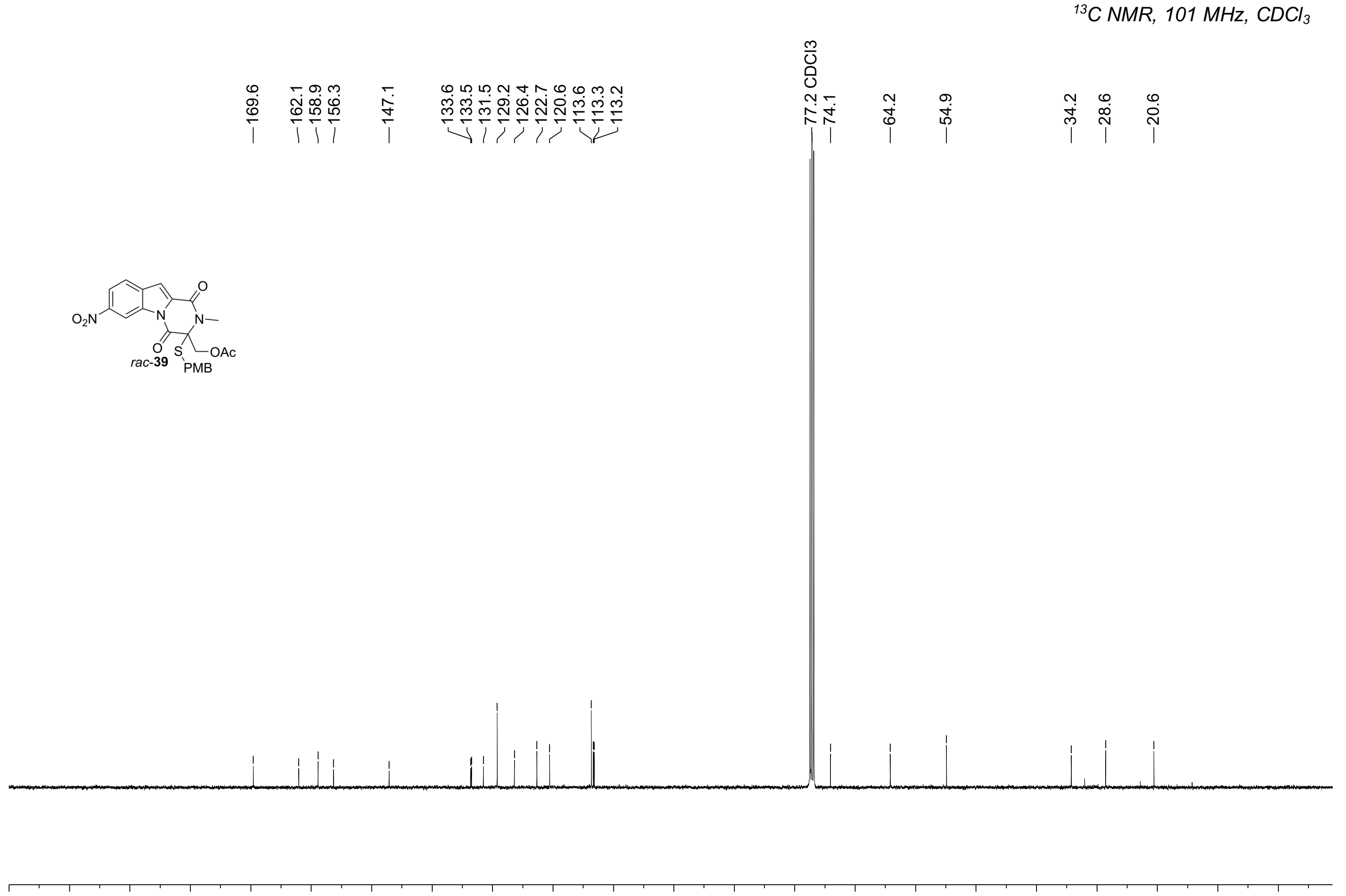

13C NMR, 101 MHz, CDCl3
169.6
162.1
158.9
156.3
147.1
133.6
133.5
131.5
129.2
126.4
122.7
120.6
113.6
113.3
113.2
77.2 CDCl3
74.1
64.2
54.9
34.2
28.6
20.6
O2N
N
N
O
O
S
PMB
OAc
rac-39
210 200 190 180 170 160 150 140 130 120 110 100 90 80 70 60 50 40 30 20 10 0

¹H NMR, 400 MHz, CDCl₃
Strand and co-workers 2022

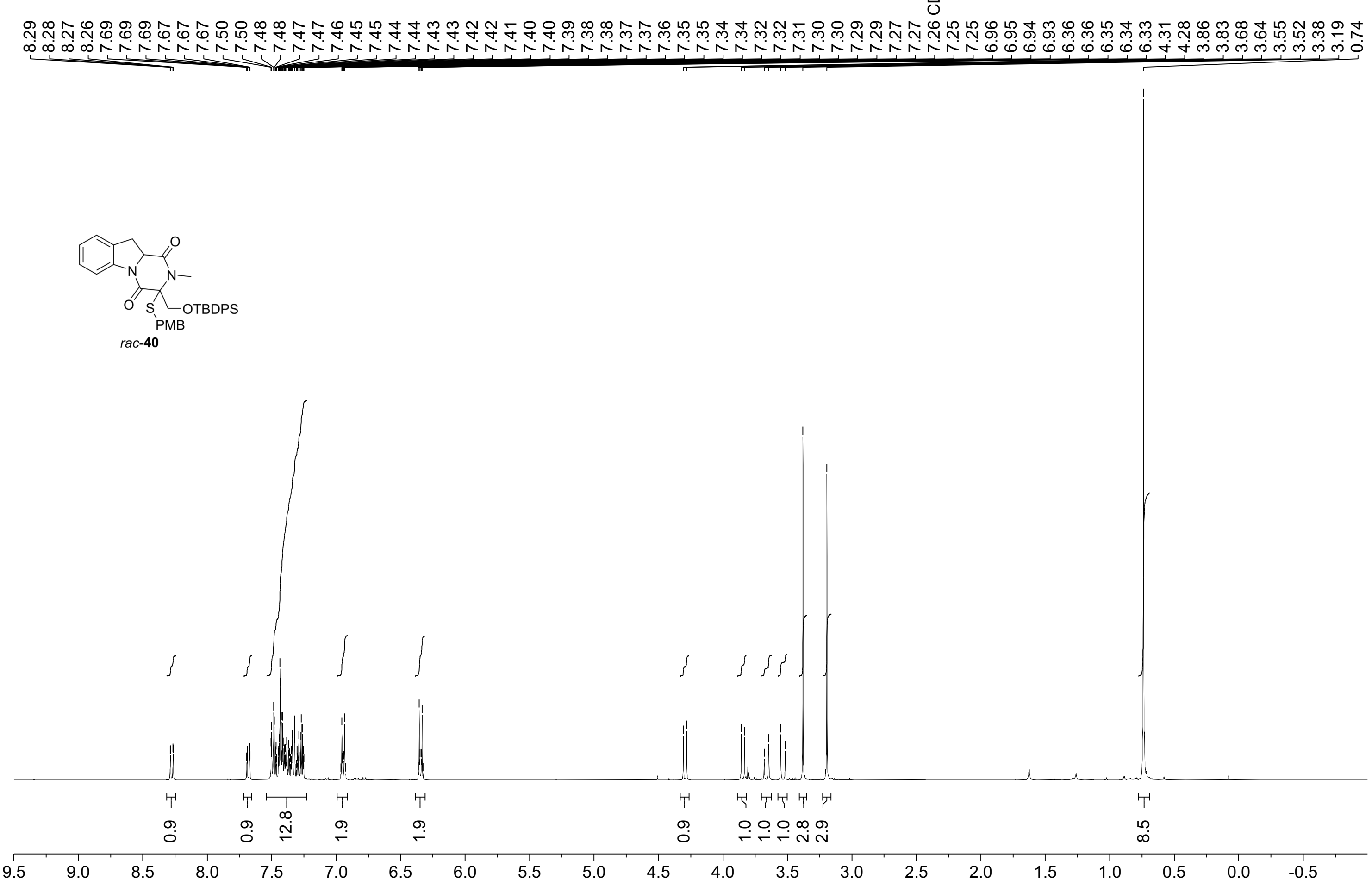

¹³C NMR, 101 MHz, CDCl₃

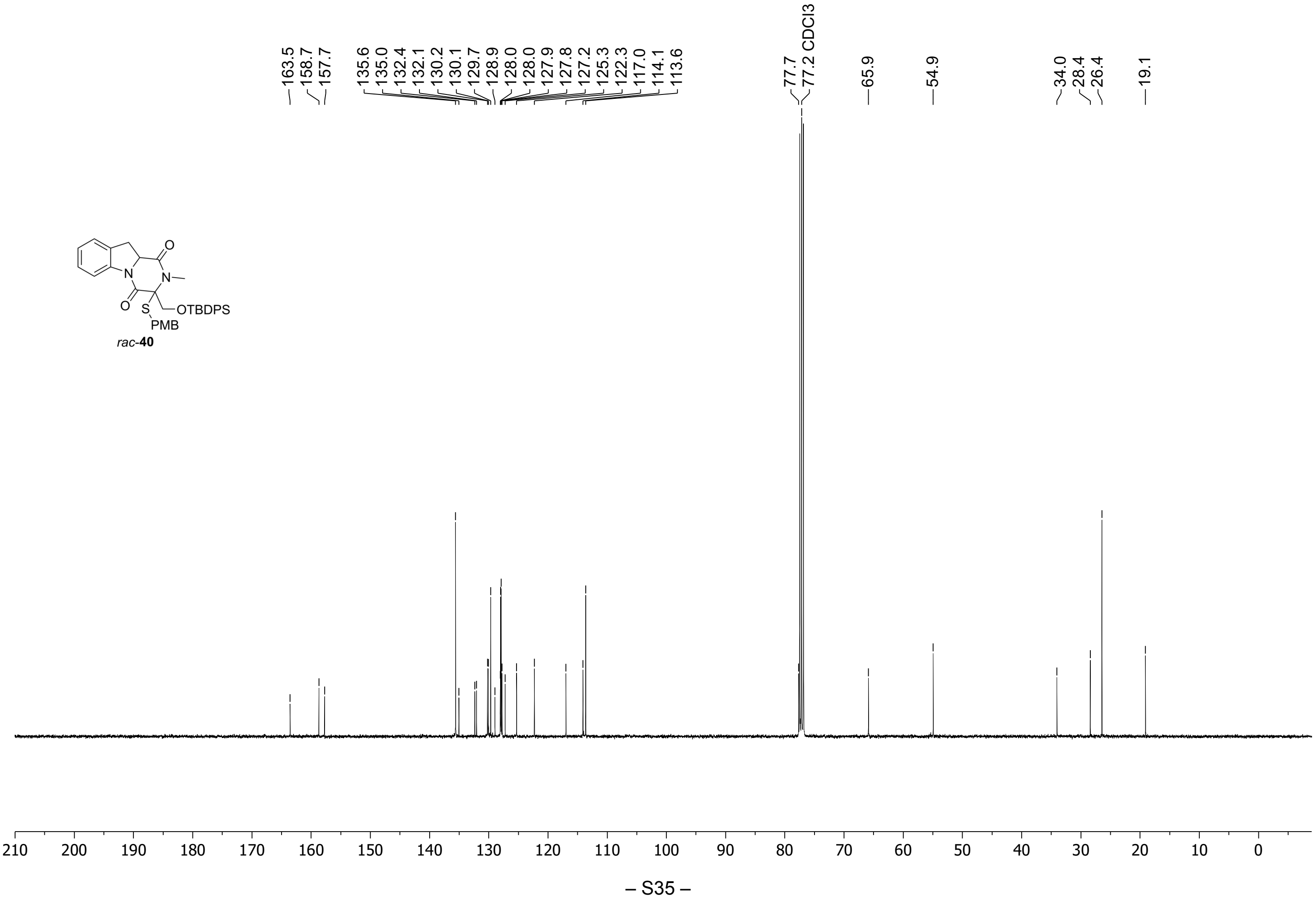

Supplement: Supplementary file 1 — ao2c00810_si_001.pdf [file ao2c00810_si_001.pdf]
